# Safety of ozanimod and etrasimod in ulcerative colitis: A disproportionality analysis of the Food and Drug Administration Adverse Event Reporting System database

**DOI:** 10.1097/MD.0000000000050262

**Published:** 2026-08-21

**Authors:** Long Chen, Dongping Wan, Benkai Xing, Xiaohui Ji, Wenyan Zhou, Yanbing Liu, Yongchao Lu

**Affiliations:** a The First Clinical Medical College, Shandong University of Traditional Chinese Medicine, Jinan, Shandong, China; b Postgraduate Education, Guangxi University of Chinese Medicine, Nanning, Guangxi, China; c Department of Traditional Chinese Medicine (Proctology Division), Shandong Provincial Hospital Affiliated to Shandong First Medical University, Jinan, Shandong, China.

**Keywords:** adverse event, etrasimod, FAERS, ozanimod, pharmacovigilance, sphingosine-1-phosphate receptor modulators, ulcerative colitis

## Abstract

Ozanimod and etrasimod are sphingosine-1-phosphate receptor modulators approved for ulcerative colitis treatment. Given their limited real-world safety data in this specific population, we conducted a disproportionality analysis using the Food and Drug Administration Adverse Event (AE) Reporting System database, aiming to explore their post-marketing safety profile. Original data were extracted from the aforementioned database. Three algorithms, namely reporting odds ratio, proportional reporting ratio, and Bayesian confidence propagation neural network, were employed to identify drug-related AE signals. We retrieved 1632 reports for ozanimod and 283 for etrasimod, with the most frequently reported age group being 50 to 59 years in both groups. At the system organ class level, signals for nervous system disorders, eye disorders, and cardiac disorders were detected for both drugs. At the preferred term level, 75 positive signals were identified for ozanimod and 24 for etrasimod. Common AEs for both drugs included drug ineffective, headache, dizziness, nausea, and blurred vision. Although most findings were consistent with the prescribing information, several unexpected AEs related to ozanimod merit special attention. The median onset time for ozanimod-related AEs was 17.5 days (interquartile range: 1.5–87.5 days). For etrasimod, the median onset time of AEs was 17.5 days (interquartile range: 2.75–69.25 days). We hope that these findings can provide comprehensive safety evidence for ozanimod and etrasimod, serving as a reference for clinicians in their individualized and safe application. However, due to the inherent limitations of the database, these findings still require further validation through prospective studies.

## 1. Introduction

Ulcerative colitis (UC) is a chronic, immune-mediated disease characterized by diffuse colonic mucosal inflammation with a relapsing-remitting course,^[1]^ affecting an estimated 5 million individuals globally.^[2]^ Its incidence continues to rise in both industrialized and developing countries, posing a significant health challenge.^[2,3]^ The primary goal of UC treatment is to induce and maintain clinical remission, which includes resolution of symptoms and achieving endoscopic healing.^[1,4]^ Despite an expanding range of therapeutic options such as aminosalicylates, corticosteroids, immunosuppressants, and biological agents, a substantial proportion of patients still experience inadequate response, loss of response, or intolerable adverse events (AEs).^[5,6]^ Consequently, approximately 10%–20% of UC patients ultimately require proctocolectomy due to poor response to currently available therapies,^[2]^ highlighting a strong unmet need for agents with novel mechanisms of action and favorable safety profiles.

The advent of new small molecules has transformed the treatment paradigm for immune-mediated inflammatory diseases like rheumatoid arthritis, multiple sclerosis (MS), and inflammatory bowel diseases.^[7]^ Among these, sphingosine-1-phosphate receptor (S1PR) modulators represent a mechanistic breakthrough.^[8–10]^ By functionally antagonizing S1P receptors on lymphocytes,^[11]^ these agents sequester lymphocytes within lymph nodes, thus preventing their egress into inflammation sites,^[12]^ such as the colonic mucosa. Ozanimod and etrasimod are 2 newer S1PR modulators, which have emerged for UC treatment in the past few years, demonstrating encouraging efficacy in inducing and maintaining remission.^[13–15]^ Ozanimod, a selective S1P_1_ and S1P_5_ modulator, was approved by the U.S. Food and Drug Administration (FDA) for moderate-to-severe UC in 2021, followed by etrasimod (targeting S1P_1,4,5_) in 2023, offering oral alternatives to biologic therapies.^[16,17]^

The safety profile of S1PR modulators has been established in the MS population, given that these agents were first widely adopted for this indication.^[9,10,18]^ However, it cannot be seamlessly extrapolated to UC patients. Unlike MS, UC is characterized by a chronic systemic inflammatory state, which is associated with the prevalence of cardiovascular risk factors,^[19]^ and elevates the risk of thromboembolism.^[20]^ Furthermore, UC patients often present with distinct comorbidities,^[21]^ concurrent hepatobiliary manifestations,^[22]^ and are frequently exposed to polypharmaceutical regimens, which may potentiate drug-drug interactions and precipitate additional health issues.^[23]^ Therefore, a disease-specific safety assessment is imperative to inform risk-benefit considerations for individuals with UC.

While pivotal randomized controlled trials have established the efficacy and tolerability of ozanimod and etrasimod in UC,^[14,15,24–30]^ their generalizability is inherently limited due to stringent inclusion criteria, limited follow-up duration, and insufficient capacity to identify rare AEs. Furthermore, real-world data on the safety of newer advanced therapies like S1PR modulators remain scarce,^[31]^ particularly in patients with UC.^[32]^ Post-marketing surveillance, therefore, plays an indispensable role in characterizing the comprehensive safety spectrum of these newer agents. The Food and Drug Administration Adverse Event Reporting System (FAERS) is a globally recognized repository for spontaneous AE reports submitted by healthcare professionals, consumers, pharmaceutical manufacturers, and other stakeholders. Established and maintained by the FDA, this publicly accessible platform facilitates the monitoring of post-marketing safety signals, providing a robust foundation for pharmacovigilance research.

To date, no pharmacovigilance study has specifically compared the AE profiles of ozanimod and etrasimod in the UC population using a real-world database. In this study, we leveraged the FAERS database to perform a disproportionality analysis of ozanimod and etrasimod related AEs in UC patients, aiming to provide clinicians with additional safety information to support the individualized application of S1PR modulators.

## 2. Methods

### 2.1. Data sources and preprocessing

**Figure 1.**
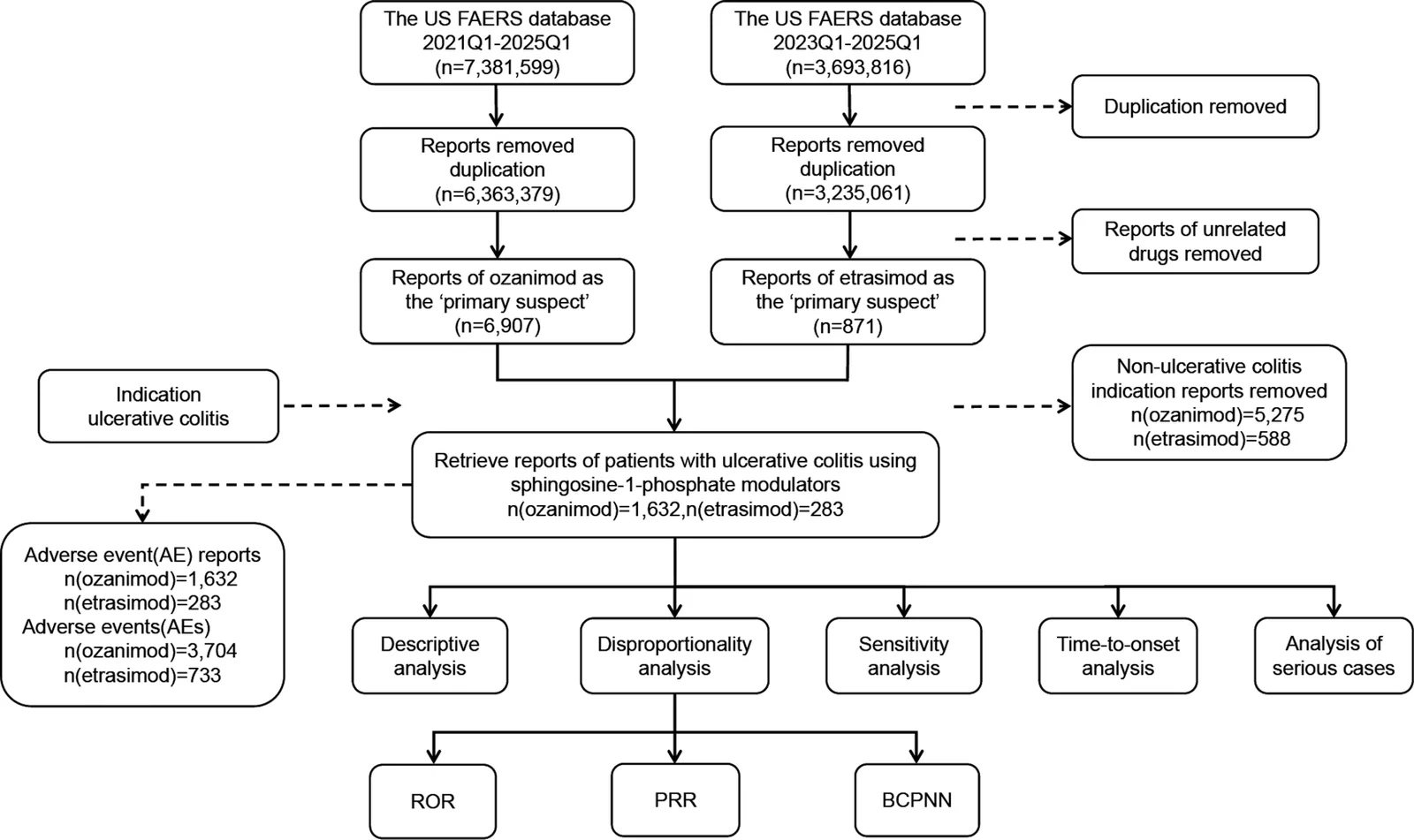
Flowchart of data extraction and filtering from the FAERS database for pharmacovigilance analysis of ozanimod and etrasimod in ulcerative colitis. 2025Q1 = the first quarter of 2025, BCPNN = Bayesian confidence propagation neural network, FAERS = the FDA Adverse Event Reporting System, PRR = proportional reporting ratio, ROR = reporting odds ratio.

The original data for this study were retrieved from the FAERS database, covering the period from the year of approval through the first quarter (Q1) of 2025 (ozanimod: Q1 2021-Q1 2025; etrasimod: Q1 2023-Q1 2025). The dataset comprised 7 file types: demographic information (DEMO), drug information (DRUG), coded adverse events (REAC), indications (INDI), patient medical outcomes (OUTC), start and end dates of the drug therapy (THER), and reporting sources (RPSR). Additionally, all adverse events were coded using the preferred term (PT) and system organ class (SOC) from the Medical Dictionary for Regulatory Activities (MedDRA, version 27.0). A single patient report could contain multiple AEs, which were recorded in the REAC file. We performed data deduplication in accordance with the approach recommended by the FDA.^[33]^ When 2 reports shared the same CASEID, we selected the one with the latest FDA_DT; in cases where both CASEID and FDA_DT were identical, the report with the higher PRIMARYID was retained. The overall study flow is illustrated in Figure 1. Ethics approval was waived for this study as it utilized only publicly available, anonymized data from the FAERS database.

### 2.2. Study design

During the screening process, we restricted our study population to patients with an indication of UC to control for the influence of underlying diseases.^[34]^ This approach allows us to address the clinically relevant question: when compared with other UC treatments, what additional AEs are linked to ozanimod and etrasimod among UC patients? By using a disease-specific comparator group, we partially controlled for confounding variables specific to UC, such as concomitant medication use and chronic systemic inflammation, which might otherwise skew signal detection if the entire FAERS database were used as background. In addition, only reports where the drug of interest was listed as the primary suspect (PS) were included. This study adhered to the Reporting of A Disproportionality analysis for drug safety signal detection using individual case safety reports in PharmacoVigilance (READUS-PV) guidelines to ensure methodological transparency, completeness, and accuracy.^[35,36]^ The relevant checklists were provided in Supplementary Tables S1 and S2, Supplemental Digital Content 1.

### 2.3. Statistical analysis

Disproportionality analysis was performed to detect safety signals for the target drugs.^[37]^ Based on the 4-grid contingency table (Table 1), we employed 2 frequentist methods and one Bayesian method, namely the reporting odds ratio (ROR), the proportional reporting ratio (PRR), and the Bayesian confidence propagation neural network.^[38]^ These algorithms identify potential AE signals by comparing the observed frequency of a given drug-AE pair with the frequency expected from all other reports in the entire database. The information component (IC) is a metric derived within the Bayesian framework as the base-two logarithm of the observed-to-expected ratio.^[39]^

**Table 1:** Formulas, signal detection criteria, and 4-grid contingency table for disproportionality analysis.

Algorithm	Formula	Criteria	
ROR	ROR = $\frac{ac}{bd}$	a ≥3, lower limit of 95%CI > 1	
	95%CI = $e^{ln\left(ROR\right)\pm \sqrt{\frac{1}{a}+\frac{1}{b}+\frac{1}{c}+\frac{1}{d}}}$		
PRR	PRR = $\frac{a/\left(a+c\right)}{b/\left(b+d\right)}$	PRR ≥ 2, a ≥3, χ^2^ ≥ 4	
	95%CI = $e^{ln\left(PRR\right)\pm \sqrt{\frac{1}{a}\;\text{--}\;\frac{1}{a+b}+\frac{1}{c}\;\text{--}\;\frac{1}{c+d}}}$		
	χ^2^= $\frac{\left(a+b+c+d\right)\left(ad\text{--}bc\right){\;}^{2}}{\left(a+b\right)\left(c+d\right)\left(a+c\right)\left(b+d\right)}$		
BCPNN	IC = $\log_{2}\frac{a\left(a+b+c+d\right)}{\left(a+c\right)\left(a+b\right)}$	IC025 > 0	
	95%CI = E(IC) ± 2 $\sqrt{V\left(IC\right)}$		
Four-grid contingency table			
	Target adverse event	Other adverse events	Total
Target drug	a	b	a +b
Other drugs	c	d	c + d
Total	a +c	b + d	a +b + c + d

a = number of reports of the target adverse event with the target drug, b = number of reports of other adverse events with the target drug, BCPNN = Bayesian confidence propagation neural network, c = number of reports of the target adverse event with other drugs, CI = confidence interval, d = number of reports of other adverse events with other drugs, IC = information component, IC025 = the lower limit of 95% confidence interval of information component, PRR = proportional reporting ratio, ROR = reporting odds ratio, *χ*^2^ = chi-squared.

In this study, we evaluated potential associations by comparing the frequency of AEs reported with drugs of interest against that of other reports within the UC population during the same period. Detailed algorithms, signal detection criteria, and the 4-grid contingency table are provided in Table 1. A drug-related AE signal was considered positive only if it concurrently met the criteria of all 3 methods: for ROR, a **≥** 3 and the lower limit of the 95% confidence interval **>** 1; for PRR, a **≥** 3 and PRR **≥** 2 with a corresponding χ^2^ **≥** 4; for Bayesian confidence propagation neural network, the lower limit of the 95% confidence interval of the IC (IC025) **>** 0. This combined approach reduced the likelihood of false-positive findings. To improve the specificity of the analysis, we excluded signals related to product issues, usage issues (e.g., abuse, misuse), and those clearly unrelated to the drug effect. All data processing, statistical analysis, and visualization were conducted using R (version 4.3.3), Microsoft Excel (2019), and Microsoft PowerPoint (2019).

### 2.4. Sensitivity analysis

To further assess the potential impact of background selection on signal detection and to evaluate whether restricting to UC reports may lead to over- or underestimation of AE signals, we conducted a sensitivity analysis using the entire FAERS database as the comparator. In this sensitivity analysis, all reports with ozanimod or etrasimod as the PS drug were compared against all other reports in the FAERS database, using the same disproportionality algorithms and signal detection criteria. Additionally, to evaluate the robustness of the findings, we performed 2 additional sensitivity analyses. First, we restricted the analysis to reports submitted by healthcare professionals (including physicians, pharmacists, registered nurses, and other healthcare professionals). Second, for each target drug, we excluded reports containing any of the top 30 most frequently co-administered drugs (Supplementary Table S3, Supplemental Digital Content 2) or any of the commonly used immunosuppressants in UC (Supplementary Table S4, Supplemental Digital Content 3), and then repeated the disproportionality analysis.

### 2.5. Time-to-onset (TTO) analysis

The onset time of drug-related AEs was calculated as EVENT_DT minus START_DT plus 0.5, where EVENT_DT and START_DT represent the date of AE occurrence and the date of drug initiation, respectively, as recorded in the DEMO and THER files. For reports with only the year and month available, the onset date was imputed as the 15th day of the month. Reports with incomplete data, input errors (i.e., EVENT_DT earlier than START_DT), or those reporting only the year were excluded from the TTO analysis.

### 2.6. Analysis of serious cases

To characterize the clinical features and patterns of serious cases identified in the FAERS database, we conducted a case‑by‑case analysis. A serious AE was defined according to the standard regulatory definition, encompassing any adverse medical event that resulted in death, was life‑threatening, required hospitalization or prolongation of existing hospitalization, caused a congenital anomaly or birth defect, or led to persistent or significant disability or incapacity.^[24]^ Reports containing at least one serious AE were classified as serious cases.

## 3. Results

### 3.1. Descriptive analysis

Following data processing, we identified 1632 reports with ozanimod and 283 reports with etrasimod as the PS drug, respectively. These reports contained 3704 AEs for ozanimod and 733 AEs for etrasimod. Supplementary Table S5, Supplemental Digital Content 4 summarizes the specific drug information retrieved from the database, detailing the brand names, active ingredients, and report frequency for each entry. Table 2 presents the demographic characteristics of patients in the included reports. Females were slightly more prevalent in both groups (50.92% for ozanimod and 52.30% for etrasimod). The median age was 48 years (interquartile range [IQR]: 33–61) for ozanimod and 48 years (IQR: 36–59) for etrasimod, with the most frequently reported age group being 50 to 59 years in both groups. The United States was the largest contributor of cases in both cohorts, accounting for 1573 (96.38%) reports in the ozanimod group and 247 (87.28%) in the etrasimod group. The majority of reports were submitted by healthcare professionals for both ozanimod (n = 1048; 64.22%) and etrasimod (n = 190; 67.14%).

**Table 2:** Demographic characteristics of adverse event reports of ozanimod and etrasimod in ulcerative colitis population reported in the Food and Drug Administration Adverse Event Reporting System database.

Characteristics	Ozanimod, n(%)	Etrasimod, n(%)
Number of reports	1632	283
Gender		
Female	831 (50.92%)	148 (52.30%)
Male	751 (46.02%)	119 (42.05%)
missing	50 (3.06%)	16 (5.65%)
Age(yr)		
missing	277 (16.97%)	94 (33.22%)
<18	17 (1.04%)	4 (1.41%)
18–29	236 (14.46%)	22 (7.77%)
30–39	225 (13.79%)	33 (11.66%)
40–49	238 (14.58%)	41 (14.49%)
50–59	273 (16.73%)	48 (16.96%)
60–69	232 (14.22%)	24 (8.48%)
≥70	134 (8.21%)	17 (6.01%)
Median(IQR)	48 (33,61)	48 (36,59)
Reporter		
Consumer	582 (35.66%)	93 (32.86%)
Healthcare Professionals	1048 (64.22%)	190 (67.14%)
missing	2 (0.12%)	0 (0.00%)
Outcomes		
Death	14 (0.86%)	0 (0.00%)
Disability	6 (0.37%)	1 (0.35%)
Hospitalization	128 (7.84%)	23 (8.13%)
Life-Threatening	15 (0.92%)	0 (0.00%)
Other	1469 (90.01%)	259 (91.52%)
Reported countries(Top3)		
1	US 1573 (96.38%)	US 247 (87.28%)
2	DE 15 (0.92%)	CA 28 (9.89%)
3	CA 9 (0.55%)	AT 2 (0.71%)
Reporting yr		
2021	206 (12.62%)	NA
2022	514 (31.50%)	NA
2023	432 (26.47%)	1 (0.35%)
2024	400 (24.51%)	180 (63.61%)
2025Q1	80 (4.90%)	102 (36.04%)

2025Q1 = the first quarter of 2025, AT = Austria, CA = Canada, DE = Germany, IQR = interquartile range, US = the United States.

### 3.2. Signal detection

#### 3.2.1. AE signals at the SOC level

**Figure 2.**
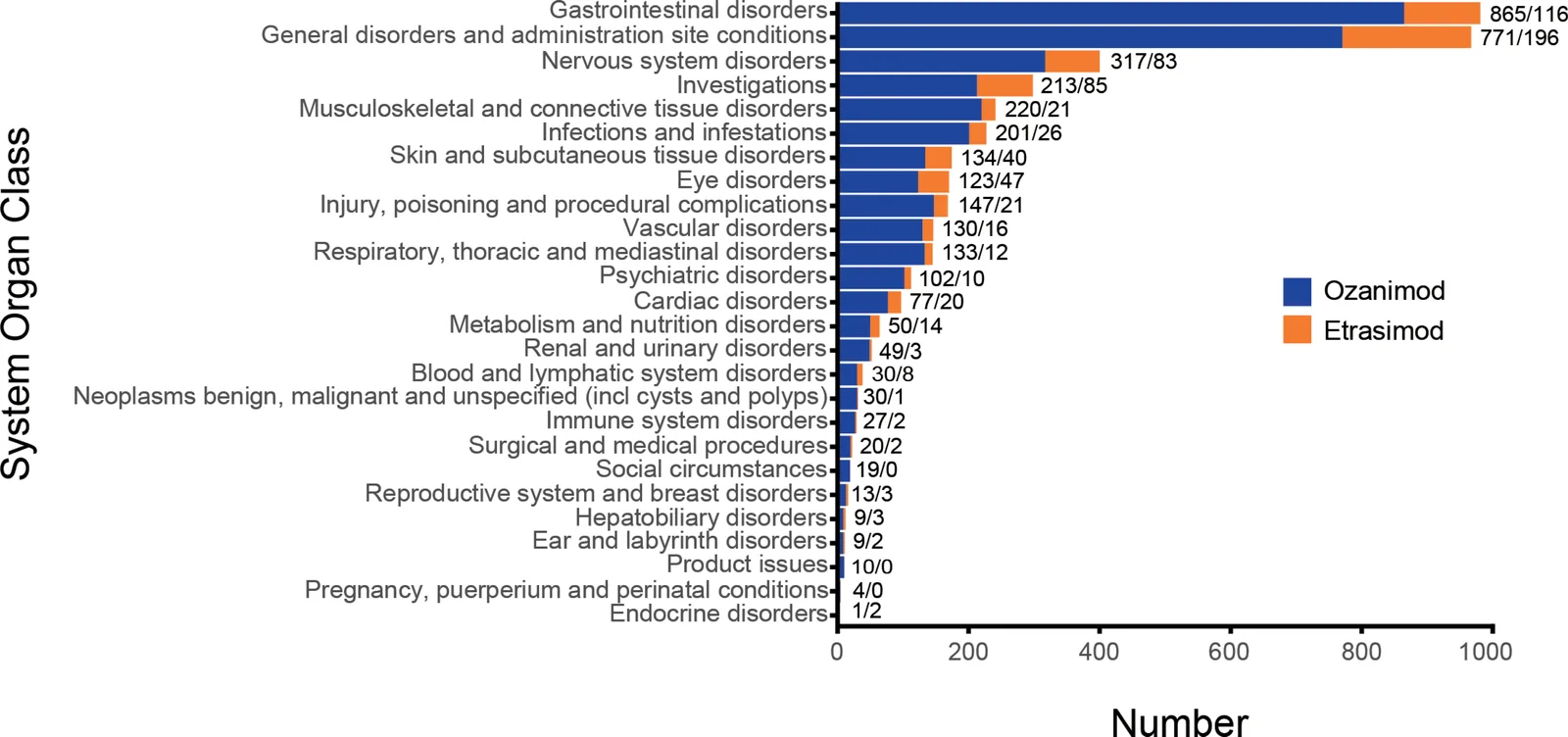
Distribution of potential adverse event signals for ozanimod and etrasimod at the system organ class level before signal detection.

We performed signal detection for AEs associated with ozanimod and etrasimod. Figure 2 presents the distribution of AEs for both drugs at the SOC level prior to signal detection. Ozanimod-related potential AEs involved 26 SOCs, whereas etrasimod-related AEs involved 23. Following signal detection, 3 positive signals were identified for ozanimod: nervous system disorders (ROR = 2.12), eye disorders (ROR = 3.49), and cardiac disorders (ROR = 2.20). Similarly, the positive signals for etrasimod were nervous system disorders (ROR = 2.84), eye disorders (ROR = 6.87), and cardiac disorders (ROR = 2.91). Table 3 presents the strength of AE signals associated with ozanimod and etrasimod at the SOC level. These findings suggest that nervous system disorders, eye disorders, and cardiac disorders may be associated with S1PR modulator use and warrant consideration in the clinical management of UC.

**Table 3:** Strength of adverse event signals of ozanimod and etrasimod at the system organ class level.

SOC	Ozanimod					Etrasimod				
	n	ROR(95%CI)	PRR(95%CI)	χ^2^	IC(IC025)	n	ROR(95%CI)	PRR(95%CI)	χ^2^	IC (IC025)
Gastrointestinal disorders	865	1.25 (1.16, 1.35)	1.19 (1.14,1.25)	33.72	0.25 (0.14)	116	0.76 (0.62,0.93)	0.80 (0.63,0.97)	7.34	−0.32 (−0.61)
General disorders and administration site conditions	771	1.20 (1.11, 1.30)	1.16 (1.10,1.22)	20.82	0.21 (0.10)	196	1.76 (1.50,2.08)	1.56 (1.44,1.68)	47.29	0.64 (0.41)
** Nervous system disorders** *	317	2.12 (1.89, 2.38)	2.02 (1.92,2.13)	168.13	1.00 (0.83)	83	2.84 (2.26,3.57)	2.63 (2.43,2.84)	87.11	1.39 (1.06)
Musculoskeletal and connective tissue disorders	220	1.15 (1.00, 1.31)	1.14 (1.01,1.27)	3.82	0.18 (−0.02)	21	0.51 (0.33,0.79)	0.53 (0.10,0.95)	9.51	−0.93 (−1.55)
Investigations	213	0.76 (0.66, 0.87)	0.77 (0.64,0.91)	15.04	−0.37 (−0.57)	85	1.75 (1.39,2.19)	1.66 (1.46,1.86)	23.82	0.73 (0.40)
Infections and infestations	201	0.69 (0.60,0.80)	0.71 (0.57,0.84)	26.23	−0.5 (−0.7)	26	0.41 (0.28,0.61)	0.44 (0.06,0.81)	20.69	−1.2 (−1.76)
Injury, poisoning and procedural complications	147	0.23 (0.20,0.27)	0.26 (0.10,0.42)	356.10	−1.92 (−2.16)	21	0.17 (0.11,0.25)	0.19 (−0.23,0.61)	86.07	−2.4 (−3.02)
Skin and subcutaneous tissue disorders	134	0.94 (0.79,1.12)	0.95 (0.78,1.11)	0.43	−0.08 (−0.33)	40	1.43 (1.04,1.96)	1.40 (1.10,1.70)	4.78	0.49 (0.02)
Respiratory, thoracic and mediastinal disorders	133	1.06 (0.89,1.26)	1.06 (0.89,1.23)	0.46	0.08 (−0.17)	12	0.46 (0.26,0.82)	0.47 (−0.09,1.03)	7.36	−1.08 (−1.89)
Vascular disorders	130	1.77 (1.49,2.11)	1.74 (1.57,1.92)	41.45	0.79 (0.53)	16	1.19 (0.73,1.96)	1.19 (0.70,1.67)	0.49	0.25 (−0.46)
**Eye disorders** *	123	3.49 (2.91,4.19)	3.41 (3.23,3.59)	204.30	1.73 (1.47)	47	6.87 (5.10,9.26)	6.49 (6.21,6.77)	216.25	2.67 (2.24)
Psychiatric disorders	102	1.16 (0.95,1.41)	1.15 (0.96,1.34)	2.06	0.20 (−0.09)	10	0.54 (0.29,1.02)	0.55 (−0.07,1.17)	3.76	−0.86 (−1.74)
**Cardiac disorders** *	77	2.20 (1.75,2.77)	2.18 (1.95,2.40)	48.42	1.11 (0.77)	20	2.91 (1.86,4.55)	2.86 (2.42,3.29)	24.18	1.51 (0.87)
Metabolism and nutrition disorders	50	0.97 (0.73,1.28)	0.97 (0.69,1.24)	0.06	−0.05 (−0.46)	14	1.33 (0.78,2.26)	1.33 (0.81,1.85)	1.14	0.41 (−0.35)
Renal and urinary disorders	49	1.79 (1.34,2.37)	1.77 (1.49,2.06)	16.40	0.82 (0.40)	3	0.56 (0.18,1.74)	0.56 (−0.57,1.69)	1.04	−0.83 (−2.28)
Neoplasms benign, malignant and unspecified (incl cysts and polyps)	30	0.71 (0.49,1.02)	0.71 (0.35,1.07)	3.52	−0.49 (−1.01)	1	0.12 (0.02,0.87)	0.12 (−1.84,2.08)	6.29	−3.01 (−5.06)
Blood and lymphatic system disorders	30	1.29 (0.90,1.85)	1.29 (0.93,1.65)	1.93	0.36 (−0.16)	8	1.77 (0.88,3.57)	1.76 (1.07,2.46)	2.65	0.82 (−0.15)
Immune system disorders	27	0.98 (0.67,1.44)	0.98 (0.60,1.36)	0.01	−0.03 (−0.58)	2	0.36 (0.09,1.44)	0.36 (−1.02,1.75)	2.28	−1.47 (−3.14)
Surgical and medical procedures	20	0.39 (0.25,0.61)	0.39 (−0.04,0.83)	18.88	−1.34 (−1.97)	2	0.21 (0.05,0.84)	0.21 (−1.17,1.60)	5.89	−2.23 (−3.9)
Social circumstances	19	0.94 (0.60,1.48)	0.94 (0.49,1.39)	0.06	−0.08 (−0.73)	0	–	–	–	–
Reproductive system and breast disorders	13	0.92 (0.53,1.59)	0.92 (0.37,1.46)	0.09	−0.12 (−0.9)	3	0.96 (0.31,3.00)	0.97 (−0.17,2.10)	0.00	−0.05 (−1.5)
Product issues	10	0.33 (0.18,0.61)	0.33 (−0.29,0.95)	13.85	−1.6 (−2.47)	0	–	–	–	–
Hepatobiliary disorders	9	0.39 (0.20,0.76)	0.40 (−0.26,1.05)	8.32	−1.33 (−2.24)	3	0.63 (0.20,1.96)	0.63 (−0.50,1.76)	0.65	−0.66 (−2.11)
Ear and labyrinth disorders	9	0.74 (0.38,1.43)	0.74 (0.09,1.40)	0.80	−0.43 (−1.34)	2	0.71 (0.18,2.86)	0.71 (−0.67,2.10)	0.23	−0.49 (−2.16)
Pregnancy, puerperium and perinatal conditions	4	0.23 (0.09,0.62)	0.23 (−0.75,1.22)	10.02	−2.08 (−3.37)	0	–	–	–	–
Endocrine disorders	1	0.27 (0.04,1.93)	0.27 (−1.69,2.23)	1.96	−1.87 (−3.92)	2	2.94 (0.73,11.86)	2.94 (1.55,4.33)	2.53	1.55 (−0.13)

Abbreviations: CI = confidence interval, IC = information component, IC025 = the lower limit of the 95% confidence interval of information component, PRR = proportional reporting ratio, ROR = reporting odds ratio, SOC = system organ class, χ^2^ = chi-squared.

Note: SOCs in **bold** indicate positive signals for ozanimod, while those marked with an asterisk (*) indicate positive signals for etrasimod. Both signals met all 3 detection algorithms.

#### 3.2.2. AE signals at the PT level

We employed the 3 algorithms and identified 75 positive signals for ozanimod and 24 for etrasimod at the PT level. The resulting signals and their corresponding strengths are summarized in Table 4 (featuring the top 30 signals for ozanimod and all signals for etrasimod) and provided in full in Supplementary Table S6, Supplemental Digital Content 5. For ozanimod, the 3 most frequently reported signals were drug ineffective (ROR = 2.35), fatigue (ROR = 2.38), and headache (ROR = 2.49). Macular edema (ROR = 116.07) was the strongest signal, followed by mental status changes (ROR = 48.97) and decreased lymphocyte count (ROR = 43.35). For etrasimod, the 3 most frequent signals were drug ineffective (ROR = 3.85), headache (ROR = 3.42), and dizziness (ROR = 6.46), while the strongest signals by ROR were lymphocyte count decreased (ROR = 61.97), macular edema (ROR = 56.72), and bradycardia (ROR = 36.45). Notably, several unexpected AEs were identified for ozanimod, including tremor (ROR = 2.37), brain fog (ROR = 7.07), and balance disorder (ROR = 2.66).

**Table 4:** Positive adverse event signals and corresponding strengths of ozanimod (top 30 in cases) and etrasimod at the preferred term level.

No	Ozanimod						Etrasimod					
	PT	n(%)	ROR(95%Cl)	PRR(95%CI)	χ^2^	IC(IC025)	PT	n(%)	ROR(95%Cl)	PRR(95%CI)	χ^2^	IC(IC025)
1	Drug ineffective	201	2.35	2.27	143.46	1.17	Drug ineffective	57	3.85	3.62	109.44	1.85
		(15.09)	(2.03, 2.71)	(2.14, 2.41)		(0.96)		(24.36)	(2.93, 5.05)	(3.37, 3.87)		(1.45)
2	Fatigue	125	2.38	2.33	94.54	1.20	Headache	25	3.42	3.34	41.01	1.73
		(9.38)	(1.99, 2.85)	(2.16, 2.51)		(0.94)		(10.68)	(2.29, 5.11)	(2.95, 3.73)		(1.15)
3	Headache	91	2.49	2.46	77.43	1.28	Dizziness	24	6.46	6.28	105.08	2.63
		(6.83)	(2.02, 3.08)	(2.25, 2.66)		(0.97)		(10.26)	(4.28, 9.74)	(5.88, 6.68)		(2.04)
4	Haemorrhage	66	5.83	5.75	245.22	2.46	Nausea	17	2.84	2.79	19.57	1.47
		(4.95)	(4.54, 7.49)	(5.50, 5.99)		(2.09)		(7.26)	(1.75, 4.60)	(2.32, 3.27)		(0.78)
5	Nausea	64	2.03	2.01	32.21	0.99	Vision blurred	13	20.16	19.82	218.93	4.23
		(4.80)	(1.58, 2.61)	(1.77, 2.26)		(0.63)		(5.56)	(11.46, 35.46)	(19.26, 20.37)		(3.42)
6	Dizziness	60	3.02	2.99	77.61	1.55	Pain	12	2.13	2.11	6.99	1.07
		(4.50)	(2.33, 3.92)	(2.74, 3.25)		(1.17)		(5.13)	(1.20, 3.77)	(1.54, 2.67)		(0.26)
7	Back pain	45	3.65	3.62	82.64	1.82	Therapeutic product ef-	11	5.01	4.95	34.27	2.29
		(3.38)	(2.71, 4.93)	(3.33, 3.92)		(1.38)	fect incomplete	(4.70)	(2.75, 9.13)	(4.36, 5.54)		(1.44)
8	Dyspnoea	42	2.39	2.38	32.88	1.23	Bradycardia	8	36.45	36.07	245.22	5.02
		(3.15)	(1.76, 3.26)	(2.07, 2.68)		(0.78)		(3.42)	(17.49, 75.99)	(35.34, 36.79)		(4.00)
9	Abdominal pain upper	36	2.26	2.25	24.50	1.15	Hypertension	6	2.46	2.45	5.11	1.28
		(2.70)	(1.62, 3.15)	(1.92, 2.58)		(0.67)		(2.56)	(1.10, 5.51)	(1.65, 3.25)		(0.18)
10	Vision blurred	31	9.26	9.19	207.15	3.09	Visual impairment	5	8.26	8.21	30.88	3.00
		(2.33)	(6.40, 13.41)	(8.83, 9.56)		(2.55)		(2.14)	(3.39, 20.12)	(7.32, 9.09)		(1.80)
11	Hypertension	29	2.54	2.53	26.23	1.32	Hypotension	5	6.29	6.25	21.66	2.62
		(2.18)	(1.76, 3.68)	(2.16, 2.90)		(0.78)		(2.14)	(2.59, 15.28)	(5.37, 7.13)		(1.42)
12	Palpitations	25	5.13	5.10	78.37	2.29	Lymphocyte count dec-	5	61.97	61.56	249.86	5.69
		(1.88)	(3.42, 7.67)	(4.70, 5.50)		(1.71)	reased	(2.14)	(23.73, 161.84)	(60.60, 62.51)		(4.41)
13	Peripheral swelling	23	2.55	2.54	20.94	1.32	Influenza like illness	5	5.23	5.20	16.70	2.36
		(1.73)	(1.68, 3.86)	(2.13, 2.95)		(0.72)		(2.14)	(2.15, 12.68)	(4.32, 6.08)		(1.16)
14	Muscle spasms	23	2.58	2.57	21.54	1.34	Type 2 diabetes mellit-	5	4.89	4.86	15.14	2.27
		(1.73)	(1.70, 3.91)	(2.16, 2.98)		(0.74)	us	(2.14)	(2.02, 11.87)	(3.98, 5.74)		(1.07)
15	Visual impairment	23	7.05	7.01	110.74	2.72	Gastrooesophageal ref-	4	6.60	6.57	18.51	2.69
		(1.73)	(4.61, 10.78)	(6.59, 7.43)		(2.11)	lux disease	(1.71)	(2.44, 17.80)	(5.58, 7.55)		(1.38)
16	Flatulence	20	2.00	2.00	9.79	0.98	White blood cell count	4	13.27	13.20	43.34	3.67
		(1.50)	(1.28, 3.12)	(1.56, 2.44)		(0.35)	decreased	(1.71)	(4.87, 36.16)	(12.20, 14.20)		(2.34)
17	Chest pain	18	2.10	2.09	10.11	1.05	Chills	4	3.04	3.03	5.39	1.59
		(1.35)	(1.32, 3.35)	(1.63, 2.56)		(0.38)		(1.71)	(1.13, 8.15)	(2.04, 4.01)		(0.29)
18	Disturbance in	18	23.92	23.81	316.40	4.27	Hepatic enzyme	4	2.89	2.88	4.87	1.52
	attention	(1.35)	(14.27, 40.07)	(23.29, 24.32)		(3.55)	increased	(1.71)	(1.08, 7.75)	(1.90, 3.86)		(0.21)
19	White blood cell count	17	12.29	12.23	155.94	3.46	Palpitations	4	3.62	3.61		1.84
	decreased	(1.28)	(7.41, 20.36)	(11.73, 12.74)		(2.74)		(1.71)	(1.35, 9.73)	(2.62, 4.59)	7.46	(0.54)
20	Maternal exposure during	17	2.01	2.00	8.41	0.99	Loss of therapeutic res-	4	2.73	2.72	4.34	1.44
	pregnency	(1.28)	(1.24, 3.25)	(1.53, 2.48)		(0.30)	ponse	(1.71)	(1.02, 7.33)	(1.74, 3.71)		(0.14)
21	Feeling abnormal	16	2.38	2.37	12.43	1.23	Blood iron decreased	3	5.54	5.52	10.92	2.44
		(1.20)	(1.45, 3.91)	(1.88, 2.87)		(0.52)		(1.28)	(1.76, 17.38)	(4.38, 6.66)		(0.98)
22	Hepatic enzyme increased	16	2.67	2.66	16.14	1.39	Alanine aminotransfer-	3	17.21	17.15	43.31	4.03
		(1.20)	(1.62, 4.38)	(2.16, 3.15)		(0.68)	ase increased	(1.28)	(5.38, 55.12)	(15.99, 18.31)		(2.54)
23	Myalgia	15	2.01	2.00	7.40	0.99	Macular edema	3	56.72	56.49	139.00	5.59
		(1.13)	(1.20, 3.35)	(1.49, 2.51)		(0.26)		(1.28)	(16.59, 193.95)	(55.26, 57.71)		(4.01)
24	Lymphocyte count decrea-	15	43.35	43.18	428.88	4.92	Eye pain	3	13.97	13.92	34.48	3.74
	sed	(1.13)	(23.59, 79.65)	(42.57, 43.78)		(4.09)		(1.28)	(4.39, 44.49)	(12.76, 15.07)		(2.26)
25	Pollakiuria	14	13.48	13.43	141.71	3.58						
		(1.05)	(7.70, 23.59)	(12.88, 13.99)		(2.78)						
26	Cerebrovascular accident	14	3.15	3.14	19.84	1.62						
		(1.05)	(1.85, 5.37)	(2.61, 3.67)		(0.86)						
27	Confusional state	13	2.46	2.45	10.91	1.27						
		(0.98)	(1.42, 4.26)	(1.90, 3.00)		(0.49)						
28	Macular edema	13	116.07	115.66	677.35	5.74						
		(0.98)	(51.96, 259.25)	(114.86, 116.47)		(4.78)						
29	Bowel movement	12	4.24	4.22	28.34	2.03						
	irregularity	(0.90)	(2.37, 7.55)	(3.65, 4.80)		(1.21)						
30	Influenza like illness	11	2.50	2.50	9.65	1.30						
		(0.83)	(1.37, 4.56)	(1.90, 3.10)		(0.45)						

CI = confidence interval, IC = information component, IC025 = the lower limit of the 95% confidence interval of information component, PRR = proportional reporting ratio, PT = preferred term, ROR = reporting odds ratio, χ^2^ = chi-squared.

### 3.3. Sensitivity analysis

When the entire FAERS database was used as the comparator, the pattern of AE signals showed discrepancies compared with the primary analysis. Detailed results of the full-database analysis are provided in Supplementary Tables S7 and S8, Supplemental Digital Content 6. At the SOC level, the signal profile differed. For ozanimod, only nervous system disorders (ROR = 2.95) remained positive. For etrasimod, positive signals were observed for gastrointestinal disorders (ROR = 2.77), eye disorders (ROR = 4.00), and metabolism and nutrition disorders (ROR = 2.63). At the PT level, 46 of the 75 signals (61.33%) for ozanimod and 15 of the 24 signals (62.50%) for etrasimod remained positive in the full-database analysis. Notably, the unexpected signals identified for ozanimod all persisted in the sensitivity analysis (tremor, ROR = 2.02; brain fog, ROR = 2.99; balance disorder, ROR = 5.49), supporting their robustness as potential drug-related AEs.

When the analysis was restricted to reports submitted by healthcare professionals, the 3 positive SOC level signals, namely nervous system, eye, and cardiac disorders, were reproduced for both drugs (Supplementary Table S9, Supplemental Digital Content 7). At the PT level, 60 positive signals were identified for ozanimod and 16 for etrasimod, of which 53 (88.3%) and 15 (93.8%) were consistent with the primary analysis, respectively (Supplementary Table S10, Supplemental Digital Content 8). After excluding reports containing any of the top 30 concomitant medications for each drug or the commonly used immunosuppressants in UC, the 3 SOC level signals again remained positive for both drugs (Supplementary Table S11, Supplemental Digital Content 9). At the PT level, 52 signals were detected for ozanimod and 16 for etrasimod, with 49 (94.2%) and 13 (81.3%) overlapping with the primary analysis (Supplementary Table S12, Supplemental Digital Content 10). Together, these sensitivity analyses demonstrate consistent patterns with the primary findings, further supporting the robustness of our results.

### 3.4. TTO analysis

**Figure 3.**
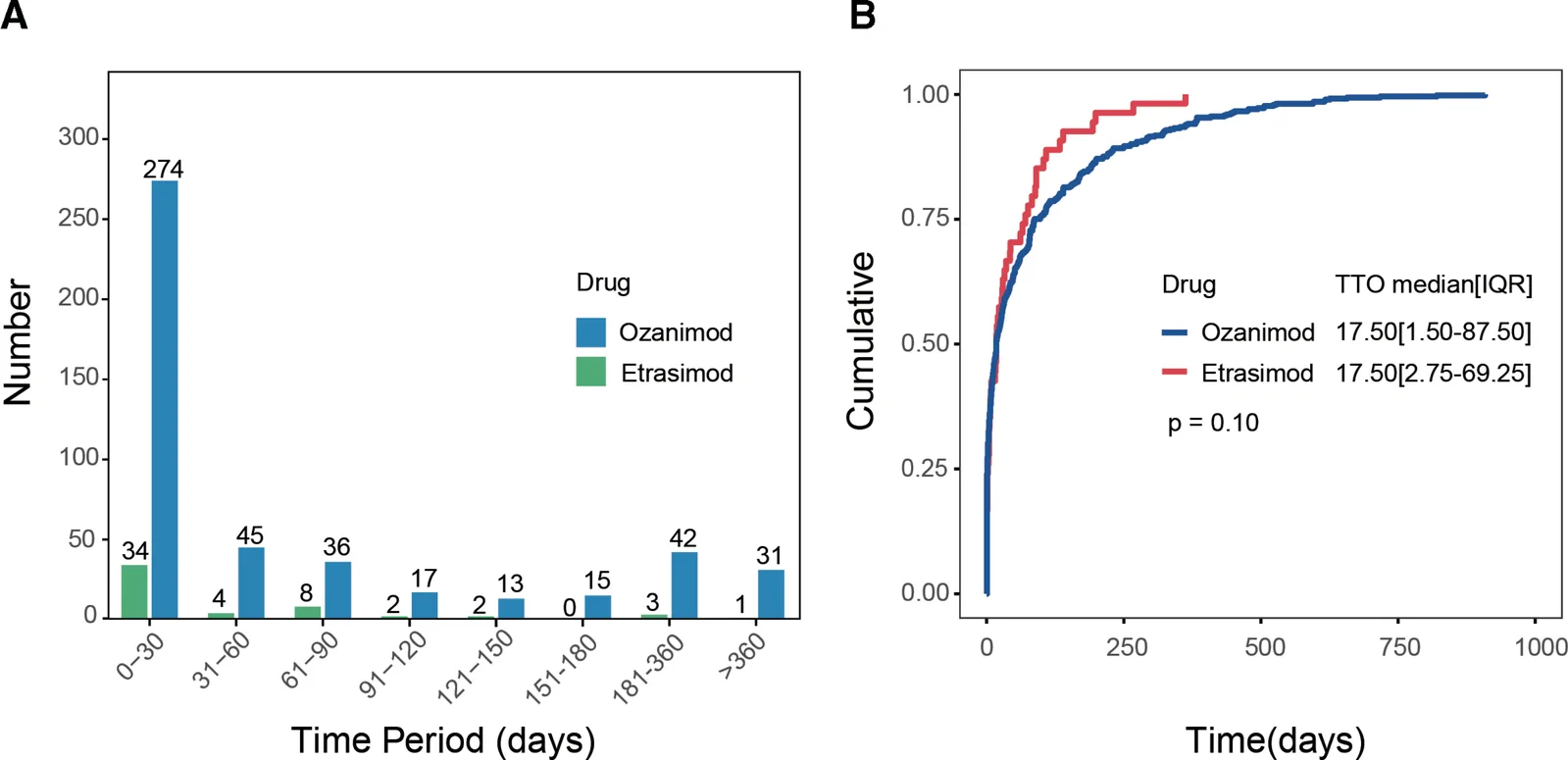
The onset time and cumulative incidence of adverse events related to ozanimod and etrasimod. (A) The onset time distribution of adverse events associated with these 2 drugs. (B) Cumulative incidence of adverse events associated with both drugs over time. IQR = interquartile range, TTO = time-to-onset.

After excluding reports with missing or erroneous data, 473 onset time records were available for ozanimod and 54 for etrasimod. The median onset time of AEs was 17.5 days (IQR: 1.5–87.5 days) for ozanimod and 17.5 days (IQR: 2.75–69.25 days) for etrasimod. Figure 3A illustrates the onset time distribution, showing that the majority of AEs occurred within the first month of therapy (ozanimod: 57.93%; etrasimod: 62.96%). As depicted in Figure 3B, the cumulative hazard curves indicate no significant difference in the cumulative incidence of AEs between the 2 drugs (log-rank test, *P* = .10). The Weibull distribution analysis (Table 5) suggests an early failure model for both drugs, indicating that the hazard of AE occurrence decreased over time during treatment.

**Table 5:** Time-to-onset analysis for ozanimod and etrasimod.

Drug	Case	TTO (d)		Weibull model				Failure type
				Scale parameter		Shape parameter		
	n	Median(IQR)	Min-max	α	95%CI	β	95%CI	
Ozanimod	473	17.50 (1.50–87.50)	0.50–908.50	41.92	33.86–49.97	0.50	0.46–0.53	Early Failure
Etrasimod	54	17.50 (2.75–69.25)	0.50–362.50	31.94	16.87–47.01	0.60	0.47–0.72	Early Failure

The Weibull distribution model is described by 2 parameters: the scale parameter (α) and the shape parameter (β). The shape parameter (β) is used to evaluate the frequency of adverse events over time. When β < 1 and the upper limit of the 95% CI is < 1, it indicates an early failure type, meaning that the frequency of adverse events is considered to initially increase and then gradually decrease over time. When β = 1 and the 95% CI includes 1, it belongs to the random failure type, meaning that the adverse events occur constantly throughout the entire treatment period. Conversely, when β > 1 and the lower limit of the 95% CI > 1, it represents a wear-out failure model, indicating that the frequency of adverse events increases as treatment progresses.

CI = confidence interval, IQR = interquartile range, TTO = time-to-onset, α = scale parameter of Weibull model, β = shape parameter of Weibull model.

### 3.5. Characteristics of serious cases

Four types of serious outcomes were identified: death, life-threatening events, hospitalization, and disability. Among the 163 serious cases associated with ozanimod, hospitalization was the most common outcome (78.53%), followed by life-threatening events (9.20%), death (8.59%), and disability (3.68%). The median age of these patients was 50 years (IQR: 34–63 years), and the median onset time was 46.5 days (IQR: 10.5–198.5 days). In contrast, etrasimod was associated with 24 serious cases, the majority of which involved hospitalization (95.83%); one case of disability (4.17%) was reported, with no deaths or life-threatening outcomes. The median age was 52 years. TTO analysis was not performed for etrasimod due to limited data. Detailed characteristics are provided in Supplementary Tables S13 and S14, Supplemental Digital Content 11.

## 4. Discussion

UC is a chronic, incurable disease with a relatively low mortality rate.^[2]^ Treatment aims to maintain sustained remission and reduce disease complications.^[16]^ Although biologic agents have revolutionized UC management over the past 2 decades, a considerable proportion of patients do not respond or lose response to these therapies.^[5]^ Traditional immunosuppressants are generally associated with limited efficacy, nonselective immunosuppressive action, and long-term safety concerns.^[8]^ This has driven the development of small molecule drugs with improved cost‑effectiveness, greater efficacy, and tolerability.^[40]^ S1PR modulators offer advantages of oral administration, rapid onset, short half-life, and no immunogenicity risk, making them a promising alternative for UC.^[41,42]^

S1P is a bioactive sphingolipid that exerts biological effects through 5 G-protein-coupled receptors (S1P_1_-_5_) expressed on various cell types,^[43]^ among which the S1P-S1P_1_ interaction plays a critical role in lymphocyte migration to sites of inflammation.^[11,44]^ S1PR modulators act as functional antagonists by inducing internalization and degradation of S1P_1_ on lymphocytes, thereby disrupting S1P gradient sensing and lymphocyte egress.^[12,44–47]^ Among these, ozanimod exhibits high affinity for S1P_1_ and S1P_5_, whereas etrasimod binds with high affinity to S1P_1_, S1P_4_, and S1P_5_.^[41,48,49]^ Since the approval of ozanimod in 2021 as the first S1PR modulator for UC, this class of agents has attracted increasing clinical attention. A systematic review confirmed their efficacy and safety,^[50]^ while clinical guidelines have endorsed ozanimod for UC,^[51]^ and highlighted etrasimod’s potential for the treatment of moderate-to-severe UC.^[17]^ Guidance on drug-drug interactions has also been issued for both agents.^[52]^ The 2024 American Gastroenterological Association guidelines recommended ozanimod and etrasimod for adult outpatients naïve to advanced therapies,^[32]^ and a US study positioned them as first-line oral therapies after 5-aminosalicylic acid failure.^[16]^

This study assessed the real-world safety profiles of ozanimod and etrasimod in the UC population using individual case safety reports from the FAERS database. In descriptive analyses, ozanimod-associated reports significantly outnumbered those for etrasimod, reflecting its earlier approval and longer market availability. A slight female predominance was observed for both drugs in our study, consistent with a prior study.^[53]^ A substantial proportion of reports involved elderly patients, supporting earlier findings of a rise in UC incidence in later decades of life.^[3,54]^ Approximately two-thirds of reports were submitted by healthcare professionals, enhancing data reliability, while the remaining third came from consumers, highlighting the importance patients place on drug safety. As reports were predominantly from the United States, future multi-regional studies are warranted to enhance the generalizability of these findings.

This study used disproportionality analysis to detect AE signals associated with the 2 S1PR modulators in the UC population. At the SOC level, positive signals for nervous system, cardiac, and eye disorders were detected for both drugs, suggesting a potential association with drug use. At the PT level, ozanimod yielded substantially more positive signals than etrasimod. A similar pattern was observed for serious cases, which were more frequently reported for ozanimod. This discrepancy should be interpreted in the context of market availability and cumulative exposure. Ozanimod was approved for UC nearly 2 years before etrasimod, resulting in a longer post-marketing period and a substantially higher volume of reports. Normalization was not performed, as FAERS lacks denominator data (e.g., total prescriptions or patient exposure) to calculate incidence rates. Moreover, notoriety bias may also contribute to the observed differences, whereby drugs that have been on the market longer or have received greater attention tend to generate more reports.^[55]^ These differences warrant further validation in studies with longer follow-up and larger patient cohorts. Nevertheless, the consistent identification of key safety signals (e.g., nervous system, eye, and cardiac disorders) and the unexpected signals for ozanimod (tremor, brain fog, and balance disorder) across sensitivity analyses supports the robustness of the findings.

Within nervous system disorders, ozanimod was associated with 11 PTs, including headache, dizziness, disturbance in attention, cerebrovascular accident, seizure, transient ischemic attack, MS, neuralgia, tremor, brain fog, and balance disorder. Among these, headache, dizziness, and transient ischemic attack were more frequently observed in clinical trials.^[24,25,28,56]^ Notably, tremor, brain fog, and balance disorder were not previously described in trials or prescribing information. For etrasimod, only headache and dizziness were identified, which were also captured in relevant trials^[28,30,42]^ and recorded in prescribing information. The mechanisms underlying these AEs remain incompletely understood. S1P_1_ receptors are widely distributed across immune cells, the cardiovascular system, and the central nervous system, whereas S1P_5_ receptors are predominantly found in the central nervous system.^[57]^ Neurological AEs associated with S1PR modulators may be related to the direct modulation of S1P receptors on neurons and glial cells, thereby disrupting synaptic signaling and neuroimmune balance.^[57]^ Using the VigiAccess database, Haldar et al identified neurological AEs related to several S1PR modulators, including headache, dizziness, neuralgia, MS, seizure, and tremor.^[57]^ These safety signals are similar to those identified in our study. Although the precise pathways require further investigation, these findings highlight the importance of clinical vigilance for neurological symptoms, necessitating pretreatment cerebrovascular assessment and strict avoidance of use in patients with a recent stroke or transient ischemic attack.

Bradycardia, atrioventricular block, and hypertension have been documented as cardiovascular AEs in association with S1PR modulators.^[13]^ As reported in randomized controlled trials for UC,^[14,15,24,25,28,30]^ bradycardia and atrioventricular blocks represent a dose-dependent, transient risk of S1PR modulators,^[58]^ most of which were mild and resolved without intervention.^[13]^ In our study, we identified 4 signals (palpitations, cardiac flutter, arrhythmia, extrasystoles) for ozanimod and 2 signals (bradycardia, palpitations) for etrasimod as cardiac AEs, consistent with previous findings.^[59]^ In cardiomyocytes, activation of S1P_1_ receptors triggers G-protein-coupled inwardly rectifying potassium channels, which play a key role in regulating cardiac conduction and heart rate.^[60]^ This leads to a transient reduction in cardiomyocyte excitability and membrane hyperpolarization,^[60]^ which may represent the underlying pathway for these observed cardiac AEs. Compared with fingolimod, a nonselective S1P_1,3,4,5_ receptor modulator, newer S1PR modulators such as ozanimod show a better cardiac safety profile due to increased receptor selectivity.^[59]^ S1PR modulators may be related to new-onset hypertension.^[58]^ In clinical trials, hypertension has been reported as a potential AE associated with S1PR modulators in UC.^[14,15,25,27]^ Consistent with previous studies, we detected hypertension as a vascular AE for both drugs. Although not fully elucidated, this effect is thought to be mediated by S1P receptor modulation in vascular smooth muscle cells and T-cell mobilization, ultimately leading to vasoconstriction and increased peripheral resistance.^[58]^ Collectively, these findings support adherence to prescribing recommendations, including baseline electrocardiogram screening and periodic monitoring for cardiac effects, along with regular blood pressure monitoring, particularly in patients with pre‑existing cardiovascular risk factors.

Ocular events are commonly observed with S1PR modulator use,^[18,61]^ with macular edema remaining an AE of special interest in several clinical trials for UC.^[14,15,27]^ Clinical trials reported 6 cases with ozanimod^[15,25,27]^ and 2 cases (0.38%) with etrasimod,^[14]^ all of which resolved after drug discontinuation. In our study, 8 ocular signals were detected for ozanimod, including macular edema, blurred vision, visual impairment, photophobia, eye pain, eye disorder, increased lacrimation, and vitreous floaters. For etrasimod, 4 ocular signals, including blurred vision, visual impairment, macular edema, and eye pain, were identified. Since S1P_1_ plays a critical role in maintaining vascular permeability, S1PR modulators may increase vascular permeability as functional antagonists of S1P_1_, thereby precipitating macular edema. As part of the ocular signals detected in our study, blurred vision and visual impairment are common clinical manifestations of macular edema. These findings support clinical vigilance for ophthalmic events, with baseline eye examination recommended for patients initiating therapy and contraindication in patients with prior macular edema. In addition, other ophthalmic events identified in our study may arise from distinct mechanisms and warrant further investigation in future research.

The sensitivity analysis using the entire FAERS database revealed discrepancies in AE signals compared with the UC-specific primary analysis. These differences underscored the presence of indication bias and validate our choice of a disease-specific comparator, which isolates drug effects from UC-related confounders. Notably, tremor, brain fog and balance disorder for ozanimod persisted across both analyses, supporting their robustness as potential drug-related AEs. When the analysis was restricted to reports from healthcare professionals, or when concomitant medications were excluded, the 3 SOC level signals were reproduced for both drugs, and the majority of PT level signals remained consistent with the primary analysis, further supporting the robustness of our findings.

The TTO analysis showed that most AEs associated with ozanimod and etrasimod occurred within the first month of treatment, with no significant difference in cumulative incidence between the 2 drugs. Weibull distribution analysis indicated an early failure pattern for both drugs, suggesting that the risk of AEs declined over time and supporting the need for intensified monitoring during the initial phase of therapy.

Our study has the following limitations. First, the FAERS database is a spontaneous reporting system, which is inherently subject to underreporting, misreporting, and incomplete data. These factors may introduce reporting bias. Second, most reports originated from the United States, which may limit the generalizability of our findings to other regions due to differences in epidemiology, treatment practices, and drug responses. Third, residual confounding cannot be entirely ruled out. Factors such as prior medication history, drug dosage, comorbidities, and concomitant medications may have influenced the results, although we attempted to mitigate this by excluding the top 30 concomitant medications and a range of immunosuppressants in a sensitivity analysis. Fourth, the moderate overlap in PT level signals between the primary UC-specific analysis and the full-database sensitivity analysis reflects the presence of indication bias and underscores the importance of using disease-specific comparators. While the UC-background design controls for this bias, the sensitivity analysis provides complementary context for interpreting the findings. Finally, disproportionality analysis identifies statistical associations but does not establish causality. Despite these limitations, the FAERS database remains a valuable resource for post-marketing safety surveillance.

## 5. Conclusions

In this study, we comprehensively analyzed AEs associated with ozanimod and etrasimod in the UC population using the FAERS database. Our findings were largely consistent with the safety profiles from clinical trials and prescribing information. Of note, we also identified potential AE signals for ozanimod not currently mentioned in its label, including tremor, brain fog, and balance disorder. The majority of AEs occurred within the first month of therapy, underscoring the need for early monitoring. This real‑world study provides safety references to support individualized clinical decision‑making. Despite these insights, further research, including prospective studies, is warranted to confirm these signals and explore the underlying mechanisms.

## Acknowledgments

The authors would like to thank all reviewers for their valuable comments.

## Author contributions

**Conceptualization:** Yongchao Lu, Long Chen.

**Supervision:** Yongchao Lu.

**Data curation:** Long Chen, Dongping Wan.

**Formal analysis:** Long Chen, Benkai Xing, Xiaohui Ji.

**Methodology:** Long Chen, Dongping Wan.

**Software:** Long Chen.

**Visualization:** Benkai Xing.

**Resources:** Xiaohui Ji.

**Investigation:** Wenyan Zhou, Yanbing Liu.

**Writing – review & editing:** Yongchao Lu, Dongping Wan.

**Writing – original draft:** Long Chen.




























