# Supplementary material for: Safety of ozanimod and etrasimod in ulcerative colitis: A disproportionality analysis of the Food and Drug Administration Adverse Event Reporting System database
Source: Medicine (Baltimore). 2026 Aug 21;105(34):e50262. doi: 10.1097/MD.0000000000050262 (PMC13502775; doi:10.1097/MD.0000000000050262)
Supplement: Supplementary file 1 [file medi-105-e50262-s001.docx]

**Title:**

**Safety of ozanimod and etrasimod in ulcerative colitis: a disproportionality analysis of the Food and Drug Administration Adverse Event Reporting System database**

**Journal name:**

Medicine

**Author names:**

Long Chen, Dongping Wan, Benkai Xing, Xiaohui Ji, Wenyan Zhou, Yanbing Liu, Yongchao Lu^*^.

***Corresponding authors:**

Yongchao Lu

Department of Traditional Chinese Medicine(Proctology Division), Shandong Provincial Hospital Affiliated to Shandong First Medical University, Jinan 250021, Shandong, China.

**E-mail address**:luyongchaojn@163.com

**Supplementary Table S1. The REporting of A Disproportionality analysis for drUg Safety signal detection using individual case safety reports in PharmacoVigilance (READUS-PV) checklist.**

| **Section and topic** | **Item** | **Checklist item** | **Reported on Page/Lines** |
| --- | --- | --- | --- |
| **Title** |  |  |  |
|  | 1a | If disproportionality analyses are a prominent component of the published study, the study should be identified as a “disproportionality analysis”. The type of data and name of the database(s) should be specified. | Page 1, Lines 1-3 |
|  | 1b | Report the name of adverse event(s) and/or drug(s) under study, when applicable. | Page 1, Lines 1-3 |
| **Introduction** |  |  |  |
| **Background** | 2a | Describe the drug(s) and its utilization, the nature of the adverse event(s) under study and its frequency, and the existing knowledge on the drug-event combination. | Pages 4-5, Lines 91-121 |
|  | 2b | Specify the rationale for performing the analysis, e.g., as part of routine pharmacovigilance, to investigate an overall safety profile, or to assess a pre-specified hypothesis. | Page 5, Lines 111-115 |
|  | 2c | Explain why ICSR databases and disproportionality analysis are suitable to fill the knowledge gap. | Page 5, Lines 115-121 |
| **Objectives** | 3 | State specific objectives, identifying the adverse event(s), the drug(s), and the reference group, including any pre-specified hypothesis, if applicable. | Page 5, Lines 122-126 |
| **Methods** |  |  |  |
| **Study design** | 4a | Identify the study (i.e., “disproportionality analysis”) and the type of data used (e.g., “individual case safety reports”). | Pages 6-7 , Lines 130-175 |
|  | 4b | Provide an outline of the entire study design, including primary and sensitivity analyses performed, and other designs such as case-by-case analysis or literature review. | Pages 6-8, Lines 129-202 |
| **Data description, access,and pre-processing** | 5a | Specify the name of the database(s), the database(s) custodian, and the coverage. Specify the type/number of drugs included within the database and the thesaurus, taxonomies, or ontologies used for coding drugs and events. | Page 5, Lines 115-121 Page 6, Lines 132-140 |
|  | 5b | Specify the extraction dates and describe and justify all choices used for data pre-processing, including any data transformation or exclusion, if appropriate. | Page 6-7, Lines 130-155 |
| **Variables definition** | 6a | Describe the study population, including any restriction. | Page 6, Lines 144-151 |
|  | 6b | Describe the nature and the meaning of key variables assessed in the work. | Page 6, Lines 144-151 |
|  | 6c | Specify and justify any grouping of drugs or events. For drugs, specify and justify whether active ingredients/trade names/salts were considered and/or the selected role. | Page 6, Lines 130-132 Page 9, Line 209 |
|  | 6d | Describe any additional data source used, the type of data, and how they interact with ICSRs. | Not applicable |
| **Statistical methods** | 7a | Present any descriptive analysis performed, specifying variables investigated, statistical tests, and significance thresholds. | Page 7, Lines 165-175 |
|  | 7b | Describe the measure(s) selected for the disproportionality analysis including any threshold used to identify signals of disproportionate reporting. Explain the reason for this choice if applicable. | Page 7, Lines 157-175 |
|  | 7c | Clearly describe any sensitivity analysis and any tool to control confounding, including any restriction, subgroup, stratification, adjustment, or interaction. | Pages 7-8, Lines 157-188 |
|  | 7d | Specify the variables and methods used for the case-by-case analysis, including any algorithm or criteria used to assess causality, if performed. | Page 8, Lines 197-202 |
|  | 7e | Specify any statistical methods used for other data sources. | Not applicable |
| **Results** |  |  |  |
| **Participants** | 8a | Specify the number of individual case safety reports included at each stage, including reasons for exclusion. | Page 8, Lines 206-217 |
|  | 8b | Provide key demographic and clinical characteristics of cases, if possible comparing cases with any appropriate reference group. | Page 8, Lines 206-217 |
| **Disproportionality analysis** | 9 | Present all results including confidence intervals. Present also results of sensitivity analyses, if performed. | Page 9-11, Lines 220-264 |
| **Case-by-case analysis** | 10 | Present the case-by-case analysis of key variables. Present the causality assessment, if applicable. | Page 11-12, Lines 276-284 |
| **Discussion** |  |  |  |
| **Key results** | 11 | Discuss key results with reference to study objectives and contextualize them within the current literature and other consulted sources. Clearly discriminate between expected reactions and emerging safety signals. | Page 13-16, Lines 311-389 |
| **External validity** | 12a | Discuss the external validity of the results to the general population. | Page 16, Lines 390 |
|  | 12b | Discuss the potential relevance of results in clinical practice | Page 14-16, Lines 337-389 |
|  | 12c | Propose further study designs if applicable | Page 14, Lines 351-354 |
| **Limitations** | 13 | Present general limitations, making clear that disproportionality analysis alone cannot prove causation or measure incidence, and specific limitations, including confounding and reporting bias and efforts to mitigate them. | Page 16-17, Lines 404-418 |
| **Declarations** |  |  |  |
|  | 14a | Provide the source of funding/sponsorship and the role of the funders/sponsors for the present study and for any original study on which the present article is based. | Page 18, Lines 442 |
|  | 14b | Clearly identify potential commercial and intellectual conflicts of interest (e.g., link to any drug/event investigated, whether financial, legal action, or software used). | Page 18, Lines 439 |
|  | 14c | Declare any institutional approval needed or granted in the investigation. | Not applicable |
|  | 14d | Include a statement on data availability, code availability (including the version of the statistical software used), and protocol registration. | Page 18, Lines 445-446 |
