# Supplementary material for: Safety of ozanimod and etrasimod in ulcerative colitis: A disproportionality analysis of the Food and Drug Administration Adverse Event Reporting System database
Source: Medicine (Baltimore). 2026 Aug 21;105(34):e50262. doi: 10.1097/MD.0000000000050262 (PMC13502775; doi:10.1097/MD.0000000000050262)
Supplement: Supplementary file 2 [file medi-105-e50262-s002.docx]

**Supplementary Table S3. The top 30 concomitant medications of the adverse event reports of ozanimod and etrasimod.**

| **No** | **Co-administration drugs of ozanimod** | **n** | **Co-administration drugs of etrasimod** | **n** |
| --- | --- | --- | --- | --- |
| 1 | prednisone | 80 | mesalamine | 24 |
| 2 | mesalamine | 41 | prednisone | 12 |
| 3 | budesonide | 23 | budesonide | 9 |
| 4 | vitamin d3 | 18 | sulfasalazine | 6 |
| 5 | omeprazole | 16 | levothyroxine | 5 |
| 6 | pantoprazole | 15 | herbals turmeric | 3 |
| 7 | vitamins | 15 | pantoprazole | 3 |
| 8 | humira (adalimumab) | 13 | adderall | 2 |
| 9 | vitamins nos | 13 | amlodipine | 2 |
| 10 | atorvastatin | 12 | aspirin | 2 |
| 11 | lisinopril | 12 | atorvastatin | 2 |
| 12 | xeljanz(tofacitinib) | 12 | bisoprolol | 2 |
| 13 | iron | 11 | dicyclomine | 2 |
| 14 | lialda | 11 | furosemide | 2 |
| 15 | amlodipine | 10 | iron | 2 |
| 16 | aspirin | 10 | lialda | 2 |
| 17 | hydrochlorothiazide | 10 | lidocaine | 2 |
| 18 | tylenol | 10 | lipitor | 2 |
| 19 | apriso | 9 | methimazole | 2 |
| 20 | metoprolol | 9 | norco | 2 |
| 21 | stelara(ustekinumab) | 9 | omeprazole | 2 |
| 22 | vitamin b12 | 9 | pentasa | 2 |
| 23 | ergocalciferol | 8 | prozac | 2 |
| 24 | gabapentin | 8 | trazodone | 2 |
| 25 | jardiance | 8 | vitamin d3 | 2 |
| 26 | lexapro | 8 | acetaminophen and codeine | 1 |
| 27 | losartan | 8 | acetaminophen hydrocodone | 1 |
| 28 | metformin | 8 | adalimumab | 1 |
| 29 | rinvoq (upadacitinib) | 8 | albuterol sulfate | 1 |
| 30 | rosuvastatin | 8 | amitiza | 1 |
