# Supplementary material for: Safety of ozanimod and etrasimod in ulcerative colitis: A disproportionality analysis of the Food and Drug Administration Adverse Event Reporting System database
Source: Medicine (Baltimore). 2026 Aug 21;105(34):e50262. doi: 10.1097/MD.0000000000050262 (PMC13502775; doi:10.1097/MD.0000000000050262)
Supplement: Supplementary file 3 [file medi-105-e50262-s003.docx]

**Supplementary Table S4. Additional key immunosuppressive medications commonly used in UC that not included in the top 30 concomitant medications list**

| **Drug Class** | **Drug Name** |
| --- | --- |
| Thiopurines | azathioprine |
|  | mercaptopurine |
| Antimetabolites | methotrexate |
| Calcineurin inhibitors | ciclosporin (cyclosporine) |
|  | tacrolimus |
| Biologics (anti-TNF) | infliximab |
|  | golimumab |
|  | certolizumab pegol |
| Biologics (anti-integrin) | vedolizumab |
| JAK inhibitors | filgotinib |
