# Supplementary material for: Safety of ozanimod and etrasimod in ulcerative colitis: A disproportionality analysis of the Food and Drug Administration Adverse Event Reporting System database
Source: Medicine (Baltimore). 2026 Aug 21;105(34):e50262. doi: 10.1097/MD.0000000000050262 (PMC13502775; doi:10.1097/MD.0000000000050262)
Supplement: Supplementary file 4 [file medi-105-e50262-s004.docx]

**Supplementary Table S5. Retrieved drug information of ozanimod and etrasimod in the FAERS database.**

| **Drug name** | **Active ingredient** | **Number of reports** |
| --- | --- | --- |
| zeposia | ozanimod hydrochloride | 1617 |
| ozanimod | ozanimod | 15 |
| velsipity | etrasimod arginine | 276 |
| etrasimod | etrasimod | 7 |
