# Supplementary material for: Safety of ozanimod and etrasimod in ulcerative colitis: A disproportionality analysis of the Food and Drug Administration Adverse Event Reporting System database
Source: Medicine (Baltimore). 2026 Aug 21;105(34):e50262. doi: 10.1097/MD.0000000000050262 (PMC13502775; doi:10.1097/MD.0000000000050262)
Supplement: Supplementary file 5 [file medi-105-e50262-s005.docx]

**Supplementary Table S6. Positive adverse event signals of ozanimod and etrasimod at the PT level in UC population.**

| **Ozanimod** | | | | | |
| --- | --- | --- | --- | --- | --- |
| **PT** | **n** | **ROR(95%Cl)** | **PRR(95%CI)** | **χ²** | **IC(IC025)** |
| Drug ineffective | 201 | 2.35(2.03,2.71) | 2.27(2.14,2.41) | 143.46 | 1.17(0.96) |
| Fatigue | 125 | 2.38(1.99,2.85) | 2.33(2.16,2.51) | 94.54 | 1.20(0.94) |
| Headache | 91 | 2.49(2.02,3.08) | 2.46(2.25,2.66) | 77.43 | 1.28(0.97) |
| Haemorrhage | 66 | 5.83(4.54,7.49) | 5.75(5.50,5.99) | 245.22 | 2.46(2.09) |
| Nausea | 64 | 2.03(1.58,2.61) | 2.01(1.77,2.26) | 32.21 | 0.99(0.63) |
| Dizziness | 60 | 3.02(2.33,3.92) | 2.99(2.74,3.25) | 77.61 | 1.55(1.17) |
| Back pain | 45 | 3.65(2.71,4.93) | 3.62(3.33,3.92) | 82.64 | 1.82(1.38) |
| Dyspnoea | 42 | 2.39(1.76,3.26) | 2.38(2.07,2.68) | 32.88 | 1.23(0.78) |
| Abdominal pain upper | 36 | 2.26(1.62,3.15) | 2.25(1.92,2.58) | 24.50 | 1.15(0.67) |
| Vision blurred | 31 | 9.26(6.40,13.41) | 9.19(8.83,9.56) | 207.15 | 3.09(2.55) |
| Hypertension | 29 | 2.54(1.76,3.68) | 2.53(2.16,2.90) | 26.23 | 1.32(0.78) |
| Palpitations | 25 | 5.13(3.42,7.67) | 5.10(4.70,5.50) | 78.37 | 2.29(1.71) |
| Peripheral swelling | 23 | 2.55(1.68,3.86) | 2.54(2.13,2.95) | 20.94 | 1.32(0.72) |
| Muscle spasms | 23 | 2.58(1.70,3.91) | 2.57(2.16,2.98) | 21.54 | 1.34(0.74) |
| Visual impairment | 23 | 7.05(4.61,10.78) | 7.01(6.59,7.43) | 110.74 | 2.72(2.11) |
| Flatulence | 20 | 2.00(1.28,3.12) | 2.00(1.56,2.44) | 9.79 | 0.98(0.35) |
| Chest pain | 18 | 2.10(1.32,3.35) | 2.09(1.63,2.56) | 10.11 | 1.05(0.38) |
| Disturbance in attention | 18 | 23.92(14.27,40.07) | 23.81(23.29,24.32) | 316.40 | 4.27(3.55) |
| White blood cell count decreased | 17 | 12.29(7.41,20.36) | 12.23(11.73,12.74) | 155.94 | 3.46(2.74) |
| Maternal exposure during pregnancy | 17 | 2.01(1.24,3.25) | 2.00(1.53,2.48) | 8.41 | 0.99(0.30) |
| Feeling abnormal | 16 | 2.38(1.45-3.91) | 2.37(1.88,2.87) | 12.43 | 1.23(0.52) |
| Hepatic enzyme increased | 16 | 2.67(1.62,4.38) | 2.66(2.16,3.15) | 16.14 | 1.39(0.68) |
| Myalgia | 15 | 2.01(1.20,3.35) | 2.00(1.49,2.51) | 7.40 | 0.99(0.26) |
| Lymphocyte count decreased | 15 | 43.35(23.59,79.65) | 43.18(42.57,43.78) | 428.88 | 4.92(4.09) |
| Pollakiuria | 14 | 13.48(7.70,23.59) | 13.43(12.88,13.99) | 141.71 | 3.58(2.78) |
| Cerebrovascular accident | 14 | 3.15(1.85,5.37) | 3.14(2.61,3.67) | 19.84 | 1.62(0.86) |
| Confusional state | 13 | 2.46(1.42,4.26) | 2.45(1.90,3.00) | 10.91 | 1.27(0.49) |
| Macular oedema | 13 | 116.07(51.96,259.25) | 115.66(114.86,116.47) | 677.35 | 5.74(4.78) |
| Bowel movement irregularity | 12 | 4.24(2.37,7.55) | 4.22(3.65,4.80) | 28.34 | 2.03(1.21) |
| Influenza like illness | 11 | 2.50(1.37,4.56) | 2.50(1.90,3.10) | 9.65 | 1.30(0.45) |
| Therapy interrupted | 10 | 4.76(2.52,8.99) | 4.75(4.12,5.38) | 28.26 | 2.19(1.30) |
| Neck pain | 9 | 4.60(2.35,8.98) | 4.59(3.92,5.26) | 24.13 | 2.15(1.21) |
| Oedema peripheral | 9 | 2.99(1.54,5.81) | 2.99(2.32,3.65) | 11.54 | 1.55(0.62) |
| Tremor | 8 | 2.37(1.17,4.78) | 2.37(1.66,3.07) | 6.16 | 1.22(0.25) |
| Seizure | 8 | 3.12(1.54,6.32) | 3.12(2.42,3.82) | 11.17 | 1.61(0.63) |
| Transient ischaemic attack | 8 | 8.35(4.05,17.19) | 8.33(7.61,9.05) | 47.56 | 2.96(1.95) |
| Muscular weakness | 7 | 3.09(1.46,6.56) | 3.09(2.33,3.84) | 9.57 | 1.60(0.56) |
| Dysuria | 7 | 5.81(2.71,12.47) | 5.81(5.04,6.57) | 26.30 | 2.47(1.42) |
| Cardiac flutter | 7 | 34.32(14.5,81.21) | 34.25(33.39,35.11) | 167.41 | 4.68(3.52) |
| Arrhythmia | 7 | 7.22(3.35,15.57) | 7.21(6.44,7.98) | 34.89 | 2.76(1.70) |
| Brain fog | 7 | 7.07(3.28,15.24) | 7.06(6.30,7.83) | 33.99 | 2.73(1.68) |
| Photophobia | 6 | 9.19(3.98,21.23) | 9.18(8.34,10.01) | 39.97 | 3.08(1.94) |
| Epistaxis | 6 | 3.87(1.71,8.75) | 3.86(3.05,4.68) | 12.25 | 1.91(0.79) |
| Faeces discoloured | 6 | 2.67(1.19,6.02) | 2.67(1.86,3.48) | 6.10 | 1.39(0.28) |
| Leukopenia | 6 | 8.52(3.70,19.64) | 8.51(7.68,9.34) | 36.59 | 2.98(1.84) |
| Balance disorder | 5 | 2.66(1.09,6.47) | 2.66(1.77,3.55) | 5.04 | 1.39(0.19) |
| Micturition urgency | 5 | 6.28(2.54,15.52) | 6.27(5.37,7.18) | 20.83 | 2.57(1.35) |
| Abdominal tenderness | 5 | 7.90(3.18,19.67) | 7.89(6.98,8.80) | 27.86 | 2.88(1.65) |
| Neutropenia | 5 | 7.31(2.95,18.15) | 7.30(6.40,8.21) | 25.32 | 2.78(1.55) |
| Covid-19 pneumonia | 4 | 3.59(1.32,9.75) | 3.59(2.59,4.59) | 7.22 | 1.81(0.49) |
| Respiratory tract congestion | 4 | 3.56(1.31,9.66) | 3.56(2.56,4.56) | 7.10 | 1.79(0.48) |
| Eye pain | 4 | 3.50(1.29,9.49) | 3.50(2.50,4.49) | 6.88 | 1.77(0.45) |
| Skin exfoliation | 4 | 4.12(1.52,11.22) | 4.12(3.12,5.12) | 9.07 | 2.00(0.68) |
| Liver function test increased | 4 | 4.35(1.6,11.86) | 4.35(3.35,5.35) | 9.88 | 2.07(0.75) |
| Eye disorder | 4 | 4.45(1.63,12.13) | 4.45(3.45,5.45) | 10.23 | 2.10(0.78) |
| Multiple sclerosis | 4 | 6.22(2.26,17.10) | 6.21(5.20,7.22) | 16.46 | 2.56(1.22) |
| Urinary incontinence | 4 | 6.76(2.45,18.62) | 6.75(5.74,7.76) | 18.33 | 2.67(1.33) |
| Infrequent bowel movements | 4 | 5.60(2.04,15.34) | 5.59(4.59,6.60) | 14.27 | 2.42(1.08) |
| Lymphopenia | 4 | 11.20(3.98,31.52) | 11.19(10.15,12.22) | 33.30 | 3.34(1.97) |
| Increased appetite | 3 | 7.17(2.22,23.15) | 7.16(5.99,8.33) | 14.82 | 2.75(1.25) |
| Neuralgia | 3 | 3.87(1.22,12.26) | 3.86(2.71,5.02) | 6.13 | 1.91(0.43) |
| Pelvic pain | 3 | 5.54(1.73,17.75) | 5.54(4.38,6.70) | 10.56 | 2.41(0.91) |
| Mental status changes | 3 | 48.97(12.24,195.9) | 48.93(47.55,50.32) | 93.91 | 5.04(3.33) |
| Extrasystoles | 3 | 26.71(7.45,95.79) | 26.69(25.42,27.97) | 58.29 | 4.41(2.78) |
| Quality of life decreased | 3 | 5.34(1.67,17.08) | 5.34(4.18,6.50) | 10.03 | 2.35(0.86) |
| Hyperglycaemia | 3 | 29.38(8.08,106.81) | 29.36(28.07,30.65) | 63.22 | 4.51(2.88) |
| Gastrointestinal motility disorder | 3 | 4.45(1.40,14.16) | 4.45(3.29,5.61) | 7.67 | 2.10(0.62) |
| Renal cancer | 3 | 8.39(2.58,27.31) | 8.39(7.21,9.57) | 17.98 | 2.96(1.45) |
| Chromaturia | 3 | 4.45(1.40,14.16) | 4.45(3.29,5.61) | 7.67 | 2.10(0.62) |
| Lacrimation increased | 3 | 4.14(1.30,13.14) | 4.14(2.98,5.29) | 6.84 | 2.00(0.52) |
| Middle insomnia | 3 | 4.14(1.30,13.14) | 4.14(2.98,5.29) | 6.84 | 2.00(0.52) |
| Mood altered | 3 | 8.90(2.73,29.04) | 8.90(7.72,10.08) | 19.28 | 3.04(1.52) |
| Norovirus infection | 3 | 8.39(2.58,27.31) | 8.39(7.21,9.57) | 17.98 | 2.96(1.45) |
| Urine abnormality | 3 | 24.49(6.91,86.81) | 24.47(23.20,25.73) | 54.02 | 4.31(2.69) |
| Vitreous floaters | 3 | 8.39(2.58,27.31) | 8.39(7.21,9.57) | 17.98 | 2.96(1.45) |
| **Etrasimod** | | | | | |
| **PT** | **n** | **ROR(95%Cl)** | **PRR(95%CI)** | **χ²** | **IC(IC025)** |
| Drug ineffective | 57 | 3.85(2.93,5.05) | 3.62(3.37,3.87) | 109.44 | 1.85(1.45) |
| Headache | 25 | 3.42(2.29,5.11) | 3.34(2.95,3.73) | 41.01 | 1.73(1.15) |
| Dizziness | 24 | 6.46(4.28,9.74) | 6.28(5.88,6.68) | 105.08 | 2.63(2.04) |
| Nausea | 17 | 2.84(1.75,4.60) | 2.79(2.32,3.27) | 19.57 | 1.47(0.78) |
| Vision blurred | 13 | 20.16(11.46,35.46) | 19.82(19.26,20.37) | 218.93 | 4.23(3.42) |
| Pain | 12 | 2.13(1.20,3.77) | 2.11(1.54,2.67) | 6.99 | 1.07(0.26) |
| Therapeutic product effect incomplete | 11 | 5.01(2.75,9.13) | 4.95(4.36,5.54) | 34.27 | 2.29(1.44) |
| Bradycardia | 8 | 36.45(17.49,75.99) | 36.07(35.34,36.79) | 245.22 | 5.02(4.00) |
| Hypertension | 6 | 2.46(1.10,5.51) | 2.45(1.65,3.25) | 5.11 | 1.28(0.18) |
| Visual impairment | 5 | 8.26(3.39,20.12) | 8.21(7.32,9.09) | 30.88 | 3.00(1.80) |
| Hypotension | 5 | 6.29(2.59,15.28) | 6.25(5.37,7.13) | 21.66 | 2.62(1.42) |
| Lymphocyte count decreased | 5 | 61.97(23.73,161.84) | 61.56(60.6,62.51) | 249.86 | 5.69(4.41) |
| Influenza like illness | 5 | 5.23(2.15,12.68) | 5.20(4.32,6.08) | 16.70 | 2.36(1.16) |
| Type 2 diabetes mellitus | 5 | 4.89(2.02,11.87) | 4.86(3.98,5.74) | 15.14 | 2.27(1.07) |
| Gastrooesophageal reflux disease | 4 | 6.6(2.44-17.80) | 6.57(5.58,7.55) | 18.51 | 2.69(1.38) |
| White blood cell count decreased | 4 | 13.27(4.87,36.16) | 13.2(12.2,14.2) | 43.34 | 3.67(2.34) |
| Chills | 4 | 3.04(1.13,8.15) | 3.03(2.04,4.01) | 5.39 | 1.59(0.29) |
| Hepatic enzyme increased | 4 | 2.89(1.08,7.75) | 2.88(1.90,3.86) | 4.87 | 1.52(0.21) |
| Palpitations | 4 | 3.62(1.35,9.73) | 3.61(2.62,4.59) | 7.46 | 1.84(0.54) |
| Loss of therapeutic response | 4 | 2.73(1.02,7.33) | 2.72(1.74,3.71) | 4.34 | 1.44(0.14) |
| Blood iron decreased | 3 | 5.54(1.76,17.38) | 5.52(4.38,6.66) | 10.92 | 2.44(0.98) |
| Alanine aminotransferase increased | 3 | 17.21(5.38,55.12) | 17.15(15.99,18.31) | 43.31 | 4.03(2.54) |
| Macular oedema | 3 | 56.72(16.59,193.95) | 56.49(55.26,57.71) | 139.00 | 5.59(4.01) |
| Eye pain | 3 | 13.97(4.39,44.49) | 13.92(12.76,15.07) | 34.48 | 3.74(2.26) |
