# Supplementary material for: Safety of ozanimod and etrasimod in ulcerative colitis: A disproportionality analysis of the Food and Drug Administration Adverse Event Reporting System database
Source: Medicine (Baltimore). 2026 Aug 21;105(34):e50262. doi: 10.1097/MD.0000000000050262 (PMC13502775; doi:10.1097/MD.0000000000050262)
Supplement: Supplementary file 6 [file medi-105-e50262-s006.docx]

**Supplementary Table S7. Positive adverse event signals at the SOC level for ozanimod and etrasimod using the full FAERS database.**

| **Ozanimod** | | | | | |
| --- | --- | --- | --- | --- | --- |
| **SOC** | **n** | **ROR(95%Cl)** | **PRR(95%Cl)** | **χ²** | **IC(IC025)** |
| General disorders and administration site conditions | 2893 | 1.11(1.07,1.16) | 1.09(1.06,1.12) | 26.09 | 0.12(0.07) |
| **Nervous system disorders** * | 2758 | 2.95(2.83,3.07) | 2.59(2.56,2.62) | 2889.92 | 1.37(1.31) |
| Gastrointestinal disorders | 1766 | 1.57(1.50,1.65) | 1.50(1.46,1.55) | 323.27 | 0.59(0.52) |
| Investigations | 1032 | 1.21(1.13,1.28) | 1.19(1.13,1.25) | 33.62 | 0.25(0.16) |
| Musculoskeletal and connective tissue disorders | 1000 | 1.33(1.25,1.42) | 1.31(1.25,1.37) | 76.21 | 0.39(0.29) |
| Injury, poisoning and procedural complications | 977 | 0.46(0.43,0.49) | 0.50(0.44,0.56) | 568.59 | -1.01(-1.0) |
| Infections and infestations | 822 | 0.95(0.88,1.02) | 0.95(0.88,1.02) | 2.35 | -0.07(-0.18) |
| Psychiatric disorders | 625 | 0.78(0.72,0.85) | 0.79(0.71,0.87) | 36.75 | -0.34(-0.46) |
| Respiratory, thoracic and mediastinal disorders | 489 | 0.72(0.66,0.78) | 0.73(0.64,0.81) | 52.83 | -0.46(-0.59) |
| Skin and subcutaneous tissue disorders | 483 | 0.60(0.55,0.66) | 0.61(0.53,0.70) | 124.64 | -0.71(-0.84) |
| Eye disorders | 426 | 1.45(1.32,1.60) | 1.44(1.34,1.53) | 57.44 | 0.52(0.38) |
| Vascular disorders | 343 | 1.26(1.14,1.41) | 1.26(1.15,1.36) | 18.59 | 0.33(0.17) |
| Cardiac disorders | 231 | 0.81(0.71,0.92) | 0.81(0.69,0.94) | 10.04 | -0.3(-0.49) |
| Renal and urinary disorders | 190 | 0.74(0.64,0.85) | 0.74(0.60,0.88) | 17.89 | -0.44(-0.65) |
| Blood and lymphatic system disorders | 161 | 0.61(0.53,0.72) | 0.62(0.47,0.77) | 38.45 | -0.69(-0.92) |
| Metabolism and nutrition disorders | 150 | 0.52(0.44,0.61) | 0.53(0.37,0.69) | 64.80 | -0.92(-1.16) |
| Neoplasms benign, malignant and unspecified (incl cysts and polyps) | 135 | 0.22(0.18,0.26) | 0.22(0.06,0.39) | 376.36 | -2.15(-2.4) |
| Immune system disorders | 114 | 0.68(0.56,0.81) | 0.68(0.50,0.86) | 17.53 | -0.56(-0.83) |
| Surgical and medical procedures | 110 | 0.47(0.39,0.57) | 0.47(0.29,0.66) | 65.25 | -1.08(-1.35) |
| Reproductive system and breast disorders | 69 | 0.79(0.62,1.00) | 0.79(0.56,1.03) | 3.80 | -0.34(-0.68) |
| Ear and labyrinth disorders | 64 | 1.07(0.84,1.37) | 1.07(0.82,1.31) | 0.28 | 0.1(-0.26) |
| Social circumstances | 59 | 0.82(0.64,1.06) | 0.82(0.57,1.08) | 2.22 | -0.28(-0.65) |
| Pregnancy, puerperium and perinatal conditions | 48 | 1.01(0.76,1.34) | 1.01(0.72,1.29) | 0.00 | 0.01(-0.4) |
| Hepatobiliary disorders | 45 | 0.36(0.27,0.48) | 0.36(0.07,0.65) | 51.97 | -1.48(-1.9) |
| Product issues | 26 | 0.09(0.06,0.13) | 0.09(-0.30,0.47) | 254.81 | -3.52(-4.08) |
| Endocrine disorders | 13 | 0.32(0.18,0.54) | 0.32(-0.23,0.86) | 19.30 | -1.66(-2.43) |
| Congenital, familial and genetic disorders | 4 | 0.10(0.04,0.27) | 0.10(-0.88,1.08) | 32.12 | -3.31(-4.6) |
| **Etrasimod** | | | | | |
| **SOC** | **n** | **ROR(95%Cl)** | **PRR(95%Cl)** | **χ²** | **IC(IC025)** |
| General disorders and administration site conditions | 493 | 1.65(1.49,1.83) | 1.48(1.41,1.56) | 94.26 | 0.57(0.42) |
| **Gastrointestinal disorders** * | 360 | 2.77(2.47,3.10) | 2.43(2.34,2.52) | 328.53 | 1.28(1.11) |
| Nervous system disorders | 159 | 1.19(1.01,1.40) | 1.17(1.03,1.32) | 4.46 | 0.23(-0.01) |
| Investigations | 145 | 1.36(1.15,1.61) | 1.33(1.18,1.49) | 12.74 | 0.41(0.17) |
| **Eye disorders** * | 139 | 4.00(3.36,4.75) | 3.78(3.62,3.94) | 289.37 | 1.92(1.66) |
| **Metabolism and nutrition disorders** * | 92 | 2.63(2.13,3.24) | 2.55(2.35,2.75) | 88.37 | 1.35(1.04) |
| Infections and infestations | 79 | 0.73(0.58,0.91) | 0.74(0.52,0.95) | 7.80 | -0.44(-0.77) |
| Skin and subcutaneous tissue disorders | 66 | 0.62(0.49,0.80) | 0.64(0.40,0.87) | 14.48 | -0.65(-1.01) |
| Musculoskeletal and connective tissue disorders | 55 | 0.56(0.43,0.73) | 0.57(0.31,0.83) | 18.30 | -0.8(-1.19) |
| Injury, poisoning and procedural complications | 51 | 0.19(0.14,0.25) | 0.21(-0.06,0.48) | 169.93 | -2.23(-2.64) |
| Cardiac disorders | 36 | 0.99(0.71,1.38) | 0.99(0.67,1.31) | 0.00 | -0.01(-0.49) |
| Psychiatric disorders | 36 | 0.35(0.25,0.49) | 0.36(0.04,0.69) | 42.60 | -1.46(-1.94) |
| Respiratory, thoracic and mediastinal disorders | 35 | 0.40(0.29,0.56) | 0.41(0.08,0.74) | 30.87 | -1.28(-1.77) |
| Immune system disorders | 32 | 1.49(1.05,2.11) | 1.48(1.14,1.82) | 5.03 | 0.57(0.06) |
| Vascular disorders | 30 | 0.87(0.60,1.24) | 0.87(0.51,1.22) | 0.61 | -0.2(-0.73) |
| Blood and lymphatic system disorders | 17 | 0.52(0.32,0.84) | 0.53(0.05,1.00) | 7.42 | -0.93(-1.61) |
| Renal and urinary disorders | 13 | 0.38(0.22,0.66) | 0.39(-0.15,0.93) | 12.84 | -1.37(-2.14) |
| Surgical and medical procedures | 9 | 0.31(0.16,0.60) | 0.32(-0.34,0.97) | 13.52 | -1.66(-2.58) |
| Hepatobiliary disorders | 8 | 0.50(0.25,1.01) | 0.51(-0.19,1.20) | 3.89 | -0.98(-1.95) |
| Neoplasms benign, malignant and unspecified (incl cysts and polyps) | 7 | 0.10(0.05,0.20) | 0.10(-0.64,0.84) | 59.15 | -3.33(-4.35) |
| Reproductive system and breast disorders | 6 | 0.52(0.23,1.17) | 0.53(-0.27,1.32) | 2.59 | -0.93(-2.02) |
| Ear and labyrinth disorders | 6 | 0.79(0.35,1.76) | 0.79(-0.01,1.59) | 0.34 | -0.34(-1.43) |
| Endocrine disorders | 5 | 0.98(0.41,2.36) | 0.98(0.11,1.86) | 0.00 | -0.03(-1.21) |
| Social circumstances | 2 | 0.22(0.05,0.87) | 0.22(-1.17,1.60) | 5.62 | -2.2(-3.86) |
| Congenital, familial and genetic disorders | 1 | 0.20(0.03,1.40) | 0.20(-1.76,2.16) | 3.26 | -2.34(-4.38) |

Note: SOCs marked with an asterisk (*) indicate positive signals at the system organ class level for ozanimod and etrasimod ,which met all three detection algorithms.
