# Supplementary material for: Safety of ozanimod and etrasimod in ulcerative colitis: A disproportionality analysis of the Food and Drug Administration Adverse Event Reporting System database
Source: Medicine (Baltimore). 2026 Aug 21;105(34):e50262. doi: 10.1097/MD.0000000000050262 (PMC13502775; doi:10.1097/MD.0000000000050262)
Supplement: Supplementary file 7 [file medi-105-e50262-s007.docx]

**Supplementary Table S9. Adverse event signals of ozanimod and etrasimod at the SOC level submitted by healthcare professionals.**

| **Ozanimod** | | | | | |
| --- | --- | --- | --- | --- | --- |
| **SOC** | **n** | **ROR(95%Cl)** | **PRR(95%Cl)** | **χ²** | **IC(IC025)** |
| General disorders and administration site conditions | 445 | 1.22(1.10,1.35) | 1.17(1.09,1.26) | 13.60 | 0.23(0.08) |
| Gastrointestinal disorders | 443 | 1.31(1.18,1.46) | 1.24(1.16,1.33) | 25.37 | 0.31(0.16) |
| **Nervous system disorders** * | 186 | 2.30(1.98,2.68) | 2.19(2.05,2.32) | 122.22 | 1.11(0.89) |
| Investigations | 134 | 0.71(0.59,0.84) | 0.72(0.56,0.89) | 15.35 | -0.46(-0.72) |
| Musculoskeletal and connective tissue disorders | 127 | 1.12(0.94,1.35) | 1.12(0.95,1.29) | 1.63 | 0.16(-0.11) |
| Infections and infestations | 120 | 0.72(0.60,0.86) | 0.73(0.56,0.91) | 12.60 | -0.45(-0.72) |
| Skin and subcutaneous tissue disorders | 97 | 1.09(0.89,1.33) | 1.08(0.89,1.28) | 0.63 | 0.11(-0.19) |
| **Eye disorders** * | 94 | 5.61(4.54,6.94) | 5.41(5.20,5.61) | 323.71 | 2.38(2.07) |
| Respiratory, thoracic and mediastinal disorders | 81 | 1.13(0.90,1.41) | 1.12(0.91,1.34) | 1.10 | 0.16(-0.16) |
| Vascular disorders | 60 | 1.32(1.02,1.71) | 1.31(1.06,1.56) | 4.51 | 0.39(0.01) |
| Psychiatric disorders | 58 | 1.25(0.96,1.63) | 1.25(0.99,1.50) | 2.84 | 0.31(-0.07) |
| Injury, poisoning and procedural complications | 53 | 0.13(0.10,0.17) | 0.15(-0.11,0.42) | 297.52 | -2.7(-3.1) |
| **Cardiac disorders** * | 50 | 2.48(1.87,3.29) | 2.44(2.17,2.72) | 42.06 | 1.27(0.86) |
| Renal and urinary disorders | 25 | 1.69(1.14,2.52) | 1.68(1.29,2.08) | 6.89 | 0.74(0.17) |
| Blood and lymphatic system disorders | 25 | 1.63(1.10,2.43) | 1.62(1.23,2.02) | 5.96 | 0.69(0.12) |
| Metabolism and nutrition disorders | 22 | 0.74(0.49,1.13) | 0.74(0.33,1.16) | 1.98 | -0.43(-1.03) |
| Immune system disorders | 18 | 1.07(0.67,1.70) | 1.07(0.60,1.53) | 0.08 | 0.09(-0.58) |
| Neoplasms benign, malignant and unspecified (incl cysts and polyps) | 17 | 0.72(0.45,1.17) | 0.72(0.25,1.20) | 1.78 | -0.46(-1.15) |
| Surgical and medical procedures | 13 | 0.60(0.35,1.04) | 0.60(0.06,1.15) | 3.43 | -0.73(-1.5) |
| Social circumstances | 9 | 0.77(0.40,1.48) | 0.77(0.11,1.42) | 0.63 | -0.38(-1.3) |
| Reproductive system and breast disorders | 8 | 1.08(0.54,2.18) | 1.08(0.39,1.78) | 0.05 | 0.11(-0.86) |
| Ear and labyrinth disorders | 7 | 1.07(0.51,2.26) | 1.07(0.33,1.81) | 0.03 | 0.10(-0.93) |
| Hepatobiliary disorders | 6 | 0.40(0.18,0.90) | 0.41(-0.39,1.21) | 5.24 | -1.29(-2.39) |
| Product issues | 4 | 0.53(0.20,1.42) | 0.53(-0.45,1.52) | 1.63 | -0.9(-2.2) |
| Pregnancy, puerperium and perinatal conditions | 3 | 0.31(0.10,0.96) | 0.31(-0.82,1.44) | 4.58 | -1.67(-3.12) |
| **Etrasimod** | | | | | |
| **SOC** | **n** | **ROR(95%Cl)** | **PRR(95%Cl)** | **χ²** | **IC(IC025)** |
| General disorders and administration site conditions | 115 | 1.87(1.50,2.32) | 1.62(1.47,1.78) | 33.04 | 0.69(0.39) |
| Gastrointestinal disorders | 60 | 0.86(0.65,1.13) | 0.88(0.65,1.12) | 1.13 | -0.18(-0.58) |
| **Nervous system disorders** * | 47 | 3.00(2.21,4.07) | 2.77(2.50,3.04) | 54.98 | 1.46(1.02) |
| Investigations | 47 | 1.50(1.10,2.03) | 1.44(1.17,1.71) | 6.83 | 0.52(0.08) |
| **Eye disorders** * | 26 | 8.01(5.36,11.96) | 7.56(7.18,7.93) | 146.08 | 2.89(2.31) |
| Skin and subcutaneous tissue disorders | 21 | 1.19(0.76,1.84) | 1.18(0.76,1.60) | 0.59 | 0.24(-0.4) |
| **Cardiac disorders** * | 17 | 4.53(2.78,7.38) | 4.38(3.91,4.85) | 44.21 | 2.12(1.42) |
| Infections and infestations | 15 | 0.43(0.26,0.72) | 0.45(-0.04,0.95) | 10.76 | -1.14(-1.88) |
| Musculoskeletal and connective tissue disorders | 12 | 0.48(0.27,0.86) | 0.50(-0.06,1.06) | 6.41 | -1(-1.81) |
| Injury, poisoning and procedural complications | 10 | 0.13(0.07,0.24) | 0.15(-0.46,0.76) | 56.67 | -2.72(-3.6) |
| Respiratory, thoracic and mediastinal disorders | 7 | 0.48(0.23,1.01) | 0.49(-0.25,1.22) | 3.91 | -1.04(-2.07) |
| Metabolism and nutrition disorders | 6 | 0.99(0.44,2.21) | 0.99(0.19,1.78) | 0.00 | -0.02(-1.12) |
| Vascular disorders | 6 | 0.76(0.34,1.71) | 0.77(-0.03,1.56) | 0.44 | -0.39(-1.49) |
| Blood and lymphatic system disorders | 6 | 2.06(0.92,4.62) | 2.04(1.25,2.84) | 3.21 | 1.03(-0.08) |
| Psychiatric disorders | 4 | 0.40(0.15,1.07) | 0.41(-0.57,1.38) | 3.55 | -1.3(-2.6) |
| Hepatobiliary disorders | 2 | 0.66(0.16,2.65) | 0.66(-0.72,2.05) | 0.35 | -0.59(-2.27) |
| Endocrine disorders | 1 | 3.33(0.46,23.92) | 3.32(1.36,5.29) | 1.61 | 1.72(-0.34) |
| Renal and urinary disorders | 1 | 0.35(0.05,2.53) | 0.36(-1.60,2.31) | 1.17 | -1.49(-3.53) |
| Ear and labyrinth disorders | 1 | 0.65(0.09,4.63) | 0.65(-1.31,2.61) | 0.19 | -0.62(-2.67) |
| Reproductive system and breast disorders | 1 | 0.62(0.09,4.40) | 0.62(-1.34,2.58) | 0.24 | -0.69(-2.74) |

Note: SOCs marked with an asterisk (*) indicate positive signals at the system organ class level for ozanimod and etrasimod ,which met all three detection algorithms.
