# Supplementary material for: Safety of ozanimod and etrasimod in ulcerative colitis: A disproportionality analysis of the Food and Drug Administration Adverse Event Reporting System database
Source: Medicine (Baltimore). 2026 Aug 21;105(34):e50262. doi: 10.1097/MD.0000000000050262 (PMC13502775; doi:10.1097/MD.0000000000050262)
Supplement: Supplementary file 8 [file medi-105-e50262-s008.docx]

**Supplementary Table S10. Positive adverse event signals of Ozanimod and etrasimod at the PT level submitted by healthcare professionals.**

| **Ozanimod** | | | | | |
| --- | --- | --- | --- | --- | --- |
| **PT** | **n** | **ROR(95%Cl)** | **PRR(95%CI)** | **χ²** | **IC(IC025)** |
| Drug ineffective | 116 | 2.34(1.94,2.83) | 2.27(2.09,2.45) | 82.70 | 1.17(0.89) |
| Fatigue | 77 | 2.85(2.27,3.59) | 2.79(2.56,3.01) | 86.98 | 1.45(1.12) |
| Headache | 50 | 2.47(1.86,3.29) | 2.44(2.16,2.72) | 41.93 | 1.27(0.85) |
| Nausea | 39 | 2.23(1.62,3.07) | 2.21(1.89,2.52) | 25.44 | 1.13(0.66) |
| Dizziness | 33 | 3.08(2.17,4.37) | 3.05(2.71,3.39) | 44.41 | 1.58(1.08) |
| Dyspnoea | 30 | 2.89(2.01,4.17) | 2.86(2.50,3.22) | 35.60 | 1.49(0.96) |
| Back pain | 27 | 4.43(3.01,6.52) | 4.38(4.00,4.77) | 67.88 | 2.09(1.53) |
| Vision blurred | 26 | 16.49(10.89,24.99) | 16.3(15.89,16.71) | 323.43 | 3.83(3.24) |
| Hypertension | 22 | 2.78(1.82,4.26) | 2.76(2.34,3.18) | 24.19 | 1.44(0.83) |
| Asthenia | 21 | 2.35(1.52,3.63) | 2.33(1.90,2.76) | 15.74 | 1.20(0.58) |
| Palpitations | 19 | 6.47(4.06,10.3) | 6.42(5.96,6.88) | 82.00 | 2.61(1.94) |
| Abdominal pain upper | 18 | 2.31(1.44,3.69) | 2.30(1.83,2.76) | 12.93 | 1.18(0.51) |
| Visual impairment | 17 | 12.20(7.37,20.19) | 12.11(11.61,12.61) | 155.42 | 3.45(2.74) |
| Disturbance in attention | 17 | 51.27(28.67,91.66) | 50.86(50.28,51.44) | 559.43 | 5.11(4.32) |
| Chest pain | 16 | 3.07(1.87,5.06) | 3.06(2.56,3.55) | 21.58 | 1.58(0.87) |
| Haemorrhage | 15 | 3.46(2.07,5.80) | 3.44(2.93,3.96) | 25.25 | 1.75(1.02) |
| Peripheral swelling | 14 | 2.81(1.65,4.79) | 2.80(2.27,3.33) | 15.79 | 1.46(0.70) |
| Macular oedema | 13 | 195.67(77.99,490.93) | 194.47(193.55,195.38) | 875.82 | 6.10(5.11) |
| Abdominal distension | 12 | 2.08(1.17,3.69) | 2.08(1.51,2.65) | 6.59 | 1.04(0.23) |
| Myalgia | 12 | 2.80(1.58,4.98) | 2.79(2.22,3.36) | 13.47 | 1.46(0.64) |
| Hepatic enzyme increased | 12 | 2.83(1.59,5.04) | 2.82(2.25,3.40) | 13.79 | 1.47(0.66) |
| Lymphocyte count decreased | 12 | 50.54(25.36,100.74) | 50.26(49.58,50.95) | 391.54 | 5.10(4.17) |
| Feeling abnormal | 10 | 3.44(1.83,6.48) | 3.43(2.8,4.06) | 16.72 | 1.75(0.86) |
| Gastrointestinal disorder | 10 | 2.27(1.21,4.26) | 2.27(1.64,2.89) | 6.94 | 1.16(0.28) |
| White blood cell count decreased | 10 | 15.24(7.84,29.63) | 15.18(14.51,15.84) | 115.70 | 3.74(2.81) |
| Confusional state | 9 | 2.60(1.34,5.04) | 2.59(1.93,3.25) | 8.58 | 1.35(0.42) |
| Paraesthesia | 7 | 2.15(1.02,4.56) | 2.15(1.40,2.90) | 4.22 | 1.09(0.05) |
| Influenza like illness | 7 | 3.10(1.46,6.58) | 3.09(2.34,3.84) | 9.64 | 1.60(0.56) |
| Hypotension | 7 | 2.52(1.19,5.33) | 2.51(1.76,3.26) | 6.22 | 1.31(0.27) |
| Pollakiuria | 7 | 13.62(6.19,29.96) | 13.57(12.79,14.36) | 72.20 | 3.60(2.51) |
| Oedema peripheral | 7 | 3.53(1.66,7.51) | 3.52(2.77,4.28) | 12.25 | 1.78(0.74) |
| Tremor | 6 | 3.16(1.40,7.13) | 3.16(2.35,3.97) | 8.59 | 1.63(0.52) |
| Epistaxis | 6 | 8.87(3.85,20.43) | 8.85(8.02,9.68) | 38.53 | 3.04(1.90) |
| Cerebrovascular accident | 6 | 2.72(1.21,6.13) | 2.72(1.91,3.53) | 6.37 | 1.42(0.31) |
| Hospitalisation | 6 | 2.38(1.06,5.36) | 2.38(1.57,3.19) | 4.70 | 1.23(0.13) |
| Neck pain | 5 | 5.58(2.27,13.73) | 5.57(4.67,6.47) | 17.81 | 2.42(1.20) |
| Seizure | 5 | 4.64(1.89,11.38) | 4.63(3.74,5.53) | 13.65 | 2.16(0.95) |
| Leukopenia | 5 | 8.33(3.35,20.72) | 8.31(7.40,9.22) | 29.80 | 2.96(1.73) |
| Neutropenia | 5 | 9.54(3.81,23.85) | 9.52(8.60,10.43) | 34.95 | 3.14(1.90) |
| Therapy interrupted | 5 | 5.70(2.32,14.04) | 5.69(4.79,6.59) | 18.34 | 2.45(1.23) |
| Covid-19 pneumonia | 4 | 6.36(2.31,17.46) | 6.35(5.34,7.35) | 16.99 | 2.59(1.26) |
| Balance disorder | 4 | 5.67(2.07,15.52) | 5.66(4.65,6.67) | 14.57 | 2.44(1.11) |
| Muscular weakness | 4 | 3.92(1.44,10.65) | 3.91(2.92,4.91) | 8.37 | 1.93(0.61) |
| Photophobia | 4 | 10.23(3.66,28.6) | 10.22(9.19,11.24) | 30.31 | 3.23(1.87) |
| Skin exfoliation | 4 | 8.93(3.21,24.80) | 8.91(7.89,9.93) | 25.90 | 3.05(1.70) |
| Cardiac flutter | 4 | 83.93(22.52,312.77) | 83.77(82.46,85.08) | 181.75 | 5.55(3.97) |
| Rash pruritic | 4 | 2.81(1.04,7.60) | 2.81(1.82,3.80) | 4.55 | 1.47(0.15) |
| Liver function test increased | 4 | 9.76(3.50,27.21) | 9.74(8.72,10.76) | 28.71 | 3.17(1.81) |
| Eye disorder | 4 | 11.65(4.14,32.77) | 11.63(10.60,12.67) | 35.00 | 3.40(2.03) |
| Faeces discoloured | 4 | 4.51(1.66,12.28) | 4.50(3.50,5.50) | 10.46 | 2.12(0.80) |
| Transient ischaemic attack | 4 | 10.23(3.66,28.60) | 10.22(9.19,11.24) | 30.31 | 3.23(1.87) |
| Lymphopenia | 4 | 13.99(4.92,39.74) | 13.96(12.92,15.00) | 42.48 | 3.64(2.26) |
| Brain fog | 4 | 10.76(3.84,30.13) | 10.74(9.71,11.77) | 32.05 | 3.30(1.93) |
| Neuralgia | 3 | 7.49(2.32,24.18) | 7.48(6.31,8.65) | 15.72 | 2.82(1.31) |
| Eye pain | 3 | 5.42(1.70,17.32) | 5.42(4.26,6.58) | 10.27 | 2.38(0.89) |
| Mental status changes | 3 | 104.86(21.15,519.85) | 104.71(103.11,106.31) | 154.09 | 5.72(3.91) |
| Micturition urgency | 3 | 5.42(1.70,17.32) | 5.42(4.26,6.58) | 10.27 | 2.38(0.89) |
| Blood potassium decreased | 3 | 7.67(2.37,24.79) | 7.66(6.49,8.83) | 16.20 | 2.85(1.34) |
| Arrhythmia | 3 | 6.05(1.89,19.38) | 6.04(4.88,7.20) | 11.93 | 2.53(1.03) |
| Urinary incontinence | 3 | 11.23(3.41,36.98) | 11.22(10.03,12.41) | 25.23 | 3.35(1.82) |
| **Etrasimod** | | | | | |
| **PT** | **n** | **ROR(95%Cl)** | **PRR(95%CI)** | **χ²** | **IC(IC025)** |
| Drug ineffective | 33 | 4.07(2.85,5.82) | 3.82(3.49,4.15) | 69.46 | 1.92(1.40) |
| Dizziness | 14 | 7.59(4.43,13.01) | 7.37(6.85,7.89) | 75.83 | 2.86(2.09) |
| Headache | 14 | 3.54(2.07,6.05) | 3.45(2.94,3.97) | 24.41 | 1.78(1.02) |
| Vision blurred | 8 | 26.37(12.78,54.42) | 25.87(25.16,26.58) | 178.45 | 4.60(3.59) |
| Therapeutic product effect incomplete | 7 | 4.92(2.32,10.44) | 4.85(4.11,5.59) | 21.21 | 2.26(1.23) |
| Bradycardia | 6 | 43.31(18.47,101.59) | 42.69(41.84,43.53) | 218.17 | 5.26(4.09) |
| Rash | 6 | 2.39(1.06,5.36) | 2.37(1.57,3.16) | 4.73 | 1.24(0.13) |
| Type 2 diabetes mellitus | 5 | 6.42(2.64,15.63) | 6.35(5.47,7.23) | 22.20 | 2.65(1.45) |
| Lymphocyte count decreased | 4 | 68.42(23.38,200.22) | 67.75(66.69,68.82) | 221.02 | 5.83(4.42) |
| Loss of therapeutic response | 4 | 5.95(2.21,16.07) | 5.90(4.92,6.89) | 16.05 | 2.54(1.23) |
| White blood cell count decreased | 3 | 21.93(6.81,70.66) | 21.78(20.62,22.94) | 56.06 | 4.36(2.86) |
| Alanine aminotransferase increased | 3 | 25.59(7.90,82.90) | 25.41(24.24,26.58) | 65.67 | 4.57(3.06) |
| Chills | 3 | 4.53(1.44,14.20) | 4.50(3.37,5.64) | 8.09 | 2.16(0.70) |
| Visual impairment | 3 | 12.64(3.98,40.14) | 12.55(11.41,13.70) | 30.83 | 3.60(2.12) |
| Macular oedema | 3 | 82.69(23.47,291.30) | 82.09(80.84,83.34) | 195.26 | 6.06(4.45) |
| Palpitations | 3 | 4.65(1.48,14.57) | 4.62(3.48,5.75) | 8.41 | 2.19(0.73) |
