# Supplementary material for: Safety of ozanimod and etrasimod in ulcerative colitis: A disproportionality analysis of the Food and Drug Administration Adverse Event Reporting System database
Source: Medicine (Baltimore). 2026 Aug 21;105(34):e50262. doi: 10.1097/MD.0000000000050262 (PMC13502775; doi:10.1097/MD.0000000000050262)
Supplement: Supplementary file 9 [file medi-105-e50262-s009.docx]

**Supplementary Table S11. Adverse event signals of Ozanimod and etrasimod at the SOC level following the removal of reports of the top 30 concomitant medications and common immunosuppressants.**

| **Ozanimod** | | | | | |
| --- | --- | --- | --- | --- | --- |
| **SOC** | **n** | **ROR(95%Cl)** | **PRR(95%Cl)** | **χ²** | **IC(IC025)** |
| Gastrointestinal disorders | 710 | 1.36(1.25,1.48) | 1.28(1.2,1.36) | 51.86 | 0.35(0.23) |
| General disorders and administration site conditions | 617 | 1.19(1.08,1.30) | 1.15(1.07,1.23) | 14.11 | 0.20(0.07) |
| **Nervous system disorders** * | 250 | 2.07(1.82,2.36) | 2.00(1.78,2.25) | 124.66 | 0.97(0.78) |
| Musculoskeletal and connective tissue disorders | 173 | 1.15(0.98,1.34) | 1.14(0.98,1.31) | 2.97 | 0.18(-0.04) |
| Investigations | 166 | 0.69(0.59,0.81) | 0.71(0.61,0.82) | 21.99 | -0.5(-0.73) |
| Infections and infestations | 155 | 0.66(0.56,0.78) | 0.68(0.58,0.79) | 25.65 | -0.56(-0.79) |
| Respiratory, thoracic and mediastinal disorders | 118 | 1.14(0.95,1.37) | 1.14(0.95,1.36) | 1.99 | 0.18(-0.09) |
| Injury, poisoning and procedural complications | 111 | 0.23(0.19,0.28) | 0.26(0.21,0.31) | 276.63 | -1.95(-2.22) |
| Vascular disorders | 107 | 1.71(1.41,2.07) | 1.68(1.39,2.03) | 29.74 | 0.74(0.45) |
| Skin and subcutaneous tissue disorders | 105 | 0.91(0.75,1.11) | 0.91(0.76,1.10) | 0.89 | -0.13(-0.41) |
| **Eye disorders** * | 103 | 3.51(2.87,4.28) | 3.42(2.82,4.15) | 173.32 | 1.75(1.42) |
| Psychiatric disorders | 70 | 1.00(0.78,1.26) | 1.00(0.79,1.26) | 0.00 | -0.01(-0.35) |
| **Cardiac disorders** * | 57 | 2.06(1.60,2.66) | 2.03(1.59,2.61) | 26.29 | 0.95(0.54) |
| Renal and urinary disorders | 42 | 1.85(1.36,2.52) | 1.84(1.36,2.49) | 16.00 | 0.87(0.40) |
| Metabolism and nutrition disorders | 35 | 0.85(0.61,1.19) | 0.85(0.61,1.19) | 0.88 | -0.23(-0.71) |
| Immune system disorders | 23 | 1.03(0.68,1.56) | 1.03(0.68,1.55) | 0.02 | 0.04(-0.55) |
| Neoplasms benign, malignant and unspecified (incl cysts and polyps) | 23 | 0.66(0.44,1.00) | 0.67(0.44,1.00) | 3.89 | -0.58(-1.16) |
| Blood and lymphatic system disorders | 22 | 1.12(0.73,1.70) | 1.12(0.73,1.69) | 0.26 | 0.16(-0.46) |
| Surgical and medical procedures | 16 | 0.38(0.23,0.63) | 0.39(0.24,0.63) | 15.71 | -1.36(-2.01) |
| Social circumstances | 15 | 1.00(0.60,1.66) | 1.00(0.60,1.66) | 0.00 | 0(-0.73) |
| Reproductive system and breast disorders | 11 | 0.99(0.54,1.79) | 0.99(0.55,1.78) | 0.00 | -0.02(-0.86) |
| Product issues | 9 | 0.40(0.21,0.77) | 0.40(0.21,0.77) | 8.09 | -1.31(-2.14) |
| Ear and labyrinth disorders | 7 | 0.76(0.36,1.61) | 0.76(0.36,1.60) | 0.51 | -0.39(-1.37) |
| Hepatobiliary disorders | 6 | 0.33(0.15,0.73) | 0.33(0.15,0.73) | 8.26 | -1.6(-2.55) |
| Pregnancy, puerperium and perinatal conditions | 3 | 0.23(0.07,0.71) | 0.23(0.07,0.71) | 7.86 | -2.12(-3.26) |
| Endocrine disorders | 1 | 0.33(0.05,2.32) | 0.33(0.05,2.32) | 1.40 | -1.61(-3.07) |
| **Etrasimod** | | | | | |
| **SOC** | **n** | **ROR(95%Cl)** | **PRR(95%Cl)** | **χ²** | **IC(IC025)** |
| General disorders and administration site conditions | 124 | 1.99(1.61,2.46) | 1.70(1.46,1.97) | 42.82 | 0.76(0.45) |
| Gastrointestinal disorders | 68 | 0.84(0.64,1.08) | 0.86(0.69,1.07) | 1.84 | -0.21(-0.59) |
| **Nervous system disorders** * | 55 | 3.34(2.52,4.45) | 3.03(2.37,3.89) | 77.99 | 1.60(1.13) |
| **Eye disorders** * | 32 | 7.88(5.48,11.32) | 7.35(5.25,10.28) | 174.72 | 2.86(2.08) |
| Investigations | 28 | 0.90(0.62,1.33) | 0.91(0.64,1.30) | 0.26 | -0.13(-0.69) |
| Skin and subcutaneous tissue disorders | 22 | 1.33(0.86,2.04) | 1.31(0.87,1.97) | 1.68 | 0.39(-0.25) |
| Musculoskeletal and connective tissue disorders | 15 | 0.66(0.39,1.10) | 0.67(0.41,1.10) | 2.56 | -0.58(-1.28) |
| Injury, poisoning and procedural complications | 13 | 0.19(0.11,0.33) | 0.22(0.13,0.37) | 43.28 | -2.21(-2.91) |
| Infections and infestations | 12 | 0.33(0.18,0.58) | 0.35(0.20,0.61) | 16.02 | -1.52(-2.27) |
| **Cardiac disorders** * | 9 | 2.20(1.13,4.26) | 2.17(1.14,4.15) | 5.74 | 1.12(0.03) |
| Vascular disorders | 7 | 0.86(0.41,1.83) | 0.87(0.42,1.81) | 0.15 | -0.21(-1.22) |
| Psychiatric disorders | 7 | 0.68(0.32,1.45) | 0.69(0.33,1.44) | 1.00 | -0.53(-1.51) |
| Metabolism and nutrition disorders | 7 | 1.19(0.56,2.51) | 1.18(0.57,2.47) | 0.20 | 0.24(-0.83) |
| Blood and lymphatic system disorders | 5 | 1.78(0.74,4.31) | 1.77(0.74,4.24) | 1.69 | 0.82(-0.54) |
| Hepatobiliary disorders | 3 | 1.07(0.34,3.35) | 1.07(0.35,3.32) | 0.01 | 0.1(-1.38) |
| Respiratory, thoracic and mediastinal disorders | 3 | 0.19(0.06,0.60) | 0.20(0.06,0.62) | 9.99 | -2.32(-3.45) |
| Endocrine disorders | 2 | 4.93(1.22,19.91) | 4.91(1.22,19.71) | 6.17 | 2.28(-0.6) |
| Renal and urinary disorders | 2 | 0.64(0.16,2.58) | 0.64(0.16,2.57) | 0.39 | -0.63(-2.13) |
| Immune system disorders | 1 | 0.30(0.04,2.15) | 0.30(0.04,2.15) | 1.61 | -1.72(-3.15) |
| Surgical and medical procedures | 1 | 0.17(0.02,1.23) | 0.18(0.02,1.24) | 3.93 | -2.51(-3.79) |
| General disorders and administration site conditions | 124 | 1.99(1.61,2.46) | 1.70(1.46,1.97) | 42.82 | 0.76(0.45) |
| Gastrointestinal disorders | 68 | 0.84(0.64,1.08) | 0.86(0.69,1.07) | 1.84 | -0.21(-0.59) |

Note: SOCs marked with an asterisk (*) indicate positive signals at the system organ class level for ozanimod and etrasimod ,which met all three detection algorithms.
