# Supplementary material for: Safety of ozanimod and etrasimod in ulcerative colitis: A disproportionality analysis of the Food and Drug Administration Adverse Event Reporting System database
Source: Medicine (Baltimore). 2026 Aug 21;105(34):e50262. doi: 10.1097/MD.0000000000050262 (PMC13502775; doi:10.1097/MD.0000000000050262)
Supplement: Supplementary file 10 [file medi-105-e50262-s010.docx]

**Supplementary Table S12.** **Positive adverse event signals of ozanimod and etrasimod at the PT level following the removal of reports of the top 30 concomitant medications and common immunosuppressants.**

| **Ozanimod** | | | | | |
| --- | --- | --- | --- | --- | --- |
| **PT** | **n** | **ROR(95%Cl)** | **PRR(95%CI)** | **χ²** | **IC(IC025)** |
| Drug ineffective | 158 | 2.23(1.9,2.62) | 2.17(1.86,2.52) | 99.87 | 1.10(0.85) |
| Fatigue | 106 | 2.52(2.07,3.06) | 2.46(2.04,2.98) | 91.57 | 1.28(0.98) |
| Headache | 75 | 2.55(2.02,3.21) | 2.51(2.00,3.14) | 67.34 | 1.31(0.94) |
| Haemorrhage | 55 | 5.93(4.51,7.80) | 5.84(4.47,7.64) | 210.92 | 2.49(1.97) |
| Nausea | 54 | 2.12(1.61,2.78) | 2.10(1.61,2.74) | 30.71 | 1.05(0.63) |
| Dizziness | 51 | 3.17(2.39,4.19) | 3.13(2.38,4.13) | 72.47 | 1.62(1.15) |
| Dyspnoea | 36 | 2.52(1.81,3.52) | 2.51(1.80,3.48) | 32.03 | 1.31(0.77) |
| Back pain | 34 | 3.35(2.38,4.72) | 3.32(2.37,4.66) | 53.87 | 1.70(1.12) |
| Abdominal pain upper | 30 | 2.40(1.67,3.45) | 2.38(1.66,3.41) | 23.69 | 1.24(0.65) |
| Vision blurred | 26 | 8.65(5.8,12.90) | 8.58(5.77,12.75) | 162.40 | 3.01(2.10) |
| Hypertension | 23 | 2.47(1.63,3.74) | 2.46(1.63,3.71) | 19.59 | 1.28(0.60) |
| Visual impairment | 21 | 7.78(5.00,12.12) | 7.73(4.98,12.01) | 115.60 | 2.87(1.87) |
| Palpitations | 20 | 5.17(3.30,8.10) | 5.14(3.29,8.03) | 63.99 | 2.31(1.41) |
| Peripheral swelling | 20 | 2.77(1.77,4.32) | 2.75(1.77,4.28) | 21.88 | 1.44(0.69) |
| Disturbance in attention | 17 | 27.89(16.42,47.37) | 27.73(16.37,46.99) | 354.49 | 4.50(2.61) |
| Muscle spasms | 15 | 2.06(1.23,3.43) | 2.05(1.23,3.41) | 7.96 | 1.02(0.20) |
| White blood cell count decreased | 15 | 12.12(7.12,20.65) | 12.07(7.10,20.51) | 138.13 | 3.46(2.00) |
| Feeling abnormal | 14 | 2.43(1.43,4.13) | 2.42(1.43,4.11) | 11.47 | 1.26(0.37) |
| Chest pain | 14 | 2.01(1.18,3.41) | 2.01(1.18,3.40) | 6.95 | 0.99(0.14) |
| Maternal exposure during pregnancy | 14 | 2.01(1.18,3.41) | 2.00(1.18,3.39) | 6.90 | 0.99(0.14) |
| Lymphocyte count decreased | 13 | 37.4(20.02,69.87) | 37.24(19.98,69.43) | 348.18 | 4.83(2.39) |
| Migraine | 12 | 2.00(1.13,3.54) | 2.00(1.13,3.53) | 5.88 | 0.99(0.07) |
| Macular oedema | 12 | 128.65(56.72,291.80) | 128.14(56.58,290.16) | 724.00 | 5.95(2.44) |
| Hepatic enzyme increased | 12 | 2.64(1.49,4.69) | 2.63(1.49,4.66) | 11.92 | 1.38(0.40) |
| Pollakiuria | 11 | 12.96(6.95,24.19) | 12.92(6.94,24.06) | 109.02 | 3.55(1.75) |
| Influenza like illness | 11 | 3.05(1.68,5.56) | 3.05(1.68,5.54) | 14.77 | 1.58(0.51) |
| Confusional state | 10 | 2.59(1.38,4.86) | 2.59(1.38,4.84) | 9.54 | 1.35(0.28) |
| Diarrhoea haemorrhagic | 10 | 2.11(1.13,3.95) | 2.11(1.13,3.94) | 5.74 | 1.06(0.04) |
| Bowel movement irregularity | 10 | 4.44(2.36,8.36) | 4.43(2.36,8.33) | 25.63 | 2.11(0.83) |
| Neck pain | 9 | 5.61(2.87,10.95) | 5.59(2.87,10.91) | 32.42 | 2.43(0.96) |
| Cerebrovascular accident | 9 | 2.58(1.33,5.01) | 2.58(1.33,4.99) | 8.52 | 1.35(0.21) |
| Oedema peripheral | 8 | 3.37(1.67,6.82) | 3.37(1.67,6.80) | 12.96 | 1.72(0.41) |
| Therapy interrupted | 8 | 4.83(2.38,9.80) | 4.82(2.38,9.76) | 23.27 | 2.22(0.74) |
| Epistaxis | 6 | 4.58(2.03,10.37) | 4.58(2.03,10.34) | 16.15 | 2.15(0.45) |
| Cardiac flutter | 6 | 35.31(14.17,87.98) | 35.24(14.16,87.68) | 153.54 | 4.77(1.29) |
| Treatment noncompliance | 6 | 3.04(1.35,6.85) | 3.04(1.35,6.83) | 8.00 | 1.58(0.11) |
| Muscular weakness | 6 | 3.31(1.47,7.46) | 3.31(1.47,7.44) | 9.41 | 1.70(0.18) |
| Dysuria | 5 | 5.40(2.20,13.23) | 5.39(2.20,13.20) | 17.09 | 2.38(0.40) |
| Neutropenia | 5 | 8.65(3.49,21.46) | 8.64(3.49,21.40) | 31.46 | 3.02(0.66) |
| Photophobia | 5 | 8.78(3.54,21.80) | 8.77(3.54,21.73) | 32.02 | 3.04(0.67) |
| Brain fog | 4 | 5.17(1.90,14.08) | 5.16(1.90,14.04) | 12.86 | 2.32(0.14) |
| Leukopenia | 4 | 6.92(2.52,18.97) | 6.91(2.52,18.93) | 19.10 | 2.72(0.29) |
| Arrhythmia | 4 | 4.70(1.73,12.79) | 4.70(1.73,12.76) | 11.20 | 2.19(0.08) |
| Micturition urgency | 4 | 5.74(2.10,15.66) | 5.73(2.10,15.62) | 14.89 | 2.46(0.20) |
| Urinary incontinence | 4 | 8.71(3.15,24.07) | 8.7(3.15,24.01) | 25.39 | 3.03(0.39) |
| Multiple sclerosis | 4 | 7.47(2.72,20.53) | 7.46(2.72,20.48) | 21.04 | 2.82(0.33) |
| Transient ischaemic attack | 4 | 5.34(1.96,14.57) | 5.34(1.96,14.53) | 13.50 | 2.36(0.16) |
| Renal cancer | 3 | 10.08(3.10,32.78) | 10.07(3.10,32.72) | 22.57 | 3.23(0.07) |
| Lymphopenia | 3 | 9.28(2.86,30.08) | 9.27(2.86,30.02) | 20.52 | 3.12(0.05) |
| Urine abnormality | 3 | 39.19(10.60,144.84) | 39.15(10.60,144.55) | 83.65 | 4.89(0.20) |
| Norovirus infection | 3 | 11.02(3.37,36.01) | 11.01(3.37,35.94) | 24.97 | 3.34(0.10) |
| Mental status changes | 3 | 58.79(14.70,235.17) | 58.73(14.69,234.72) | 113.49 | 5.30(0.18) |
| **Etrasimod** | | | | | |
| **PT** | **n** | **ROR(95%Cl)** | **PRR(95%CI)** | **χ²** | **IC(IC025)** |
| Drug ineffective | 38 | 4.30(3.08,6.01) | 4.00(2.95,5.42) | 86.84 | 1.99(1.40) |
| Headache | 21 | 5.13(3.30,7.98) | 4.92(3.24,7.49) | 65.71 | 2.29(1.42) |
| Condition aggravated | 20 | 2.05(1.31,3.21) | 2.00(1.30,3.07) | 10.17 | 1.00(0.28) |
| Dizziness | 15 | 7.01(4.17,11.78) | 6.80(4.12,11.21) | 73.52 | 2.75(1.56) |
| Vision blurred | 10 | 23.49(12.37,44.61) | 22.95(12.26,42.95) | 200.76 | 4.46(2.01) |
| Nausea | 9 | 2.62(1.35,5.07) | 2.58(1.35,4.94) | 8.75 | 1.36(0.22) |
| Pain | 8 | 2.49(1.24,5.03) | 2.46(1.24,4.90) | 6.98 | 1.30(0.10) |
| Therapeutic product effect incomplete | 7 | 5.14(2.43,10.89) | 5.07(2.42,10.61) | 22.71 | 2.33(0.70) |
| Bradycardia | 5 | 36.01(14.45,89.71) | 35.59(14.43,87.76) | 156.77 | 5.06(1.15) |
| Hypoaesthesia | 4 | 4.24(1.58,11.39) | 4.21(1.58,11.20) | 9.72 | 2.06(0.05) |
| Hepatic enzyme increased | 4 | 5.25(1.95,14.13) | 5.21(1.95,13.89) | 13.49 | 2.37(0.19) |
| Visual impairment | 4 | 11.52(4.26,31.20) | 11.42(4.26,30.63) | 37.20 | 3.48(0.56) |
| Type 2 diabetes mellitus | 4 | 7.47(2.77,20.16) | 7.41(2.77,19.80) | 21.89 | 2.87(0.38) |
| Diabetic eye disease | 4 | 1983.41(221.21,17783.91) | 1964.35(220.01,17538.42) | 1569.92 | 8.62(0.55) |
| Influenza like illness | 4 | 7.26(2.69,19.56) | 7.20(2.69,19.22) | 21.06 | 2.83(0.37) |
| Loss of therapeutic response | 4 | 4.52(1.68,12.15) | 4.48(1.68,11.95) | 10.76 | 2.15(0.09) |
