# Supplementary material for: Safety of ozanimod and etrasimod in ulcerative colitis: A disproportionality analysis of the Food and Drug Administration Adverse Event Reporting System database
Source: Medicine (Baltimore). 2026 Aug 21;105(34):e50262. doi: 10.1097/MD.0000000000050262 (PMC13502775; doi:10.1097/MD.0000000000050262)
Supplement: Supplementary file 11 [file medi-105-e50262-s011.docx]

**Supplementary Table S13. Characteristics of serious cases related to ozanimod and etrasimod.**

| **Characteristics** | **Ozanimod n(%)** | **Etrasimod n(%)** |
| --- | --- | --- |
| Number of cases | 163 | 24 |
| Outcome types | | |
| Death | 14(8.59%) | 0(0.00%) |
| Life-Threatening | 15(9.20%) | 0(0.00%) |
| Hospitalization | 128(78.53%) | 23(95.83%) |
| Disability | 6(3.68%) | 1(4.17%) |
| Gender | | |
| Female | 83(50.92%) | 14(58.33%) |
| Male | 77(47.24%) | 10(41.67%) |
| missing | 3(1.84%) | 0(0.00%) |
| Age | | |
| Median | 50 | 52 |
| Interquartile range | 34 ,63 | 39 ,62.5 |
| Time-to-onset | | |
| Median | 46.5 | Insufficient data |
| Interquartile range | 10.5 ,198.5 | Insufficient data |
| **Top 3 adverse events of SOC level of each outcome** | | |
| Death | General disorders and administration site conditions(n=11) | Not available |
|  | Gastrointestinal disorders(n=1) |  |
|  | Nervous system disorders(n=1) |  |
| Life-Threatening | Infections and infestations(n=3) | Not available |
|  | Cardiac disorders(n=2) |  |
|  | Nervous system disorders(n=2) |  |
| Hospitalization | Gastrointestinal disorders(n=36) | Infections and infestations(n=4) |
|  | Infections and infestations(n=19) | Nervous system disorders(n=4) |
|  | General disorders and administration site conditions(n=15) | General disorders and administration site conditions(n=4) |
| Disability | Eye disorders(n=3) | Investigations(n=1) |
|  | General disorders and administration site conditions(n=2) | Not available |
|  | Social circumstances(n=1) |  |
