# Supplementary material for: Safety of ozanimod and etrasimod in ulcerative colitis: A disproportionality analysis of the Food and Drug Administration Adverse Event Reporting System database
Source: Medicine (Baltimore). 2026 Aug 21;105(34):e50262. doi: 10.1097/MD.0000000000050262 (PMC13502775; doi:10.1097/MD.0000000000050262)
Supplement: Supplementary file 12 [file medi-105-e50262-s012.docx]

**Supplementary Table S2. The REporting of A Disproportionality analysis for drUg Safety signal detection using individual case safety reports in PharmacoVigilance (READUS-PV) checklist for abstracts.**

| **Section and topic** | **Item** | **Checklist item** | **Reported on Page/Lines** |
| --- | --- | --- | --- |
| **Background** | 1a | State the aim/rationale for performing the study. | Page 3, Lines 55 |
|  | 1b | Specify the adverse event(s) and/or the drug(s) under study, when applicable. | Page 3, Lines 54-57 |
|  | 1c | Specify the specific population or setting, when applicable. | Page 3, Lines 55-57 |
|  | 1d | Identify the study as a “disproportionality analysis” and specify the type of data used. | Page 3, Lines 55-57 |
| **Methods** | 2a | Specify the name of the database(s) used and the type of access. | Page 3, Lines 55-57 |
|  | 2b | Specify the timeframe and geographical region, when applicable. | Page 3, Lines 55-57 |
|  | 2c | Specify the disproportionality measure(s) used and their statistical significance threshold(s). | Page 3, Lines 58-60 |
|  | 2d | Specify if a case-by-case analysis is performed. | Not applicable |
|  | 2e | State the aim/rationale for performing the study. | Page 3, Lines 57 |
| **Results** | 3 | Report main findings including their precision (e.g., 95% confidence intervals), together with a short summary of the case-by-case analysis. | Page 3, Lines 61-69 |
| **Conclusion** | 4a | Clearly report key conclusions. | Page 3, Lines 65-66 |
|  | 4b | Acknowledge that the disproportionality analysis is a hypothesis generating or refinement approach. | Page 3, Lines 71-72 |
|  | 4c | State the implications and clinical relevance of the findings. | Page 3, Lines 69-71 |
