# Supplementary material for: Safety of ozanimod and etrasimod in ulcerative colitis: A disproportionality analysis of the Food and Drug Administration Adverse Event Reporting System database
Source: Medicine (Baltimore). 2026 Aug 21;105(34):e50262. doi: 10.1097/MD.0000000000050262 (PMC13502775; doi:10.1097/MD.0000000000050262)
Supplement: Supplementary file 13 [file medi-105-e50262-s013.docx]

**Supplementary Table S8. Positive adverse event signals at the PT level for ozanimod and etrasimod using the full FAERS database.**

| **Ozanimod** | | | | | |
| --- | --- | --- | --- | --- | --- |
| **PT** | **n** | **ROR(95%Cl)** | **PRR(95%CI)** | **χ²** | **IC(IC025)** |
| Fatigue | 623 | 3.30(3.05,3.58) | 3.21(3.13,3.29) | 957.38 | 1.68(1.56) |
| Headache | 446 | 3.44(3.13,3.78) | 3.37(3.28,3.46) | 748.12 | 1.75(1.61) |
| Dizziness | 299 | 2.97(2.65,3.33) | 2.93(2.82,3.04) | 381.38 | 1.55(1.38) |
| Back pain | 194 | 3.90(3.38,4.49) | 3.86(3.72,4.00) | 411.08 | 1.95(1.74) |
| Fall | 146 | 2.04(1.74,2.41) | 2.03(1.87,2.20) | 77.01 | 1.02(0.78) |
| Hypoaesthesia | 134 | 4.29(3.62,5.09) | 4.26(4.10,4.43) | 334.50 | 2.09(1.84) |
| Hypertension | 116 | 2.39(1.99,2.87) | 2.38(2.20,2.56) | 93.25 | 1.25(0.98) |
| Depression | 111 | 2.77(2.3,3.34) | 2.76(2.57,2.94) | 124.54 | 1.46(1.19) |
| Urinary tract infection | 103 | 2.46(2.03,2.99) | 2.45(2.26,2.65) | 88.86 | 1.29(1.01) |
| Gait disturbance | 98 | 2.39(1.96,2.91) | 2.38(2.18,2.58) | 78.37 | 1.25(0.96) |
| Paraesthesia | 96 | 3.01(2.46,3.68) | 3.00(2.80,3.20) | 127.91 | 1.58(1.29) |
| Balance disorder | 95 | 5.49(4.49,6.72) | 5.47(5.26,5.67) | 345.53 | 2.45(2.15) |
| Peripheral swelling | 95 | 2.11(1.73,2.58) | 2.11(1.90,2.31) | 55.18 | 1.07(0.78) |
| White blood cell count decreased | 95 | 3.35(2.74,4.10) | 3.33(3.13,3.53) | 155.10 | 1.73(1.44) |
| Blood pressure increased | 94 | 2.51(2.05,3.07) | 2.50(2.29,2.70) | 84.32 | 1.32(1.02) |
| Lymphocyte count decreased | 94 | 20.36(16.60,24.98) | 20.24(20.04,20.45) | 1693.38 | 4.32(4.02) |
| Abdominal pain upper | 94 | 2.11(1.72,2.59) | 2.10(1.90,2.30) | 54.47 | 1.07(0.77) |
| Muscle spasms | 91 | 2.48(2.02,3.05) | 2.47(2.27,2.68) | 79.69 | 1.30(1.00) |
| Memory impairment | 90 | 2.87(2.33,3.53) | 2.86(2.65,3.06) | 108.72 | 1.51(1.21) |
| Visual impairment | 89 | 2.86(2.32,3.53) | 2.85(2.65,3.06) | 107.11 | 1.51(1.21) |
| Migraine | 86 | 3.72(3.01,4.60) | 3.71(3.49,3.92) | 169.68 | 1.89(1.58) |
| Vision blurred | 85 | 3.05(2.47,3.78) | 3.04(2.83,3.25) | 116.45 | 1.60(1.29) |
| Alopecia | 85 | 2.03(1.64,2.51) | 2.02(1.81,2.23) | 43.95 | 1.01(0.70) |
| Haemorrhage | 78 | 3.32(2.66,4.15) | 3.31(3.09,3.53) | 125.56 | 1.72(1.40) |
| Maternal exposure during pregnancy | 78 | 2.64(2.12,3.30) | 2.63(2.41,2.86) | 79.07 | 1.40(1.07) |
| Palpitations | 75 | 3.27(2.61,4.11) | 3.26(3.04,3.49) | 117.58 | 1.70(1.37) |
| Heart rate decreased | 74 | 7.32(5.82,9.20) | 7.29(7.06,7.52) | 399.55 | 2.86(2.52) |
| Stress | 71 | 3.87(3.06,4.88) | 3.85(3.62,4.08) | 149.66 | 1.94(1.60) |
| Lymphopenia | 70 | 18.79(14.84,23.81) | 18.71(18.48,18.95) | 1157.02 | 4.21(3.86) |
| Haematochezia | 70 | 4.60(3.63,5.82) | 4.58(4.35,4.82) | 195.52 | 2.19(1.85) |
| Hepatic enzyme increased | 69 | 3.88(3.06,4.92) | 3.87(3.63,4.11) | 146.63 | 1.95(1.60) |
| Disturbance in attention | 62 | 5.93(4.62,7.61) | 5.91(5.66,6.16) | 251.93 | 2.56(2.19) |
| Muscular weakness | 57 | 2.48(1.91,3.21) | 2.47(2.21,2.73) | 49.94 | 1.30(0.92) |
| Musculoskeletal stiffness | 55 | 2.41(1.85,3.15) | 2.41(2.15,2.67) | 45.34 | 1.27(0.88) |
| Treatment noncompliance | 45 | 3.41(2.54,4.57) | 3.40(3.11,3.69) | 76.15 | 1.76(1.34) |
| Tremor | 45 | 2.02(1.63,2.41) | 2.01(1.72,2.30) | 28.14 | 1.01(0.78) |
| Contusion | 44 | 2.03(1.51,2.72) | 2.02(1.73,2.32) | 22.76 | 1.02(0.58) |
| Rectal haemorrhage | 42 | 5.43(4.01,7.36) | 5.42(5.12,5.72) | 150.80 | 2.43(1.99) |
| Herpes zoster | 39 | 2.83(2.07,3.88) | 2.82(2.51,3.14) | 45.90 | 1.50(1.04) |
| Therapy non-responder | 36 | 2.34(1.68,3.24) | 2.33(2.01,2.66) | 27.38 | 1.22(0.74) |
| Flatulence | 34 | 2.91(2.08,4.08) | 2.91(2.57,3.24) | 42.51 | 1.54(1.05) |
| Pollakiuria | 32 | 3.77(2.66,5.34) | 3.76(3.42,4.11) | 64.81 | 1.91(1.41) |
| Urinary incontinence | 31 | 5.31(3.73,7.56) | 5.30(4.95,5.66) | 107.84 | 2.40(1.89) |
| Limb discomfort | 30 | 3.70(2.59,5.30) | 3.70(3.34,4.06) | 58.90 | 1.88(1.36) |
| Neuralgia | 30 | 4.90(3.43,7.02) | 4.90(4.54,5.25) | 92.71 | 2.29(1.77) |
| Leukopenia | 28 | 2.55(1.76,3.69) | 2.55(2.18,2.92) | 26.26 | 1.35(0.81) |
| Neck pain | 27 | 2.20(1.51,3.21) | 2.20(1.82,2.57) | 17.60 | 1.13(0.59) |
| Cognitive disorder | 26 | 2.43(1.65,3.57) | 2.43(2.04,2.81) | 21.81 | 1.28(0.72) |
| Vertigo | 24 | 2.10(1.41,3.13) | 2.10(1.70,2.50) | 13.78 | 1.07(0.49) |
| Frequent bowel movements | 23 | 2.78(1.84,4.18) | 2.77(2.36,3.18) | 26.04 | 1.47(0.88) |
| Macular oedema | 23 | 21.64(14.33,32.69) | 21.61(21.20,22.02) | 444.62 | 4.41(3.82) |
| Insurance issue | 23 | 3.65(2.42,5.50) | 3.65(3.24,4.06) | 44.09 | 1.86(1.27) |
| Upper respiratory tract infection | 22 | 2.17(1.43,3.29) | 2.16(1.75,2.58) | 13.77 | 1.11(0.51) |
| Transient ischaemic attack | 22 | 4.28(2.81,6.50) | 4.27(3.85,4.69) | 54.94 | 2.09(1.49) |
| Liver function test increased | 21 | 3.22(2.10,4.95) | 3.22(2.79,3.65) | 32.08 | 1.68(1.07) |
| Blindness | 20 | 2.11(1.36,3.27) | 2.11(1.67,2.54) | 11.61 | 1.07(0.44) |
| Central nervous system lesion | 20 | 9.67(6.23,15.03) | 9.66(9.22,10.10) | 154.20 | 3.26(2.63) |
| Optic neuritis | 20 | 12.37(7.96,19.22) | 12.36(11.92,12.80) | 206.81 | 3.61(2.98) |
| Brain fog | 20 | 2.99(1.93,4.64) | 2.99(2.55,3.43) | 26.39 | 1.58(0.95) |
| Bladder disorder | 19 | 9.09(5.79,14.29) | 9.08(8.63,9.54) | 135.76 | 3.17(2.53) |
| Covid-19 pneumonia | 18 | 2.31(1.46,3.67) | 2.31(1.85,2.77) | 13.37 | 1.21(0.54) |
| Bowel movement irregularity | 17 | 7.55(4.69,12.17) | 7.54(7.07,8.02) | 95.96 | 2.91(2.23) |
| Blood pressure abnormal | 16 | 2.93(1.79,4.78) | 2.93(2.44,3.42) | 20.24 | 1.55(0.85) |
| Eye disorder | 16 | 2.09(1.28,3.42) | 2.09(1.60,2.58) | 9.10 | 1.06(0.36) |
| Defaecation urgency | 16 | 6.97(4.26,11.4) | 6.96(6.47,7.45) | 81.29 | 2.79(2.09) |
| Chromaturia | 15 | 3.89(2.34,6.46) | 3.89(3.38,4.39) | 32.06 | 1.96(1.23) |
| Dysuria | 15 | 2.09(1.26,3.46) | 2.09(1.58,2.59) | 8.46 | 1.06(0.34) |
| Movement disorder | 15 | 2.29(1.38,3.79) | 2.28(1.78,2.79) | 10.81 | 1.19(0.47) |
| Muscle spasticity | 14 | 5.82(3.44,9.83) | 5.81(5.29,6.34) | 55.53 | 2.53(1.79) |
| Hypersomnia | 13 | 2.07(1.20,3.57) | 2.07(1.53,2.61) | 7.18 | 1.05(0.28) |
| Oral herpes | 13 | 2.38(1.38,4.10) | 2.38(1.84,2.92) | 10.39 | 1.25(0.48) |
| Pregnancy | 13 | 5.23(3.03,9.02) | 5.22(4.68,5.77) | 44.23 | 2.38(1.61) |
| Cardiac flutter | 12 | 8.7(4.93,15.35) | 8.69(8.13,9.26) | 81.16 | 3.11(2.31) |
| Multiple allergies | 12 | 5.43(3.08,9.57) | 5.42(4.86,5.99) | 43.13 | 2.43(1.63) |
| Concussion | 12 | 6.39(3.62,11.26) | 6.38(5.81,6.95) | 54.19 | 2.67(1.87) |
| Orthostatic hypotension | 12 | 3.12(1.77,5.51) | 3.12(2.56,3.69) | 17.28 | 1.64(0.84) |
| Diplopia | 12 | 2.23(1.26,3.92) | 2.23(1.66,2.79) | 8.09 | 1.15(0.35) |
| Tooth disorder | 12 | 2.57(1.46,4.53) | 2.57(2.00,3.13) | 11.46 | 1.36(0.56) |
| Gastroenteritis viral | 12 | 2.79(1.58,4.92) | 2.79(2.22,3.36) | 13.77 | 1.48(0.68) |
| Pain in jaw | 11 | 2.23(1.24,4.04) | 2.23(1.64,2.82) | 7.48 | 1.16(0.32) |
| Sleep apnoea syndrome | 11 | 2.59(1.43,4.67) | 2.58(1.99,3.18) | 10.66 | 1.37(0.53) |
| Neoplasm | 11 | 3.60(1.99,6.50) | 3.59(3.00,4.19) | 20.54 | 1.84(1.01) |
| Photophobia | 11 | 2.54(1.40,4.58) | 2.53(1.94,3.13) | 10.20 | 1.34(0.51) |
| Product distribution issue | 11 | 2.83(1.57,5.12) | 2.83(2.24,3.42) | 13.02 | 1.50(0.67) |
| General physical condition abnormal | 11 | 5.88(3.25,10.64) | 5.88(5.29,6.47) | 44.36 | 2.55(1.72) |
| Immune system disorder | 10 | 3.03(1.63,5.64) | 3.03(2.41,3.65) | 13.58 | 1.60(0.73) |
| Respiratory tract congestion | 10 | 2.86(1.54,5.32) | 2.86(2.24,3.48) | 12.07 | 1.51(0.64) |
| Abnormal faeces | 10 | 4.64(2.49,8.63) | 4.63(4.01,5.26) | 28.41 | 2.21(1.34) |
| Anal incontinence | 10 | 3.49(1.88,6.50) | 3.49(2.87,4.11) | 17.74 | 1.80(0.93) |
| Micturition urgency | 10 | 4.17(2.24,7.77) | 4.17(3.55,4.79) | 24.05 | 2.06(1.19) |
| Magnetic resonance imaging head abnormal | 10 | 22.8(12.20,42.62) | 22.79(22.16,23.41) | 204.69 | 4.49(3.61) |
| Peroneal nerve palsy | 10 | 9.50(5.10,17.71) | 9.50(8.88,10.12) | 75.49 | 3.24(2.36) |
| Diarrhoea haemorrhagic | 10 | 4.85(2.60,9.02) | 4.84(4.22,5.46) | 30.38 | 2.27(1.40) |
| Immunodeficiency | 10 | 2.14(1.15,3.99) | 2.14(1.52,2.76) | 6.09 | 1.10(0.23) |
| Dizziness postural | 9 | 3.62(1.88,6.97) | 3.62(2.97,4.27) | 17.02 | 1.85(0.94) |
| Muscle twitching | 9 | 2.10(1.09,4.04) | 2.10(1.45,2.76) | 5.20 | 1.07(0.16) |
| Hemiparesis | 9 | 3.31(1.72,6.36) | 3.30(2.65,3.96) | 14.43 | 1.72(0.81) |
| Uterine disorder | 9 | 26.48(13.69,51.25) | 26.47(25.81,27.13) | 216.12 | 4.70(3.78) |
| Lymphocyte count abnormal | 9 | 27.89(14.41,53.99) | 27.87(27.21,28.53) | 228.23 | 4.77(3.85) |
| Muscle tightness | 9 | 2.97(1.54,5.71) | 2.97(2.31,3.62) | 11.70 | 1.57(0.65) |
| Hepatic enzyme abnormal | 9 | 7.74(4.02,14.90) | 7.73(7.08,8.39) | 52.44 | 2.94(2.03) |
| Feeling of despair | 9 | 8.70(4.51,16.75) | 8.69(8.04,9.35) | 60.84 | 3.11(2.19) |
| Photosensitivity reaction | 8 | 2.36(1.18,4.71) | 2.35(1.66,3.05) | 6.22 | 1.23(0.27) |
| Periorbital swelling | 8 | 3.06(1.53,6.12) | 3.05(2.36,3.75) | 11.03 | 1.61(0.65) |
| Back disorder | 8 | 2.22(1.11,4.45) | 2.22(1.53,2.92) | 5.37 | 1.15(0.19) |
| Vitamin d decreased | 8 | 4.41(2.20,8.84) | 4.41(3.72,5.11) | 21.05 | 2.14(1.17) |
| Spinal disorder | 8 | 3.82(1.91,7.64) | 3.82(3.12,4.51) | 16.57 | 1.93(0.96) |
| Faeces discoloured | 8 | 2.02(1.01,4.04) | 2.02(1.33,2.71) | 4.12 | 1.01(0.05) |
| Decreased activity | 8 | 2.45(1.23,4.91) | 2.45(1.76,3.15) | 6.87 | 1.29(0.33) |
| Trigeminal neuralgia | 8 | 9.56(4.77,19.17) | 9.56(8.86,10.25) | 60.83 | 3.25(2.28) |
| Coronavirus infection | 8 | 2.63(1.31,5.26) | 2.63(1.93,3.32) | 8.04 | 1.39(0.43) |
| Product supply issue | 8 | 2.29(1.14,4.57) | 2.28(1.59,2.98) | 5.77 | 1.19(0.23) |
| Decreased interest | 8 | 9.27(4.62,18.58) | 9.26(8.57,9.96) | 58.55 | 3.20(2.24) |
| Rotator cuff syndrome | 7 | 2.80(1.33,5.88) | 2.80(2.06,3.54) | 8.08 | 1.48(0.46) |
| Hypoaesthesia oral | 7 | 2.37(1.13,4.97) | 2.37(1.63,3.11) | 5.52 | 1.24(0.22) |
| Temperature intolerance | 7 | 2.73(1.30,5.74) | 2.73(1.99,3.47) | 7.68 | 1.45(0.43) |
| Mucous stools | 7 | 3.89(1.85,8.16) | 3.88(3.14,4.63) | 14.95 | 1.95(0.93) |
| Affective disorder | 7 | 3.75(1.79,7.88) | 3.75(3.01,4.49) | 14.08 | 1.90(0.88) |
| Sensory disturbance | 6 | 2.44(1.09,5.43) | 2.44(1.64,3.24) | 5.08 | 1.28(0.19) |
| Vitreous floaters | 6 | 2.49(1.12,5.55) | 2.49(1.69,3.29) | 5.34 | 1.31(0.22) |
| Extrasystoles | 6 | 5.51(2.47,12.30) | 5.51(4.71,6.31) | 22.07 | 2.46(1.36) |
| Suspected covid-19 | 6 | 3.35(1.50,7.47) | 3.35(2.55,4.15) | 9.87 | 1.74(0.65) |
| Gallbladder disorder | 6 | 2.31(1.04,5.14) | 2.31(1.50,3.11) | 4.43 | 1.20(0.11) |
| Abdominal tenderness | 6 | 7.01(3.14,15.65) | 7.01(6.21,7.81) | 30.75 | 2.80(1.71) |
| Sleep deficit | 6 | 6.66(2.99,14.86) | 6.66(5.86,7.46) | 28.71 | 2.73(1.63) |
| Coordination abnormal | 6 | 3.46(1.55,7.71) | 3.46(2.66,4.26) | 10.46 | 1.79(0.70) |
| Loss of therapeutic response | 6 | 3.78(1.70,8.43) | 3.78(2.98,4.58) | 12.23 | 1.92(0.82) |
| Gastrointestinal motility disorder | 5 | 4.07(1.69,9.80) | 4.07(3.19,4.95) | 11.55 | 2.02(0.84) |
| White blood cell count abnormal | 5 | 2.81(1.17,6.75) | 2.81(1.93,3.68) | 5.80 | 1.49(0.31) |
| Meniscus injury | 5 | 3.24(1.35,7.80) | 3.24(2.36,4.12) | 7.73 | 1.69(0.51) |
| Urinary tract disorder | 5 | 4.83(2.01,11.62) | 4.83(3.95,5.71) | 15.12 | 2.27(1.09) |
| Food poisoning | 5 | 3.39(1.41,8.16) | 3.39(2.52,4.27) | 8.42 | 1.76(0.58) |
| T-lymphocyte count decreased | 5 | 26.78(11.05,64.95) | 26.78(25.89,27.66) | 121.53 | 4.71(3.52) |
| Blood disorder | 5 | 2.78(1.16,6.69) | 2.78(1.90,3.66) | 5.69 | 1.47(0.29) |
| Cd4 lymphocytes decreased | 5 | 11.79(4.89,28.46) | 11.79(10.91,12.67) | 48.93 | 3.55(2.36) |
| Bladder pain | 4 | 6.06(2.27,16.18) | 6.06(5.07,7.04) | 16.80 | 2.59(1.30) |
| Heart rate abnormal | 4 | 2.77(1.04,7.39) | 2.77(1.79,3.75) | 4.51 | 1.47(0.18) |
| Jc polyomavirus test positive | 4 | 7.51(2.81,20.06) | 7.51(6.52,8.49) | 22.42 | 2.90(1.61) |
| Hyperaesthesia teeth | 4 | 7.03(2.63,18.8) | 7.03(6.05,8.02) | 20.59 | 2.81(1.51) |
| Skin abrasion | 4 | 3.04(1.14,8.10) | 3.03(2.05,4.02) | 5.44 | 1.60(0.31) |
| Feeling drunk | 4 | 3.55(1.33,9.47) | 3.55(2.57,4.53) | 7.30 | 1.82(0.53) |
| Auditory disorder | 4 | 8.94(3.34,23.91) | 8.94(7.96,9.92) | 28.02 | 3.15(1.86) |
| Proctalgia | 4 | 2.86(1.07,7.63) | 2.86(1.88,3.84) | 4.83 | 1.51(0.22) |
| Menstruation delayed | 4 | 3.17(1.19,8.46) | 3.17(2.19,4.15) | 5.93 | 1.66(0.37) |
| Iron deficiency | 4 | 2.99(1.12,7.98) | 2.99(2.01,3.97) | 5.29 | 1.58(0.29) |
| Labelled drug-drug interaction issue | 4 | 5.28(1.98,14.1) | 5.28(4.30,6.26) | 13.81 | 2.40(1.10) |
| Infrequent bowel movements | 4 | 7.13(2.67,19.06) | 7.13(6.15,8.11) | 20.97 | 2.83(1.53) |
| Norovirus infection | 4 | 5.81(2.18,15.52) | 5.81(4.83,6.79) | 15.85 | 2.53(1.24) |
| Normal newborn | 4 | 3.65(1.37,9.73) | 3.65(2.66,4.63) | 7.66 | 1.86(0.57) |
| Intervertebral disc disorder | 4 | 5.66(2.12,15.11) | 5.66(4.67,6.64) | 15.26 | 2.49(1.20) |
| Retinal oedema | 4 | 8.51(3.18,22.76) | 8.51(7.53,9.49) | 26.34 | 3.08(1.79) |
| Muscle fatigue | 3 | 3.16(1.02,9.80) | 3.16(2.02,4.29) | 4.41 | 1.66(0.21) |
| Lymph node pain | 3 | 7.17(2.30,22.30) | 7.17(6.03,8.30) | 15.83 | 2.83(1.39) |
| Lactose intolerance | 3 | 5.25(1.69,16.33) | 5.25(4.12,6.39) | 10.29 | 2.39(0.94) |
| Neurological decompensation | 3 | 5.52(1.78,17.17) | 5.52(4.39,6.66) | 11.07 | 2.46(1.01) |
| Plantar fasciitis | 3 | 3.53(1.14,10.97) | 3.53(2.40,4.66) | 5.43 | 1.82(0.37) |
| Traumatic fracture | 3 | 15.18(4.86,47.4) | 15.18(14.04,16.32) | 39.27 | 3.91(2.45) |
| Oral infection | 3 | 3.58(1.15,11.13) | 3.58(2.45,4.72) | 5.57 | 1.84(0.39) |
| Functional gastrointestinal disorder | 3 | 3.67(1.18,11.41) | 3.67(2.54,4.81) | 5.82 | 1.87(0.43) |
| Cardiac discomfort | 3 | 5.52(1.78,17.17) | 5.52(4.39,6.66) | 11.07 | 2.46(1.01) |
| Weight abnormal | 3 | 3.64(1.17,11.31) | 3.64(2.51,4.77) | 5.73 | 1.86(0.42) |
| Sinus headache | 3 | 3.57(1.15,11.09) | 3.57(2.44,4.70) | 5.54 | 1.83(0.39) |
| Nasal septum deviation | 3 | 7.08(2.27,22.01) | 7.07(5.94,8.21) | 15.56 | 2.82(1.37) |
| Penile swelling | 3 | 15.87(5.08,49.56) | 15.87(14.73,17.01) | 41.28 | 3.97(2.52) |
| Clumsiness | 3 | 5.53(1.78,17.20) | 5.53(4.40,6.67) | 11.09 | 2.46(1.02) |
| Head titubation | 3 | 10.86(3.49,33.85) | 10.86(9.72,12.00) | 26.64 | 3.43(1.98) |
| Migraine with aura | 3 | 6.04(1.94,18.79) | 6.04(4.91,7.18) | 12.57 | 2.59(1.14) |
| Cervical vertebral fracture | 3 | 4.00(1.29,12.41) | 4.00(2.86,5.13) | 6.72 | 2.00(0.55) |
| Autoimmune thyroiditis | 3 | 3.21(1.03,9.97) | 3.21(2.08,4.34) | 4.55 | 1.68(0.24) |
| Kaposi's sarcoma | 3 | 3.82(1.23,11.86) | 3.82(2.68,4.95) | 6.22 | 1.93(0.48) |
| Gastroenteritis escherichia coli | 3 | 17.06(5.46,53.31) | 17.06(15.92,18.20) | 44.76 | 4.07(2.62) |
| **Etrasimod** | | | | | |
| **PT** | **n** | **ROR(95%Cl)** | **PRR(95%CI)** | **χ²** | **IC(IC025)** |
| Drug ineffective | 158 | 3.99(3.39,4.70) | 3.74(3.59,3.89) | 324.18 | 1.9(1.66) |
| Condition aggravated | 77 | 7.05(5.62,8.86) | 6.81(6.59,7.03) | 383.50 | 2.77(2.43) |
| Type 2 diabetes mellitus | 66 | 88.70(69.33,113.49) | 85.63(85.39,85.86) | 5485.06 | 6.41(6.05) |
| Headache | 54 | 3.26(2.49,4.28) | 3.20(2.93,3.46) | 82.26 | 1.68(1.28) |
| Diabetic eye disease | 52 | 5134.65(3711.77,7102.97) | 4992.80(4992.48,4993.12) | 185779.31 | 11.8(11.35) |
| Dizziness | 45 | 3.57(2.65,4.79) | 3.51(3.22,3.79) | 81.10 | 1.81(1.38) |
| Haematochezia | 29 | 15.58(10.79,22.49) | 15.35(14.99,15.72) | 389.09 | 3.94(3.41) |
| Malaise | 29 | 2.64(1.83,3.81) | 2.61(2.25,2.98) | 29.07 | 1.39(0.86) |
| Drug interaction | 26 | 6.32(4.29,9.31) | 6.25(5.87,6.63) | 114.86 | 2.64(2.08) |
| Vision blurred | 22 | 6.38(4.19,9.72) | 6.32(5.90,6.73) | 98.64 | 2.66(2.05) |
| Rectal haemorrhage | 18 | 17.13(10.77,27.26) | 16.98(16.52,17.44) | 270.41 | 4.08(3.42) |
| Nasopharyngitis | 15 | 2.59(1.56,4.31) | 2.58(2.08,3.09) | 14.57 | 1.37(0.64) |
| Hypersensitivity | 13 | 2.37(1.37,4.08) | 2.36(1.82,2.90) | 10.19 | 1.24(0.46) |
| Constipation | 13 | 2.00(1.16,3.46) | 2.00(1.45,2.54) | 6.49 | 1(0.22) |
| Heart rate decreased | 12 | 9.75(5.52,17.20) | 9.69(9.13,10.25) | 93.51 | 3.28(2.47) |
| Macular oedema | 10 | 72.96(39.12,136.07) | 72.58(71.96,73.20) | 701.93 | 6.17(5.3) |
| Bradycardia | 10 | 7.15(3.84,13.31) | 7.11(6.50,7.73) | 52.55 | 2.83(1.96) |
| Gastrointestinal disorder | 10 | 2.61(1.40,4.86) | 2.60(1.98,3.22) | 9.87 | 1.38(0.51) |
| Visual impairment | 9 | 2.29(1.19,4.4) | 2.28(1.63,2.93) | 6.49 | 1.19(0.27) |
| Migraine | 9 | 3.07(1.59,5.91) | 3.06(2.41,3.71) | 12.48 | 1.61(0.7) |
| Frequent bowel movements | 9 | 9.16(4.76,17.63) | 9.12(8.46,9.77) | 65.02 | 3.19(2.27) |
| Defaecation urgency | 9 | 33.95(17.62,65.41) | 33.79(33.14,34.44) | 285.66 | 5.07(4.16) |
| Chest pain | 9 | 2.14(1.11,4.12) | 2.13(1.48,2.79) | 5.44 | 1.09(0.18) |
| Diabetic retinopathy | 9 | 109.68(56.82,211.73) | 109.16(108.51,109.82) | 956.29 | 6.76(5.84) |
| Inflammation | 8 | 4.29(2.14,8.6) | 4.28(3.59,4.97) | 20.12 | 2.1(1.13) |
| Palpitations | 8 | 2.73(1.36,5.47) | 2.73(2.03,3.42) | 8.76 | 1.45(0.48) |
| Gastrooesophageal reflux disease | 7 | 3.35(1.59,7.04) | 3.34(2.60,4.08) | 11.50 | 1.74(0.72) |
| Mucous stools | 7 | 33.5(15.93,70.43) | 33.38(32.64,34.12) | 219.29 | 5.06(4.03) |
| Haemorrhage | 7 | 2.40(1.14,5.04) | 2.40(1.66,3.14) | 5.70 | 1.26(0.24) |
| Dyspepsia | 6 | 2.32(1.04,5.18) | 2.32(1.52,3.12) | 4.51 | 1.21(0.12) |
| Lymphocyte count decreased | 6 | 9.40(4.22,20.96) | 9.37(8.57,10.17) | 44.86 | 3.23(2.13) |
| Eye disorder | 6 | 6.23(2.80,13.90) | 6.22(5.42,7.02) | 26.27 | 2.64(1.54) |
| Thyroid disorder | 5 | 10.80(4.49,25.98) | 10.77(9.89,11.65) | 44.29 | 3.43(2.25) |
| Blood cholesterol abnormal | 5 | 37.85(15.71,91.16) | 37.75(36.87,38.63) | 178.36 | 5.23(4.05) |
| Flatulence | 5 | 3.39(1.41,8.15) | 3.38(2.51,4.26) | 8.39 | 1.76(0.58) |
| Influenza like illness | 5 | 3.02(1.26,7.27) | 3.02(2.14,3.89) | 6.74 | 1.59(0.41) |
| Blood pressure abnormal | 5 | 7.40(3.08,17.80) | 7.38(6.51,8.26) | 27.59 | 2.88(1.7) |
| Vertigo | 4 | 2.72(1.02,7.26) | 2.72(1.74,3.70) | 4.34 | 1.44(0.15) |
| Blood iron decreased | 4 | 9.35(3.51,24.95) | 9.33(8.36,10.31) | 29.75 | 3.22(1.93) |
| Haemorrhoids | 4 | 8.60(3.22,22.94) | 8.58(7.60,9.56) | 26.78 | 3.1(1.81) |
| Brain fog | 4 | 5.85(2.19,15.6) | 5.84(4.86,6.82) | 16.03 | 2.54(1.25) |
| Nephrolithiasis | 4 | 2.77(1.04,7.39) | 2.77(1.79,3.74) | 4.51 | 1.47(0.17) |
| Faecal calprotectin increased | 4 | 21.66(8.12,57.83) | 21.62(20.64,22.60) | 78.54 | 4.43(3.14) |
| Loss of therapeutic response | 4 | 24.39(9.14,65.12) | 24.34(23.36,25.32) | 89.36 | 4.6(3.31) |
| Poor quality sleep | 4 | 6.08(2.28,16.21) | 6.06(5.09,7.04) | 16.92 | 2.6(1.31) |
| Seasonal allergy | 4 | 7.21(2.70,19.25) | 7.20(6.22,8.18) | 21.35 | 2.85(1.55) |
| Bowel movement irregularity | 4 | 14.49(5.43,38.68) | 14.47(13.49,15.45) | 50.09 | 3.85(2.56) |
| Fungal infection | 3 | 3.14(1.01,9.74) | 3.13(2.00,4.26) | 4.36 | 1.65(0.2) |
| Liver function test increased | 3 | 3.46(1.11,10.73) | 3.45(2.32,4.58) | 5.23 | 1.79(0.34) |
| Iron deficiency anaemia | 3 | 13.33(4.29,41.41) | 13.31(12.18,14.45) | 34.14 | 3.73(2.29) |
| Drug effect less than expected | 3 | 3.86(1.24,11.98) | 3.85(2.72,4.98) | 6.34 | 1.95(0.5) |
| Night sweats | 3 | 3.17(1.02,9.84) | 3.17(2.04,4.30) | 4.45 | 1.66(0.22) |
| Escherichia infection | 3 | 12.08(3.89,37.52) | 12.07(10.94,13.20) | 30.42 | 3.59(2.15) |
| White blood cell count increased | 3 | 3.33(1.07,10.35) | 3.33(2.20,4.46) | 4.89 | 1.74(0.29) |
| Gout | 3 | 6.04(1.95,18.75) | 6.03(4.90,7.16) | 12.59 | 2.59(1.15) |
| Diarrhoea haemorrhagic | 3 | 11.23(3.62,34.88) | 11.22(10.09,12.35) | 27.90 | 3.49(2.04) |
| Gastritis | 3 | 4.77(1.54,14.82) | 4.77(3.64,5.90) | 8.93 | 2.25(0.81) |
| Vitamin d decreased | 3 | 12.88(4.15,40.00) | 12.86(11.73,13.99) | 32.79 | 3.68(2.24) |
| Herpes simplex | 3 | 19.17(6.17,59.56) | 19.14(18.01,20.28) | 51.52 | 4.26(2.81) |
