# Supplementary material for: Safety of ozanimod and etrasimod in ulcerative colitis: A disproportionality analysis of the Food and Drug Administration Adverse Event Reporting System database
Source: Medicine (Baltimore). 2026 Aug 21;105(34):e50262. doi: 10.1097/MD.0000000000050262 (PMC13502775; doi:10.1097/MD.0000000000050262)
Supplement: Supplementary file 14 [file medi-105-e50262-s014.docx]

**Supplementary Table S14. Demographic information,** **adverse events and outcomes of serious cases related to ozanimod and etrasimod.**

| **Ozanimod** | | | | | | |
| --- | --- | --- | --- | --- | --- | --- |
| **Primaryid** | **Outcome** | **Age** | **Sex** | **Time-to onset** | **PT** | **SOC** |
| 205181931 | Death | 59 | F | 7 | Multiple organ dysfunction syndrome | General disorders and administration site conditions |
| 205440811 | Death | 75 | M | missing | Death | General disorders and administration site conditions |
| 211304643 | Death | 70 | F | 0 | Dizziness | Nervous system disorders |
| 211304643 | Death | 70 | F | 0 | Thrombosis | Vascular disorders |
| 211304643 | Death | 70 | F | 0 | Arterial occlusive disease | Vascular disorders |
| 211304643 | Death | 70 | F | 0 | Haemorrhage | Vascular disorders |
| 211304643 | Death | 70 | F | 0 | Intestinal obstruction | Gastrointestinal disorders |
| 211304643 | Death | 70 | F | 0 | Pain | General disorders and administration site conditions |
| 211304643 | Death | 70 | F | 0 | Muscle spasms | Musculoskeletal and connective tissue disorders |
| 212347511 | Death | 93 | F | missing | Death | General disorders and administration site conditions |
| 212412121 | Death | 70 | F | missing | Colitis ulcerative | Gastrointestinal disorders |
| 213371832 | Death | 33 | M | 72 | Road traffic accident | Injury, poisoning and procedural complications |
| 213371832 | Death | 33 | M | 72 | Death | General disorders and administration site conditions |
| 213371832 | Death | 33 | M | 72 | Spinal disorder | Musculoskeletal and connective tissue disorders |
| 213371832 | Death | 33 | M | 72 | Spinal fracture | Injury, poisoning and procedural complications |
| 213371832 | Death | 33 | M | 72 | Paralysis | Nervous system disorders |
| 218035622 | Death | 48 | F | missing | Sudden death | General disorders and administration site conditions |
| 230637101 | Death | missing | missing | missing | Drug ineffective | General disorders and administration site conditions |
| 230637101 | Death | missing | missing | missing | Colitis ulcerative | Gastrointestinal disorders |
| 230637101 | Death | missing | missing | missing | Completed suicide | Psychiatric disorders |
| 231025471 | Death | 74 | F | 35 | Death | General disorders and administration site conditions |
| 231655431 | Death | 67 | M | missing | Death | General disorders and administration site conditions |
| 234779701 | Death | 70 | M | missing | Death | General disorders and administration site conditions |
| 235129851 | Death | 56 | F | missing | Death | General disorders and administration site conditions |
| 236172221 | Death | 75 | M | missing | Death | General disorders and administration site conditions |
| 248191301 | Death | 57 | F | missing | Death | General disorders and administration site conditions |
| 186857055 | Life-Threatening | 52 | M | 452 | Covid-19 pneumonia | Infections and infestations |
| 203113223 | Life-Threatening | 62 | F | 25 | Anuria | Renal and urinary disorders |
| 203113223 | Life-Threatening | 62 | F | 25 | Angioedema | Skin and subcutaneous tissue disorders |
| 208580921 | Life-Threatening | 72 | M | missing | Cardiac disorder | Cardiac disorders |
| 210956911 | Life-Threatening | 42 | F | 140 | Cerebrovascular accident | Nervous system disorders |
| 213449861 | Life-Threatening | 79 | M | missing | Hypotension | Vascular disorders |
| 217538053 | Life-Threatening | 34 | M | 26 | Myocardial infarction | Cardiac disorders |
| 219181181 | Life-Threatening | 58 | F | missing | Appendicitis | Infections and infestations |
| 219181181 | Life-Threatening | 58 | F | missing | Glucocorticoid deficiency | Endocrine disorders |
| 219792011 | Life-Threatening | 62 | F | 0 | Stress | Psychiatric disorders |
| 219792011 | Life-Threatening | 62 | F | 0 | Bacteroides bacteraemia | Infections and infestations |
| 219792011 | Life-Threatening | 62 | F | 0 | Pain | General disorders and administration site conditions |
| 219792011 | Life-Threatening | 62 | F | 0 | Colitis | Gastrointestinal disorders |
| 219792011 | Life-Threatening | 62 | F | 0 | Influenza like illness | General disorders and administration site conditions |
| 219792011 | Life-Threatening | 62 | F | 0 | Nausea | Gastrointestinal disorders |
| 219792011 | Life-Threatening | 62 | F | 0 | Intestinal obstruction | Gastrointestinal disorders |
| 226291082 | Life-Threatening | 59 | M | missing | Pulmonary embolism | Respiratory, thoracic and mediastinal disorders |
| 230574081 | Life-Threatening | missing | missing | missing | Lymphopenia | Blood and lymphatic system disorders |
| 230574081 | Life-Threatening | missing | missing | missing | Infection | Infections and infestations |
| 232942793 | Life-Threatening | 57 | F | 323 | Headache | Nervous system disorders |
| 232942793 | Life-Threatening | 57 | F | 323 | Dysarthria | Nervous system disorders |
| 232942793 | Life-Threatening | 57 | F | 323 | Confusional state | Psychiatric disorders |
| 232942793 | Life-Threatening | 57 | F | 323 | Hypertension | Vascular disorders |
| 233280001 | Life-Threatening | 23 | M | 24 | Faeces discoloured | Gastrointestinal disorders |
| 233280001 | Life-Threatening | 23 | M | 24 | Diarrhoea | Gastrointestinal disorders |
| 233280001 | Life-Threatening | 23 | M | 24 | Abdominal distension | Gastrointestinal disorders |
| 233280001 | Life-Threatening | 23 | M | 24 | Decreased appetite | Metabolism and nutrition disorders |
| 233280001 | Life-Threatening | 23 | M | 24 | Gastrointestinal inflammation | Gastrointestinal disorders |
| 233280001 | Life-Threatening | 23 | M | 24 | Illness | General disorders and administration site conditions |
| 233280001 | Life-Threatening | 23 | M | 24 | Haemorrhage | Vascular disorders |
| 233412121 | Life-Threatening | 79 | F | missing | Hypertension | Vascular disorders |
| 233412121 | Life-Threatening | 79 | F | missing | Cerebrovascular accident | Nervous system disorders |
| 249403602 | Life-Threatening | 60 | F | 522 | Acute myeloid leukaemia | Neoplasms benign, malignant and unspecified (incl cysts and polyps) |
| 249403602 | Life-Threatening | 60 | F | 522 | Leukopenia | Blood and lymphatic system disorders |
| 1799764612 | Life-Threatening | 62 | M | 617 | Sepsis | Infections and infestations |
| 1799764612 | Life-Threatening | 62 | M | 617 | Cholecystitis acute | Hepatobiliary disorders |
| 1799764612 | Life-Threatening | 62 | M | 617 | Peripheral ischaemia | Vascular disorders |
| 178469567 | Hospitalization | 48 | M | 341 | Adenocarcinoma of colon | Neoplasms benign, malignant and unspecified (incl cysts and polyps) |
| 188421123 | Hospitalization | 39 | M | 361 | Covid-19 | Infections and infestations |
| 188421123 | Hospitalization | 39 | M | 361 | Pulmonary embolism | Respiratory, thoracic and mediastinal disorders |
| 189577943 | Hospitalization | 60 | F | 527 | Covid-19 | Infections and infestations |
| 189970532 | Hospitalization | 47 | M | 60 | Covid-19 pneumonia | Infections and infestations |
| 191070261 | Hospitalization | 59 | F | 432 | Covid-19 pneumonia | Infections and infestations |
| 191226513 | Hospitalization | 54 | F | 437 | Hemiplegia | Nervous system disorders |
| 192654021 | Hospitalization | 22 | M | 596 | Appendicitis | Infections and infestations |
| 195337547 | Hospitalization | 74 | F | 4 | Feeling abnormal | General disorders and administration site conditions |
| 195337547 | Hospitalization | 74 | F | 4 | Atrial fibrillation | Cardiac disorders |
| 195337547 | Hospitalization | 74 | F | 4 | Blood pressure decreased | Investigations |
| 195337547 | Hospitalization | 74 | F | 4 | Peripheral swelling | General disorders and administration site conditions |
| 195337547 | Hospitalization | 74 | F | 4 | Haematochezia | Gastrointestinal disorders |
| 195337547 | Hospitalization | 74 | F | 4 | Dizziness | Nervous system disorders |
| 195337547 | Hospitalization | 74 | F | 4 | Hyperhidrosis | Skin and subcutaneous tissue disorders |
| 195337547 | Hospitalization | 74 | F | 4 | Fatigue | General disorders and administration site conditions |
| 195885151 | Hospitalization | 39 | M | 131 | Suspected covid-19 | Infections and infestations |
| 195885151 | Hospitalization | 39 | M | 131 | Pneumonia | Infections and infestations |
| 196816516 | Hospitalization | 57 | F | incorrect | Chest discomfort | General disorders and administration site conditions |
| 196816516 | Hospitalization | 57 | F | incorrect | Pain | General disorders and administration site conditions |
| 196816516 | Hospitalization | 57 | F | incorrect | Fatigue | General disorders and administration site conditions |
| 196816516 | Hospitalization | 57 | F | incorrect | Drug ineffective | General disorders and administration site conditions |
| 196816516 | Hospitalization | 57 | F | incorrect | Malaise | General disorders and administration site conditions |
| 198066222 | Hospitalization | 26 | M | 0 | C-reactive protein increased | Investigations |
| 198066222 | Hospitalization | 26 | M | 0 | White blood cell count increased | Investigations |
| 198066222 | Hospitalization | 26 | M | 0 | Pain | General disorders and administration site conditions |
| 198066222 | Hospitalization | 26 | M | 0 | Arthralgia | Musculoskeletal and connective tissue disorders |
| 198066222 | Hospitalization | 26 | M | 0 | Diarrhoea | Gastrointestinal disorders |
| 198066222 | Hospitalization | 26 | M | 0 | Pyrexia | General disorders and administration site conditions |
| 198287462 | Hospitalization | 60 | M | 27 | Gastrointestinal disorder | Gastrointestinal disorders |
| 198695695 | Hospitalization | 26 | M | missing | Seizure | Nervous system disorders |
| 198695695 | Hospitalization | 26 | M | missing | Pyrexia | General disorders and administration site conditions |
| 198695695 | Hospitalization | 26 | M | missing | Diarrhoea | Gastrointestinal disorders |
| 198786388 | Hospitalization | missing | F | incorrect | Hyperhidrosis | Skin and subcutaneous tissue disorders |
| 198786388 | Hospitalization | missing | F | incorrect | Cardiac flutter | Cardiac disorders |
| 198786388 | Hospitalization | missing | F | incorrect | Colitis ulcerative | Gastrointestinal disorders |
| 198786388 | Hospitalization | missing | F | incorrect | Dizziness | Nervous system disorders |
| 198786388 | Hospitalization | missing | F | incorrect | Drug ineffective | General disorders and administration site conditions |
| 198855411 | Hospitalization | 65 | M | 11 | Rectal haemorrhage | Gastrointestinal disorders |
| 198855411 | Hospitalization | 65 | M | 11 | Pneumonia | Infections and infestations |
| 199042361 | Hospitalization | 50 | F | 26 | Blood potassium decreased | Investigations |
| 199268791 | Hospitalization | 48 | F | 18 | Covid-19 pneumonia | Infections and infestations |
| 199439761 | Hospitalization | 30 | M | 24 | Vomiting | Gastrointestinal disorders |
| 199439761 | Hospitalization | 30 | M | 24 | Rectal haemorrhage | Gastrointestinal disorders |
| 199750473 | Hospitalization | 36 | M | 3 | Liver function test increased | Investigations |
| 200131725 | Hospitalization | missing | F | incorrect | Drug ineffective | General disorders and administration site conditions |
| 200131725 | Hospitalization | missing | F | incorrect | Gastrointestinal disorder | Gastrointestinal disorders |
| 200466271 | Hospitalization | missing | M | missing | Hepatic enzyme increased | Investigations |
| 201605332 | Hospitalization | 37 | F | 0 | Therapeutic product effect incomplete | General disorders and administration site conditions |
| 201605332 | Hospitalization | 37 | F | 0 | Gastrointestinal inflammation | Gastrointestinal disorders |
| 201705641 | Hospitalization | 71 | F | 57 | Aneurysm | Vascular disorders |
| 201705641 | Hospitalization | 71 | F | 57 | Transient ischaemic attack | Nervous system disorders |
| 201882831 | Hospitalization | missing | F | missing | Haemorrhage | Vascular disorders |
| 201882831 | Hospitalization | missing | F | missing | Cerebrovascular accident | Nervous system disorders |
| 201885441 | Hospitalization | 63 | F | 86 | Colitis ulcerative | Gastrointestinal disorders |
| 202021184 | Hospitalization | 21 | M | 23 | Diarrhoea haemorrhagic | Gastrointestinal disorders |
| 202021184 | Hospitalization | 21 | M | 23 | Headache | Nervous system disorders |
| 202021184 | Hospitalization | 21 | M | 23 | Pyrexia | General disorders and administration site conditions |
| 202021184 | Hospitalization | 21 | M | 23 | Nasopharyngitis | Infections and infestations |
| 202021184 | Hospitalization | 21 | M | 23 | Dehydration | Metabolism and nutrition disorders |
| 202244991 | Hospitalization | missing | M | missing | Haemorrhage | Vascular disorders |
| 202244991 | Hospitalization | missing | M | missing | Gastrointestinal disorder | Gastrointestinal disorders |
| 202463331 | Hospitalization | missing | M | incorrect | Nasal septum deviation | Respiratory, thoracic and mediastinal disorders |
| 203541851 | Hospitalization | missing | F | missing | Infection | Infections and infestations |
| 204060711 | Hospitalization | missing | M | missing | Pneumonia | Infections and infestations |
| 204317561 | Hospitalization | missing | F | 10 | Colitis ulcerative | Gastrointestinal disorders |
| 204476371 | Hospitalization | 49 | M | 1 | Drug ineffective | General disorders and administration site conditions |
| 204476371 | Hospitalization | 49 | M | 1 | Supraventricular tachycardia | Cardiac disorders |
| 204476371 | Hospitalization | 49 | M | 1 | Dehydration | Metabolism and nutrition disorders |
| 205084481 | Hospitalization | missing | M | missing | Sepsis | Infections and infestations |
| 205085101 | Hospitalization | 18 | F | 38 | Sinusitis | Infections and infestations |
| 205085101 | Hospitalization | 18 | F | 38 | Febrile neutropenia | Blood and lymphatic system disorders |
| 205085101 | Hospitalization | 18 | F | 38 | Candida infection | Infections and infestations |
| 205104701 | Hospitalization | 42 | M | 191 | Infection | Infections and infestations |
| 205481641 | Hospitalization | 54 | M | 1 | Thrombosis | Vascular disorders |
| 205810341 | Hospitalization | 57 | F | missing | Large intestine perforation | Gastrointestinal disorders |
| 205853271 | Hospitalization | 24 | M | 6 | Haemorrhage | Vascular disorders |
| 205853271 | Hospitalization | 24 | M | 6 | Pain | General disorders and administration site conditions |
| 206586391 | Hospitalization | 48 | M | 25 | Diarrhoea | Gastrointestinal disorders |
| 206586391 | Hospitalization | 48 | M | 25 | Drug ineffective | General disorders and administration site conditions |
| 206586391 | Hospitalization | 48 | M | 25 | Hospitalisation | Surgical and medical procedures |
| 206586391 | Hospitalization | 48 | M | 25 | Proctalgia | Gastrointestinal disorders |
| 206923571 | Hospitalization | missing | F | missing | Feeling abnormal | General disorders and administration site conditions |
| 206923571 | Hospitalization | missing | F | missing | Paraesthesia | Nervous system disorders |
| 207045291 | Hospitalization | 42 | M | 199 | Postoperative wound infection | Infections and infestations |
| 207485141 | Hospitalization | 80 | F | incorrect | Atrial fibrillation | Cardiac disorders |
| 207752172 | Hospitalization | 39 | M | 15 | Seizure | Nervous system disorders |
| 207805662 | Hospitalization | 44 | M | incorrect | Haematemesis | Gastrointestinal disorders |
| 207805662 | Hospitalization | 44 | M | incorrect | Palpitations | Cardiac disorders |
| 207805662 | Hospitalization | 44 | M | incorrect | Feeling abnormal | General disorders and administration site conditions |
| 207805662 | Hospitalization | 44 | M | incorrect | Depression | Psychiatric disorders |
| 207805662 | Hospitalization | 44 | M | incorrect | Haemoglobin decreased | Investigations |
| 207805662 | Hospitalization | 44 | M | incorrect | Rectal haemorrhage | Gastrointestinal disorders |
| 207805662 | Hospitalization | 44 | M | incorrect | Abdominal pain upper | Gastrointestinal disorders |
| 207805662 | Hospitalization | 44 | M | incorrect | Respiratory arrest | Respiratory, thoracic and mediastinal disorders |
| 207805662 | Hospitalization | 44 | M | incorrect | Arthralgia | Musculoskeletal and connective tissue disorders |
| 207805662 | Hospitalization | 44 | M | incorrect | Loss of consciousness | Nervous system disorders |
| 207805662 | Hospitalization | 44 | M | incorrect | Haematochezia | Gastrointestinal disorders |
| 207805662 | Hospitalization | 44 | M | incorrect | Anxiety | Psychiatric disorders |
| 207805662 | Hospitalization | 44 | M | incorrect | Heart rate increased | Investigations |
| 207805662 | Hospitalization | 44 | M | incorrect | Haemorrhoidal haemorrhage | Gastrointestinal disorders |
| 207805662 | Hospitalization | 44 | M | incorrect | Stress | Psychiatric disorders |
| 207805662 | Hospitalization | 44 | M | incorrect | Pollakiuria | Renal and urinary disorders |
| 207912123 | Hospitalization | 34 | M | missing | Back pain | Musculoskeletal and connective tissue disorders |
| 207912123 | Hospitalization | 34 | M | missing | Dehydration | Metabolism and nutrition disorders |
| 207968731 | Hospitalization | missing | F | missing | Hyperglycaemia | Metabolism and nutrition disorders |
| 207968731 | Hospitalization | missing | F | missing | Rash pruritic | Skin and subcutaneous tissue disorders |
| 207968731 | Hospitalization | missing | F | missing | Abdominal pain | Gastrointestinal disorders |
| 207968731 | Hospitalization | missing | F | missing | Haemorrhage | Vascular disorders |
| 208316532 | Hospitalization | 48 | M | missing | Back pain | Musculoskeletal and connective tissue disorders |
| 208316532 | Hospitalization | 48 | M | missing | Colitis ulcerative | Gastrointestinal disorders |
| 208316532 | Hospitalization | 48 | M | missing | Asthenia | General disorders and administration site conditions |
| 208316532 | Hospitalization | 48 | M | missing | Weight decreased | Investigations |
| 208316532 | Hospitalization | 48 | M | missing | Drug ineffective | General disorders and administration site conditions |
| 208648851 | Hospitalization | 25 | M | 177 | Inflammatory marker increased | Investigations |
| 208648851 | Hospitalization | 25 | M | 177 | Food poisoning | Gastrointestinal disorders |
| 208920372 | Hospitalization | 38 | M | 30 | Decreased immune responsiveness | Immune system disorders |
| 208920372 | Hospitalization | 38 | M | 30 | Pneumonia | Infections and infestations |
| 209020471 | Hospitalization | 34 | F | 6 | Perineal abscess | Infections and infestations |
| 209033591 | Hospitalization | 59 | F | 14 | Dizziness | Nervous system disorders |
| 209033591 | Hospitalization | 59 | F | 14 | Asthenia | General disorders and administration site conditions |
| 209033591 | Hospitalization | 59 | F | 14 | Chest pain | General disorders and administration site conditions |
| 209033591 | Hospitalization | 59 | F | 14 | Nausea | Gastrointestinal disorders |
| 209033591 | Hospitalization | 59 | F | 14 | Diarrhoea | Gastrointestinal disorders |
| 209149011 | Hospitalization | 65 | F | 13 | Dehydration | Metabolism and nutrition disorders |
| 209149011 | Hospitalization | 65 | F | 13 | Pain | General disorders and administration site conditions |
| 209149011 | Hospitalization | 65 | F | 13 | Abdominal pain upper | Gastrointestinal disorders |
| 209149011 | Hospitalization | 65 | F | 13 | Renal disorder | Renal and urinary disorders |
| 209168151 | Hospitalization | 24 | M | missing | Colitis ulcerative | Gastrointestinal disorders |
| 209886621 | Hospitalization | 54 | M | 1 | Thrombosis | Vascular disorders |
| 209886621 | Hospitalization | 54 | M | 1 | Pain in extremity | Musculoskeletal and connective tissue disorders |
| 209886621 | Hospitalization | 54 | M | 1 | Weight increased | Investigations |
| 209988923 | Hospitalization | 37 | F | 4 | Frequent bowel movements | Gastrointestinal disorders |
| 209988923 | Hospitalization | 37 | F | 4 | Pain | General disorders and administration site conditions |
| 209988923 | Hospitalization | 37 | F | 4 | Diarrhoea | Gastrointestinal disorders |
| 209988923 | Hospitalization | 37 | F | 4 | Colitis ulcerative | Gastrointestinal disorders |
| 209988923 | Hospitalization | 37 | F | 4 | Nausea | Gastrointestinal disorders |
| 209988923 | Hospitalization | 37 | F | 4 | Secretion discharge | General disorders and administration site conditions |
| 209988923 | Hospitalization | 37 | F | 4 | Abdominal pain upper | Gastrointestinal disorders |
| 209988923 | Hospitalization | 37 | F | 4 | Rectal haemorrhage | Gastrointestinal disorders |
| 210105783 | Hospitalization | missing | M | 326 | Transient ischaemic attack | Nervous system disorders |
| 210667352 | Hospitalization | 57 | M | missing | Nausea | Gastrointestinal disorders |
| 210667352 | Hospitalization | 57 | M | missing | Haematoma | Vascular disorders |
| 210667352 | Hospitalization | 57 | M | missing | Pyrexia | General disorders and administration site conditions |
| 210667352 | Hospitalization | 57 | M | missing | Arthralgia | Musculoskeletal and connective tissue disorders |
| 210667352 | Hospitalization | 57 | M | missing | Splenic injury | Injury, poisoning and procedural complications |
| 210667352 | Hospitalization | 57 | M | missing | Gait disturbance | General disorders and administration site conditions |
| 210667352 | Hospitalization | 57 | M | missing | Hyperhidrosis | Skin and subcutaneous tissue disorders |
| 210667352 | Hospitalization | 57 | M | missing | Abdominal pain | Gastrointestinal disorders |
| 210748013 | Hospitalization | 49 | M | missing | Colitis ulcerative | Gastrointestinal disorders |
| 210748013 | Hospitalization | 49 | M | missing | Decreased appetite | Metabolism and nutrition disorders |
| 210748013 | Hospitalization | 49 | M | missing | Nephrolithiasis | Renal and urinary disorders |
| 210748013 | Hospitalization | 49 | M | missing | Taste disorder | Nervous system disorders |
| 210997692 | Hospitalization | 51 | M | 194 | Colitis ulcerative | Gastrointestinal disorders |
| 210997692 | Hospitalization | 51 | M | 194 | Product distribution issue | Product issues |
| 211511941 | Hospitalization | 45 | F | missing | Adverse event | General disorders and administration site conditions |
| 211672061 | Hospitalization | 27 | F | missing | Anal fistula | Gastrointestinal disorders |
| 211779641 | Hospitalization | 35 | F | missing | Mental disorder | Psychiatric disorders |
| 211921371 | Hospitalization | 62 | F | 221 | Flatulence | Gastrointestinal disorders |
| 211921371 | Hospitalization | 62 | F | 221 | Arthropathy | Musculoskeletal and connective tissue disorders |
| 211921371 | Hospitalization | 62 | F | 221 | Hypertension | Vascular disorders |
| 211921371 | Hospitalization | 62 | F | 221 | Blood pressure increased | Investigations |
| 211955112 | Hospitalization | 63 | M | 0 | Fatigue | General disorders and administration site conditions |
| 211955112 | Hospitalization | 63 | M | 0 | Colitis ulcerative | Gastrointestinal disorders |
| 211955112 | Hospitalization | 63 | M | 0 | Inappropriate schedule of product administration | Injury, poisoning and procedural complications |
| 211955621 | Hospitalization | missing | F | missing | Rectal haemorrhage | Gastrointestinal disorders |
| 211955621 | Hospitalization | missing | F | missing | Large intestine infection | Infections and infestations |
| 212368251 | Hospitalization | 0.5 | M | missing | Colitis ulcerative | Gastrointestinal disorders |
| 213573185 | Hospitalization | 59 | F | 115 | Immune reconstitution inflammatory syndrome | Immune system disorders |
| 213573185 | Hospitalization | 59 | F | 115 | Colitis ulcerative | Gastrointestinal disorders |
| 213573185 | Hospitalization | 59 | F | 115 | Leukopenia | Blood and lymphatic system disorders |
| 213899951 | Hospitalization | 76 | M | missing | Colitis ulcerative | Gastrointestinal disorders |
| 214092681 | Hospitalization | missing | F | missing | Ameloblastoma | Neoplasms benign, malignant and unspecified (incl cysts and polyps) |
| 214658264 | Hospitalization | 39 | F | 180 | Lymphopenia | Blood and lymphatic system disorders |
| 214658264 | Hospitalization | 39 | F | 180 | Cardiac failure | Cardiac disorders |
| 214658264 | Hospitalization | 39 | F | 180 | Myocarditis | Cardiac disorders |
| 214658264 | Hospitalization | 39 | F | 180 | Granulomatosis with polyangiitis | Vascular disorders |
| 214658264 | Hospitalization | 39 | F | 180 | Pulmonary embolism | Respiratory, thoracic and mediastinal disorders |
| 214658264 | Hospitalization | 39 | F | 180 | Gastritis | Gastrointestinal disorders |
| 214707981 | Hospitalization | 27 | M | 0 | Daydreaming | Psychiatric disorders |
| 214707981 | Hospitalization | 27 | M | 0 | Frequent bowel movements | Gastrointestinal disorders |
| 214707981 | Hospitalization | 27 | M | 0 | Confusional state | Psychiatric disorders |
| 214707981 | Hospitalization | 27 | M | 0 | Headache | Nervous system disorders |
| 214707981 | Hospitalization | 27 | M | 0 | Colitis ulcerative | Gastrointestinal disorders |
| 214707981 | Hospitalization | 27 | M | 0 | Clostridium difficile infection | Infections and infestations |
| 214707981 | Hospitalization | 27 | M | 0 | Somnolence | Nervous system disorders |
| 214707981 | Hospitalization | 27 | M | 0 | Fatigue | General disorders and administration site conditions |
| 215450721 | Hospitalization | 58 | M | missing | Dizziness | Nervous system disorders |
| 215450721 | Hospitalization | 58 | M | missing | Rhabdomyolysis | Musculoskeletal and connective tissue disorders |
| 215450721 | Hospitalization | 58 | M | missing | Hypersensitivity | Immune system disorders |
| 215450721 | Hospitalization | 58 | M | missing | Fall | Injury, poisoning and procedural complications |
| 215490491 | Hospitalization | 24 | M | 198 | Gastrointestinal disorder | Gastrointestinal disorders |
| 215767641 | Hospitalization | 61 | F | missing | Deep vein thrombosis | Vascular disorders |
| 215767641 | Hospitalization | 61 | F | missing | Dyspnoea | Respiratory, thoracic and mediastinal disorders |
| 215767641 | Hospitalization | 61 | F | missing | Pulmonary embolism | Respiratory, thoracic and mediastinal disorders |
| 216234102 | Hospitalization | 18 | M | missing | Hepatic enzyme abnormal | Investigations |
| 216234102 | Hospitalization | 18 | M | missing | White blood cell count decreased | Investigations |
| 216234102 | Hospitalization | 18 | M | missing | Colitis ulcerative | Gastrointestinal disorders |
| 217782201 | Hospitalization | 72 | F | missing | Pneumonia | Infections and infestations |
| 218063882 | Hospitalization | 21 | F | missing | Hypotension | Vascular disorders |
| 218063882 | Hospitalization | 21 | F | missing | Influenza | Infections and infestations |
| 218063882 | Hospitalization | 21 | F | missing | Migraine | Nervous system disorders |
| 218124381 | Hospitalization | 42 | F | 108 | Colitis ulcerative | Gastrointestinal disorders |
| 218124381 | Hospitalization | 42 | F | 108 | Product dose omission issue | Injury, poisoning and procedural complications |
| 218650268 | Hospitalization | 19 | F | incorrect | Posterior reversible encephalopathy syndrome | Nervous system disorders |
| 219664011 | Hospitalization | 87 | F | incorrect | Drug ineffective | General disorders and administration site conditions |
| 219664011 | Hospitalization | 87 | F | incorrect | Fall | Injury, poisoning and procedural complications |
| 219664011 | Hospitalization | 87 | F | incorrect | Urinary tract infection | Infections and infestations |
| 219664011 | Hospitalization | 87 | F | incorrect | Rib fracture | Injury, poisoning and procedural complications |
| 219664011 | Hospitalization | 87 | F | incorrect | Cervical vertebral fracture | Injury, poisoning and procedural complications |
| 219709071 | Hospitalization | missing | F | missing | Abdominal pain | Gastrointestinal disorders |
| 221360461 | Hospitalization | 35 | F | missing | Drug ineffective | General disorders and administration site conditions |
| 221445981 | Hospitalization | missing | F | missing | Vomiting | Gastrointestinal disorders |
| 221445981 | Hospitalization | missing | F | missing | Abdominal pain | Gastrointestinal disorders |
| 221446532 | Hospitalization | 67 | F | incorrect | Pulmonary oedema | Respiratory, thoracic and mediastinal disorders |
| 221446532 | Hospitalization | 67 | F | incorrect | Sepsis | Infections and infestations |
| 221446532 | Hospitalization | 67 | F | incorrect | Pneumonia | Infections and infestations |
| 222009061 | Hospitalization | 33 | M | missing | Gastrointestinal haemorrhage | Gastrointestinal disorders |
| 222009061 | Hospitalization | 33 | M | missing | Gastrointestinal pain | Gastrointestinal disorders |
| 222237962 | Hospitalization | 31 | F | 174 | Constipation | Gastrointestinal disorders |
| 223028723 | Hospitalization | 60 | M | 212 | Nystagmus | Nervous system disorders |
| 223028723 | Hospitalization | 60 | M | 212 | Product dose omission issue | Injury, poisoning and procedural complications |
| 223028723 | Hospitalization | 60 | M | 212 | Transient ischaemic attack | Nervous system disorders |
| 223028723 | Hospitalization | 60 | M | 212 | Skin infection | Infections and infestations |
| 223028723 | Hospitalization | 60 | M | 212 | Atrioventricular block first degree | Cardiac disorders |
| 223028723 | Hospitalization | 60 | M | 212 | Cerebrovascular accident | Nervous system disorders |
| 223028723 | Hospitalization | 60 | M | 212 | Hypertension | Vascular disorders |
| 223028723 | Hospitalization | 60 | M | 212 | Seizure | Nervous system disorders |
| 223028723 | Hospitalization | 60 | M | 212 | Speech disorder | Nervous system disorders |
| 226364601 | Hospitalization | 77 | F | 219 | Aphasia | Nervous system disorders |
| 226364601 | Hospitalization | 77 | F | 219 | Cerebrovascular accident | Nervous system disorders |
| 226577032 | Hospitalization | 73 | F | missing | Hospitalisation | Surgical and medical procedures |
| 226976822 | Hospitalization | 65 | F | 75 | Transient ischaemic attack | Nervous system disorders |
| 226976822 | Hospitalization | 65 | F | 75 | Epilepsy | Nervous system disorders |
| 227062382 | Hospitalization | 39 | F | 0 | Back pain | Musculoskeletal and connective tissue disorders |
| 227062382 | Hospitalization | 39 | F | 0 | Myalgia | Musculoskeletal and connective tissue disorders |
| 227062382 | Hospitalization | 39 | F | 0 | Condition aggravated | General disorders and administration site conditions |
| 227062382 | Hospitalization | 39 | F | 0 | Chest discomfort | General disorders and administration site conditions |
| 227062382 | Hospitalization | 39 | F | 0 | Pericardial effusion | Cardiac disorders |
| 227062382 | Hospitalization | 39 | F | 0 | Hypokalaemia | Metabolism and nutrition disorders |
| 227062382 | Hospitalization | 39 | F | 0 | Myopericarditis | Cardiac disorders |
| 227062382 | Hospitalization | 39 | F | 0 | Chest pain | General disorders and administration site conditions |
| 227062382 | Hospitalization | 39 | F | 0 | Hypersensitivity | Immune system disorders |
| 227062382 | Hospitalization | 39 | F | 0 | Troponin increased | Investigations |
| 227145071 | Hospitalization | 26 | M | missing | Gastrointestinal disorder | Gastrointestinal disorders |
| 228116602 | Hospitalization | 88 | F | missing | Depression | Psychiatric disorders |
| 228116602 | Hospitalization | 88 | F | missing | Fall | Injury, poisoning and procedural complications |
| 228525781 | Hospitalization | 42 | M | missing | Hospitalisation | Surgical and medical procedures |
| 228869211 | Hospitalization | 60 | M | missing | Colitis ulcerative | Gastrointestinal disorders |
| 228869211 | Hospitalization | 60 | M | missing | Drug ineffective | General disorders and administration site conditions |
| 228922911 | Hospitalization | 65 | F | 60 | Colitis ulcerative | Gastrointestinal disorders |
| 229215312 | Hospitalization | missing | F | missing | Gastrointestinal disorder | Gastrointestinal disorders |
| 229215312 | Hospitalization | missing | F | missing | Colitis ulcerative | Gastrointestinal disorders |
| 229469361 | Hospitalization | 19 | M | 77 | Colitis ulcerative | Gastrointestinal disorders |
| 229638261 | Hospitalization | missing | F | missing | Colitis ulcerative | Gastrointestinal disorders |
| 229821692 | Hospitalization | missing | F | missing | Hand fracture | Injury, poisoning and procedural complications |
| 229821692 | Hospitalization | missing | F | missing | Seizure | Nervous system disorders |
| 229981301 | Hospitalization | 69 | F | missing | Arrhythmia | Cardiac disorders |
| 230586022 | Hospitalization | 28 | M | 7 | Product dose omission issue | Injury, poisoning and procedural complications |
| 230586022 | Hospitalization | 28 | M | 7 | Vomiting | Gastrointestinal disorders |
| 230586022 | Hospitalization | 28 | M | 7 | Pulmonary embolism | Respiratory, thoracic and mediastinal disorders |
| 230586022 | Hospitalization | 28 | M | 7 | Nausea | Gastrointestinal disorders |
| 230586022 | Hospitalization | 28 | M | 7 | Chills | General disorders and administration site conditions |
| 231476052 | Hospitalization | missing | M | missing | Drug ineffective | General disorders and administration site conditions |
| 231476052 | Hospitalization | missing | M | missing | Colitis ulcerative | Gastrointestinal disorders |
| 232110591 | Hospitalization | 45 | M | missing | Hospitalisation | Surgical and medical procedures |
| 232195501 | Hospitalization | 20 | F | missing | Hospitalisation | Surgical and medical procedures |
| 232438321 | Hospitalization | 62 | F | 139 | Colitis ulcerative | Gastrointestinal disorders |
| 232870141 | Hospitalization | 30 | F | missing | Hospitalisation | Surgical and medical procedures |
| 233627133 | Hospitalization | 65 | M | missing | Carditis | Cardiac disorders |
| 235603652 | Hospitalization | 84 | M | missing | Loss of personal independence in daily activities | Social circumstances |
| 235603652 | Hospitalization | 84 | M | missing | Haematological infection | Infections and infestations |
| 235603652 | Hospitalization | 84 | M | missing | Pain | General disorders and administration site conditions |
| 237487451 | Hospitalization | 0.7 | M | missing | Haemoglobin decreased | Investigations |
| 237525361 | Hospitalization | 27 | M | missing | Influenza like illness | General disorders and administration site conditions |
| 237643671 | Hospitalization | 51 | M | 21 | Fatigue | General disorders and administration site conditions |
| 237643671 | Hospitalization | 51 | M | 21 | Gastritis | Gastrointestinal disorders |
| 237643671 | Hospitalization | 51 | M | 21 | Myocardial infarction | Cardiac disorders |
| 237856701 | Hospitalization | 27 | F | 383 | Hospitalisation | Surgical and medical procedures |
| 237944511 | Hospitalization | 72 | M | missing | Arrhythmia | Cardiac disorders |
| 238089171 | Hospitalization | 15 | F | missing | Hospitalisation | Surgical and medical procedures |
| 238280461 | Hospitalization | missing | missing | missing | Colitis ulcerative | Gastrointestinal disorders |
| 238415682 | Hospitalization | 43 | F | 0 | Abdominal pain | Gastrointestinal disorders |
| 238512142 | Hospitalization | 85 | F | missing | Urinary tract infection | Infections and infestations |
| 238512142 | Hospitalization | 85 | F | missing | Hallucination | Psychiatric disorders |
| 238512142 | Hospitalization | 85 | F | missing | Disorientation | Psychiatric disorders |
| 238512142 | Hospitalization | 85 | F | missing | Memory impairment | Nervous system disorders |
| 238512142 | Hospitalization | 85 | F | missing | Headache | Nervous system disorders |
| 240086112 | Hospitalization | 35 | M | 19 | Cytomegalovirus infection | Infections and infestations |
| 240086112 | Hospitalization | 35 | M | 19 | Dyspnoea | Respiratory, thoracic and mediastinal disorders |
| 240086112 | Hospitalization | 35 | M | 19 | Hypokalaemia | Metabolism and nutrition disorders |
| 240086112 | Hospitalization | 35 | M | 19 | Ventricular extrasystoles | Cardiac disorders |
| 240086112 | Hospitalization | 35 | M | 19 | Chest discomfort | General disorders and administration site conditions |
| 240819951 | Hospitalization | 65 | F | missing | Malaise | General disorders and administration site conditions |
| 240819951 | Hospitalization | 65 | F | missing | Therapy interrupted | Surgical and medical procedures |
| 240819951 | Hospitalization | 65 | F | missing | Bowel movement irregularity | Gastrointestinal disorders |
| 240819951 | Hospitalization | 65 | F | missing | Dehydration | Metabolism and nutrition disorders |
| 241478882 | Hospitalization | 15 | F | missing | Bone marrow disorder | Blood and lymphatic system disorders |
| 241478882 | Hospitalization | 15 | F | missing | Off label use | Injury, poisoning and procedural complications |
| 241953411 | Hospitalization | 18 | M | 68 | Sickle cell anaemia with crisis | Blood and lymphatic system disorders |
| 246414342 | Hospitalization | 57 | F | 615 | Cerebrovascular accident | Nervous system disorders |
| 246414342 | Hospitalization | 57 | F | 615 | Neoplasm malignant | Neoplasms benign, malignant and unspecified (incl cysts and polyps) |
| 246597461 | Hospitalization | 41 | F | 625 | Colitis ulcerative | Gastrointestinal disorders |
| 246783161 | Hospitalization | 71 | F | missing | Pneumonia | Infections and infestations |
| 249619471 | Hospitalization | 21 | M | missing | Colitis ulcerative | Gastrointestinal disorders |
| 249619471 | Hospitalization | 21 | M | missing | Norovirus infection | Infections and infestations |
| 249693811 | Hospitalization | 19 | M | 46 | Drug ineffective | General disorders and administration site conditions |
| 249693811 | Hospitalization | 19 | M | 46 | Colitis ulcerative | Gastrointestinal disorders |
| 1824901211 | Hospitalization | 60 | M | 296 | Anal abscess | Infections and infestations |
| 199551252 | Disability | 50 | M | 0 | Impaired work ability | Social circumstances |
| 199551252 | Disability | 50 | M | 0 | Drug ineffective | General disorders and administration site conditions |
| 199856193 | Disability | missing | F | incorrect | Eye disorder | Eye disorders |
| 199856193 | Disability | missing | F | incorrect | Blood pressure fluctuation | Vascular disorders |
| 199856193 | Disability | missing | F | incorrect | Headache | Nervous system disorders |
| 212985361 | Disability | 50 | F | incorrect | Illness | General disorders and administration site conditions |
| 212985361 | Disability | 50 | F | incorrect | Arthritis | Musculoskeletal and connective tissue disorders |
| 219630732 | Disability | 67 | F | missing | Drug ineffective | General disorders and administration site conditions |
| 232642281 | Disability | missing | M | missing | Eye disorder | Eye disorders |
| 236257502 | Disability | 62 | M | missing | Vision blurred | Eye disorders |
| **Etrasimod** | | | | | | |
| **Primaryid** | **Outcome** | **Age** | **Sex** | **Time-to onset** | **PT** | **SOC** |
| 230841251 | Hospitalization | 51 | F | 362 | Cytomegalovirus colitis | Infections and infestations |
| 233510793 | Hospitalization | 67 | M | 5 | Syncope | Nervous system disorders |
| 233510793 | Hospitalization | 67 | M | 5 | Gastrooesophageal reflux disease | Gastrointestinal disorders |
| 233510793 | Hospitalization | 67 | M | 5 | Drug ineffective | General disorders and administration site conditions |
| 233510793 | Hospitalization | 67 | M | 5 | Dyspnoea | Respiratory, thoracic and mediastinal disorders |
| 233510793 | Hospitalization | 67 | M | 5 | Vertigo | Ear and labyrinth disorders |
| 233510793 | Hospitalization | 67 | M | 5 | Dehydration | Metabolism and nutrition disorders |
| 233510793 | Hospitalization | 67 | M | 5 | Fatigue | General disorders and administration site conditions |
| 233510793 | Hospitalization | 67 | M | 5 | Dizziness | Nervous system disorders |
| 233510793 | Hospitalization | 67 | M | 5 | Bradycardia | Cardiac disorders |
| 233510793 | Hospitalization | 67 | M | 5 | Nausea | Gastrointestinal disorders |
| 233510793 | Hospitalization | 67 | M | 5 | Hypotension | Vascular disorders |
| 233510793 | Hospitalization | 67 | M | 5 | Large intestinal ulcer | Gastrointestinal disorders |
| 233510793 | Hospitalization | 67 | M | 5 | Diarrhoea | Gastrointestinal disorders |
| 234827681 | Hospitalization | 63 | F | incorrect | Speech disorder | Nervous system disorders |
| 234827681 | Hospitalization | 63 | F | incorrect | Headache | Nervous system disorders |
| 234827681 | Hospitalization | 63 | F | incorrect | Cerebrovascular accident | Nervous system disorders |
| 235601764 | Hospitalization | 56 | F | missing | Colitis | Gastrointestinal disorders |
| 235601764 | Hospitalization | 56 | F | missing | Colitis ulcerative | Gastrointestinal disorders |
| 235717354 | Hospitalization | 20 | F | 1 | Liver function test increased | Investigations |
| 235717354 | Hospitalization | 20 | F | 1 | Chest discomfort | General disorders and administration site conditions |
| 235717354 | Hospitalization | 20 | F | 1 | Bradycardia | Cardiac disorders |
| 235717354 | Hospitalization | 20 | F | 1 | Dizziness | Nervous system disorders |
| 235767072 | Hospitalization | 20 | F | 1 | Electrocardiogram pr shortened | Investigations |
| 235767072 | Hospitalization | 20 | F | 1 | Chest discomfort | General disorders and administration site conditions |
| 235767072 | Hospitalization | 20 | F | 1 | Bradycardia | Cardiac disorders |
| 235767072 | Hospitalization | 20 | F | 1 | Sinus bradycardia | Cardiac disorders |
| 239428782 | Hospitalization | 24 | M | missing | Therapeutic product effect decreased | General disorders and administration site conditions |
| 239428782 | Hospitalization | 24 | M | missing | Haemoglobin decreased | Investigations |
| 240857191 | Hospitalization | missing | M | missing | Colitis ulcerative | Gastrointestinal disorders |
| 241483341 | Hospitalization | missing | M | missing | Drug ineffective | General disorders and administration site conditions |
| 241896482 | Hospitalization | 70 | F | incorrect | Pneumonia | Infections and infestations |
| 241947842 | Hospitalization | 55 | F | incorrect | Chills | General disorders and administration site conditions |
| 241947842 | Hospitalization | 55 | F | incorrect | Condition aggravated | General disorders and administration site conditions |
| 241947842 | Hospitalization | 55 | F | incorrect | Pain | General disorders and administration site conditions |
| 241947842 | Hospitalization | 55 | F | incorrect | Pyrexia | General disorders and administration site conditions |
| 242637703 | Hospitalization | 37 | M | 70 | Clostridium difficile infection | Infections and infestations |
| 242637703 | Hospitalization | 37 | M | 70 | Escherichia infection | Infections and infestations |
| 242637703 | Hospitalization | 37 | M | 70 | Condition aggravated | General disorders and administration site conditions |
| 245398711 | Hospitalization | 47 | F | 4 | Pain | General disorders and administration site conditions |
| 245398711 | Hospitalization | 47 | F | 4 | Malaise | General disorders and administration site conditions |
| 245398711 | Hospitalization | 47 | F | 4 | Condition aggravated | General disorders and administration site conditions |
| 247137424 | Hospitalization | 62 | F | 7 | Product dose omission issue | Injury, poisoning and procedural complications |
| 247137424 | Hospitalization | 62 | F | 7 | Rectal haemorrhage | Gastrointestinal disorders |
| 247137424 | Hospitalization | 62 | F | 7 | Haemorrhoids | Gastrointestinal disorders |
| 247137424 | Hospitalization | 62 | F | 7 | Hypotension | Vascular disorders |
| 247170542 | Hospitalization | missing | F | missing | Palpitations | Cardiac disorders |
| 247774142 | Hospitalization | 59 | F | 75 | Acute myocardial infarction | Cardiac disorders |
| 247774142 | Hospitalization | 59 | F | 75 | Pericarditis | Cardiac disorders |
| 247827131 | Hospitalization | 75 | F | incorrect | Gastrointestinal disorder | Gastrointestinal disorders |
| 247887303 | Hospitalization | 41 | M | 3 | Rectal abscess | Infections and infestations |
| 248695061 | Hospitalization | missing | M | missing | Fall | Injury, poisoning and procedural complications |
| 248695061 | Hospitalization | missing | M | missing | Dizziness | Nervous system disorders |
| 248754461 | Hospitalization | missing | M | 2 | Loss of consciousness | Nervous system disorders |
| 248754461 | Hospitalization | missing | M | 2 | Fall | Injury, poisoning and procedural complications |
| 249570043 | Hospitalization | 34 | F | incorrect | Oropharyngeal pain | Respiratory, thoracic and mediastinal disorders |
| 249570043 | Hospitalization | 34 | F | incorrect | Bacterial vaginosis | Infections and infestations |
| 249570043 | Hospitalization | 34 | F | incorrect | Blood test abnormal | Investigations |
| 249570043 | Hospitalization | 34 | F | incorrect | Colitis ulcerative | Gastrointestinal disorders |
| 249570043 | Hospitalization | 34 | F | incorrect | Nasal congestion | Respiratory, thoracic and mediastinal disorders |
| 249570043 | Hospitalization | 34 | F | incorrect | Nasopharyngitis | Infections and infestations |
| 249570043 | Hospitalization | 34 | F | incorrect | Cellulitis | Infections and infestations |
| 249570043 | Hospitalization | 34 | F | incorrect | Drug ineffective | General disorders and administration site conditions |
| 249768991 | Hospitalization | 60 | M | 17 | Cerebrovascular accident | Nervous system disorders |
| 250369981 | Hospitalization | 59 | M | 20 | Extrasystoles | Cardiac disorders |
| 250369981 | Hospitalization | 59 | M | 20 | Ventricular extrasystoles | Cardiac disorders |
| 239195191 | Disability | 69 | F | incorrect | Oxygen saturation decreased | Investigations |
| 239195191 | Disability | 69 | F | incorrect | Blister | Skin and subcutaneous tissue disorders |
| 239195191 | Disability | 69 | F | incorrect | Tachycardia | Cardiac disorders |
| 239195191 | Disability | 69 | F | incorrect | Rash | Skin and subcutaneous tissue disorders |
| 239195191 | Disability | 69 | F | incorrect | Fatigue | General disorders and administration site conditions |
| 239195191 | Disability | 69 | F | incorrect | Dyspnoea | Respiratory, thoracic and mediastinal disorders |
